# Impact of Solution {Ba^2+^}:{SO_4_^2–^} on Charge Evolution of Forming and Growing Barite (BaSO_4_) Crystals: A ζ—Potential Measurement Investigation

**DOI:** 10.1021/acsomega.3c03727

**Published:** 2023-11-07

**Authors:** Sergěj Y. M. H. Seepma, Bonny W. M. Kuipers, Mariëtte Wolthers

**Affiliations:** † Department of Earth Sciences, Utrecht University, Princetonlaan 8A, Utrecht 3584 CB, The Netherlands; ‡ Van ‘t Hoff Laboratory for Physical and Colloid Chemistry, Debye Institute for Nanomaterials Science, Utrecht University, Padualaan 8, Utrecht 3584 CH, The Netherlands

## Abstract

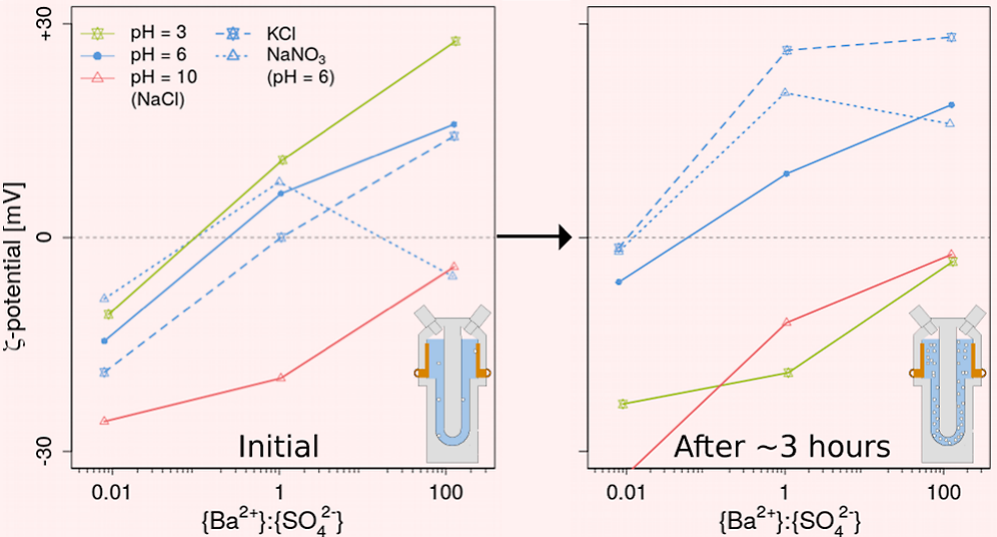

The impact of solution stoichiometry on formation of BaSO_4_ (barite) crystals and the development of surface charge was investigated at various predefined stoichiometries (*r*_aq_ = 0.01, 0.1, 1, 10, and 100, where *r*_aq_ = {Ba^2+^}:{SO_4_^2–^}). Synthesis experiments and zeta potential (ζ-potential) measurements were conducted at a fixed initial degree of supersaturation (Ω_barite_ = 1000, where Ω_barite_ = {Ba^2+^}{SO_4_^2–^}/*K*_sp_), at circumneutral pH of ∼6, 0.02 M NaCl, and ambient temperature and pressure. Mixed-mode measurement–phase analysis light scattering (M3-PALS) showed that the particles stayed negative for *r*_aq_ < 1 during barite crystal formation and positive for *r*_aq_ > 1. At *r*_aq_ = 1, two populations with a positive or negative ζ-potential prevailed for ∼2.5 h before a population with a circumneutral ζ-potential (−10 to +10 mV) remained. We relate the observations of particle charge evolution to particle size and morphology evolution under the experimental conditions. Furthermore, we showed that the ζ-potential became more negative when the pH was increased for every *r*_aq_. In addition, our results demonstrated that the type of monovalent background electrolyte did not influence the ζ-potential of barite crystals significantly, although NaCl showed slightly different behavior compared to KCl and NaNO_3_. Our results show the important role of surface charge (evolution) during ionic crystal formation under nonstoichiometric conditions. Moreover, our combined scanning electron microscopy and ζ-potential results imply that the surface charge during particle formation can be influenced by solution stoichiometry, besides the pH and ionic strength, and may aid in predicting the fate of barite in environmental settings and in understanding and improving industrial barite (surface chemistry) processes.

## 1 Introduction

BaSO_4_ (barite) mineral formation poses a major problem in the geothermal energy industry and during oil and gas recovery, where undesirable barite scale forms onto the surfaces of distribution piping and water handling equipment, such as pumps, valves, and heat transfer equipment.^1,2^ Additionally, the barite scale hinders the flow and adversely impacts the permeability of oil and gas reservoir rocks. On top of that, the barite scale is considered to be particularly difficult to deal with due to its low solubility and it contributes to about 80% of the total amount of scale deposits in the oil and gas business, ultimately leading to high treatment, repair, or additive-usage costs. Barite scale formation is often the result of mixing incompatible waters. For example, sulfate-rich seawater is often injected into offshore reservoirs for pressure maintenance, where it meets connate water, which often contains a high amount of barium. Furthermore, due to barite’s high specific gravity, whiteness, inertness, and opaqueness to X-ray characteristics, it plays an important role as filler for plastics, paints, rubbers, and pharmaceuticals.^3−5^

Multitudinous scientific studies have been dedicated to investigating the (rates of) nucleation and growth of barite on both the industrial level as well as from a fundamental perspective.^6−21^ In both settings, some of that research has been dedicated to the effect of stoichiometry (*r*_aq_; where *r*_aq_ = {Ba^2+^}:{SO_4_^2–^}) on bulk processes, like crystal growth rates,^6−9^ induction times,^10^ and nucleation.^9^ It was found that barite nucleation and growth depend strongly and asymmetrically on *r*_aq_ at a constant degree of supersaturation and pH ∼ 6. Barite formation was slower at extreme sulfate limitation than at equivalent barium-limited conditions. It is well-known that barite particle charge, in terms of zeta potential (ζ-potential), is affected by various physicochemical conditions (i.e., varying pH, ionic strength, chemical composition of solution matrix, temperature, and *r*_aq_) in aqueous solutions at or near equilibrium,^11−21^ but none have investigated the ζ-potential evolution from nonequilibrium to equilibrium conditions. In addition to the effect of *r*_aq_ on barite nucleation and growth rates, we expect that *r*_aq_ also has a significant impact on the surface charge development of Barite crystals during nucleation and growth, altering the crystal’s electrokinetic properties. In the latter properties, we include the isoelectric point (IEP), potential-determining ions (PDIs), potential indifferent ions (PIIs), surface potential (Ψ_s_), Stern potential (Ψ_d_), and ζ-potential among others, of barite crystals in aqueous solutions. These properties are important as they control flotation, coagulation, and dispersion characteristics in suspended systems,^5^ which in turn help to understand adsorption activation mechanisms,^22^ solid–liquid separation processes, including wastewater treatment systems^23,24^ and the optimal conditions of a well dispersed system.^25^

Therefore, we investigate the ζ-potential of barite particles during formation at different *r*_aq_ by using mixed-mode measurement-phase analysis light scattering (M3-PALS).^26^ We explore how the ζ-potential of the newly forming barite particles (i.e., in highly supersaturated conditions) changes while the particles grow toward stable crystals at (near-)equilibrium conditions. Moreover, we performed M3-PALS measurements to monitor the effect of different monovalent background electrolytes and pH on the ζ-potential at different *r*_aq_ in equilibrium and nonequilibrium conditions. We relate the observations of particle charge evolution to the particle size and morphology evolution under the same conditions. This experimental study is complementary to the investigations done by Seepma et al. (2023),^9^ and the measurements were conducted under the same conditions for which the *r*_aq_-dependence of barite nucleation and growth was reported.^9^

## 2 Materials and Methods

### 2.1 Growth Solutions

The formation of Barite from an aqueous solution was established by the following reaction
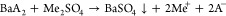
1where A = Cl or NO_3_ and Me = Na or K. Experimental conditions were selected to be identical to those of Seepma et al. (2023),^9^ who defined the optimal conditions for dynamic light scattering investigations of barite particle formation, including aspects of anisotropy, particle morphology, and sedimentation. Therefore, the target initial supersaturation was 1000 with respect to barite, i.e., Ω_barite_ = 1000, with Ω_barite_ defined as
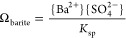
2where *K*_sp_ is the solubility product of barite (10^–9.99^ at 25 °C).^27−29^ The propriety of Ω_barite_ = 1000 is further touched upon in Supporting Information-I. To calculate the composition of different sets of growth solutions, with a range of *r*_aq_, pH, and ionic strength (*I*), Visual MINTEQ^30^—a free equilibrium speciation model-version 3.1—was used. Stock solutions of BaCl_2_, Ba(NO_3_)_2_, Na_2_SO_4_, K_2_SO_4_, NaCl, KCl, NaNO_3_, HCl, and NaOH, with different concentrations, were prepared by dissolving reagent grade salts into Milli-Q water (ISO 3696 Standard grade 1–18 mΩ) to create all growth solutions. Growth solutions of 50 mL were prepared from the stock solutions and kept in centrifuge tubes (Greiner), with a minimum amount of headspace, and were used to perform batch M3-PALS experiments within 48 h. Considering the volume of headspace and solution composition, no significant supersaturation with respect to BaCO_3_ (witherite) was reached due to potential CO_2_ incursion (see Supporting Information-I). The solution pH and *I* were adjusted by the addition of NaOH/HCl and NaCl/KCl/NaNO_3_ (Sigma-Aldrich), respectively. The growth solutions were made in sets, where one solution contained the sulfate salt together with the pH- and *I*-adjusting salts and the other solution contained the barium salt. An equal volume of both solutions was simultaneously added in a beaker, shaken for about 5 s to mix, extracted with a syringe, and loaded into the cell for ζ-potential measurements (Section 2.4.).

The desired growth solutions’ *I* for these experiments was 0.02 M, which was high enough to ensure that *I* remained approximately constant during precipitation (see Figure S1 in the Supporting Information-II), but low enough that ζ-potential measurements could be conducted where the background electrolyte (BE) did not dominate the measured systems. The Davies equation (valid for *I* ≤ 0.5 M) was used to calculate the ionic activities of the growth solutions.^31^

All calculations within Visual MINTEQ were done using the experimental temperature of 20 °C and assuming an open atmosphere for the neutral and more acidic growth solutions, while for the more alkaline (pH > 10) conditions, a closed atmosphere was assumed because the hydration and hydroxylation of CO_2_ in the solution are much slower under these circumstances (see also Supporting Information-I).^32,33^Table 1 lists all of the growth solutions used with their physicochemical parameter values for the M3-PALS experiments. It must be noted that the desired pH of the final growth solutions for solution nos. 4.1–4.3 and 5.1–5.3 was 7, but most likely, due to quick CO_2_ dissolution at circumneutral pH conditions (i.e., 5.5–7.4) in very dilute systems,^34^ the pH dropped quickly to ∼5.5–6.0 right after mixing the two growth solutions (see measured pH values in Table 1), and continued to drop more gradually to 5.1–5.2.

**Table 1:** Chemical Properties of the Investigated Growth Solutions^a^

	calculated parameters (MINTEQ)																	measured
solution no.	HCl/NaOH	BaCl_2_	Na_2_SO_4_	NaCl	{Ba^2+^}	{SO_4_^2–^}	{BaCl^+^}	{BaOH^+^}	{NaSO_4_^–^}	{HSO_4_^–^}	pH_ini_	pH_eq_	*I*_ini_	*I*_eq_	Ω_barite_	*r*_aq_	ø_s_	pH_ini_
2.1	0	0.0598	5.98	3.00	0.0274	3.27	10^–4^–10^–5^	<10^–7^	0.228	10^–4^–10^–5^	7.13	7.13	0.0205	0.0203	1002	0.008	0.00139	6.73
2.2	0	0.179	1.79	14.4	0.0920	0.970	0.00105	<10^–7^	0.0854	<10^–5^	7.09	7.09	0.0201	0.0194	997.7	0.095	0.00410	5.94
2.3	0	0.548	0.548	17.1	0.306	0.292	0.00414	<10^–6^	0.0251	<10^–5^	7.08	7.08	0.0201	0.0181	1000	1.047	0.0124	5.85
2.4	0	1.79	0.179	14.4	0.971	0.0921	0.0102	<10^–6^	0.00639	<10^–6^	7.08	7.08	0.0200	0.0194	1000	10.55	0.00410	5.86
2.5	0	5.90	0.0590	2.80	3.35	0.0267	0.0365	<10^–5^	0.000367	<10^–6^	7.08	7.08	0.0205	0.0203	1002	125.6	0.00138	5.82
3.1	1.17	0.560	0.560	15.8	0.313	0.285	0.00421	<10^–12^	0.0227	0.0242	3.00	2.99	0.0200	0.0180	997.7	1.099	0.0127	3.17
3.2	1.46	0.0610	6.10	1.50	0.0288	3.19	10^–4^–10^–5^	<10^–12^	0.204	0.271	3.00	3.00	0.0203	0.0201	1002	0.009	0.00142	3.18
3.3	1.15	6.00	0.0600	0.900	3.43	0.0261	0.0359	<10^–10^	0.000126	0.00222	3.00	3.00	0.0201	0.0199	1000	131.3	0.00140	3.22
3.4	–0.392	0.549	0.549	16.8	0.306	0.292	0.00407	0.000433	0.0252	<10^–9^	10.7	10.7	0.0202	0.0182	1000	1.046	0.0124	10.64
3.5	–0.428	0.0596	5.96	2.30	0.0273	3.28	10^–4^–10^–5^	10^–4^–10^–5^	0.224	<10^–8^	10.7	10.7	0.0202	0.0200	1000	0.008	0.00139	10.68
3.6	–0.400	5.87	0.0587	2.10	3.35	0.0267	0.0346	0.00484	0.000330	<10^–10^	10.7	10.7	0.0201	0.0199	1000	125.3	0.00137	10.64
	HCl/NaOH	BaCl_2_	K_2_SO_4_	KCl	{Ba^2+^}	{SO_4_^2–^}	{BaCl^+^}	{BaOH^+^}	{KSO_4_^–^}	{HSO_4_^–^}	pH_ini_	pH_eq_	*I*_ini_	*I*_eq_	Ω_barite_	*r*_aq_	ø_s_	pH_ini_
4.1	0	0.0600	6.00	3.00	0.0275	3.26	10^–4^–10^–5^	<10^–7^	0.269	10^–4^–10^–5^	7.13	7.13	0.0205	0.0203	1002	0.008	0.00140	6.09
4.2	0	0.550	0.550	17.0	0.307	0.291	0.00414	<10^–6^	0.0294	<10^–5^	7.08	7.08	0.0200	0.0180	1000	1.058	0.0125	5.78
4.3	0	5.88	0.0588	2.50	3.35	0.0267	0.0357	<10^–5^	0.000392	<10^–6^	7.08	7.08	0.0202	0.0200	1002	125.8	0.00137	5.65
	HCl/NaOH	Ba(NO_3_)_2_	Na_2_SO_4_	NaNO_3_	{Ba^2+^}	{SO_4_^2–^}	{BaNO_3_^+^}	{BaOH^+^}	{NaSO_4_^–^}	{HSO_4_^–^}	pH_ini_	pH_eq_	*I*_ini_	*I*_eq_	Ω_barite_	*r*_aq_	ø_s_	pH_ini_
5.1	0	0.0600	6.00	3.00	0.0272	3.28	0.000404	<10^–7^	0.229	10^–4^–10^–5^	7.13	7.13	0.0206	0.0204	1000	0.008	0.00140	6.20
5.2	0	0.560	0.560	16.9	0.299	0.299	0.0256	<10^–6^	0.0255	<10^–5^	7.08	7.08	0.0200	0.0180	1002	1.000	0.0127	5.80
5.3	0	6.00	0.0600	3.00	3.28	0.0273	0.230	<10^–5^	0.000402	<10^–6^	7.08	7.08	0.0206	0.0204	1000	120.4	0.00140	5.75

a Concentration/activity units are displayed in mmol/L; negative numbers in the second column indicate NaOH, positive numbers indicate HCl; Ø_s_ is the solid volume fraction [%]; and pH values were measured 5 min after mixing of the Ba-containing and SO_4_-containing growth solutions. In the experiments at pH 3 and 10, the pH changed <0.04 pH-units, while at circumneutral pH conditions, the pH dropped ±1 pH-unit, most likely by CO_2_ exchange with the atmosphere during sample handling, in combination with the low acid/base neutralizing capacity of those solutions.

### 2.2 Scanning Electron Microscopy Sample Preparation and Imaging

The barite particles formed during the ζ-potential measurements were characterized by scanning electron microscopy (SEM) imaging. 500 mL of each of the barite-containing solutions was filtered with 0.2 μm pore-size polycarbonate filters. Double-sided conductive carbon tabs were placed on standard aluminum stubs (with a diameter of 12.7 mm), and a piece of each filter, containing the samples, was cut and placed onto the tabs. The samples were coated with an 8 nm layer of 80:20 Pt/Pd coating. Subsequently, the samples were analyzed using a JEOL JCM-6000 Tabletop SEM. A voltage current of 10 to 15 keV was used.

### 2.3 Electrophoresis Theory

ζ-Potential [V] is a difficult parameter to measure and, for our type of suspensions, is usually acquired indirectly by measuring the electrophoretic mobility *u*_e_ [m^2^ V^–1^ s^–1^], which is the particle’s velocity divided by the electric field strength, under an applied electrical field. It is related to ζ-potential by Henry’s equation^35^ if the particles in the system can be assumed to be spherical
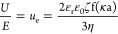
3where *E* is the electrical field [V m^–1^], *U* is the average velocity of the (charged) particles in the medium [m s^–1^], ε_r_ is the solvent’s relative dielectric permittivity [-], ε_0_ is the permittivity of free space [kg m V^–2^ s^–2^], η is the dynamic viscosity [kg m^–1^ s^–1^], *f*(κ*a*) is the Henry function, with κ [m^–1^] as the inverse Debye length, and *a* [m] as the particle radius. It is worth mentioning that, according to eq 2, *u*_e_ is more or less independent of the particle radius *a*. The value for the Henry function varies between 1 (Hückel approximation) and 1.5 (Smoluchowski approximation) and is determined by the product of the (average) particle size radius *a* to the inverse Debye length κ. Generally, ζ-potential measurements have a relatively large error compared to other techniques. The measurement accuracy for ζ-potential is at best ±2 mV or ±10%, which means that minor changes and the meaning of those observed behaviors in the ζ-potential cannot and should not be overinterpreted.^36^ We therefore focused our discussion on changes in the ζ-potential larger than 5 mV.

### 2.4 ζ-Potential Measurements

ζ-Potential batch experiments were conducted with the Zetasizer ULTRA using ZS XPLORER v1.2.0.91 software.^37,38^ Based on the work of Seepma et al. (2023),^9^ we performed our M3-PALS measurements in the forward detection angle (FWD) at 17° for our type of suspensions since the dominant particle size formed at our conditions is best measured at this angle. The experiments were conducted in DTS1080 folded capillary cells.^37^ In the Zetasizer software, the measurement procedure involved a group that contained a series of measurements that was repeated three times. In that group, one size measurement (i.e., dynamic light scattering; DLS) was performed, followed by ten ζ-potential measurements (in FWD) and ended with another size measurement. These size measurements were conducted using the backscattering detection angle (BSD) at 174.7° because DLS measurements at this angle are more capable of observing particles at different size classes concurrently compared to FWD. Therefore, we used these BSD measurements to investigate if the applied electric field had a substantial (unexpected) effect on the particle sizes themselves. Parallel to the ζ-potential batch experiments, DLS batch experiments were conducted in FWD so that we could assess ζ-potential evolution with respect to size evolution (or, more correctly hydrodynamic diameter, cf. Seepma et al. (2023)^9^).

The Auto Mode approach was used for ζ-potential measurements. Consequently, slow field reversal (SFR) and fast field reversal (FFR) (or electro-osmosis and electrophoresis) were conducted sequentially as our samples fulfilled the condition of *I* < 0.15 M (cutoff value). In this way, we were able to obtain a distribution for ζ-potential (compared to a single mean value). Each ζ-potential measurement consisted of 25 subruns, with no pause in between those subruns. A delay period of 300 s between each ζ-potential measurement was chosen in order to avoid excessive joule heating of the sample, which has an adverse effect on the gold-coated electrodes on both ends of the folded capillary cell.^38^ Further settings included automatic attenuation and voltage selection, although 150 V was consistently selected for the voltage selection by the Zetasizer. The viscosity of the medium was chosen as that of water (i.e., 0.001 kg m^–1^ s^–1^), corrected for the temperature of 20 °C, but not corrected for the salt(s) in the medium as this amount was assumed to have a negligible effect in our samples.^39,40^ The relative dielectric constant chosen for our experiments was 80.4.

## 3 Results and Discussion

### 3.1 Barite Particle Characterization

**Figure 1:**
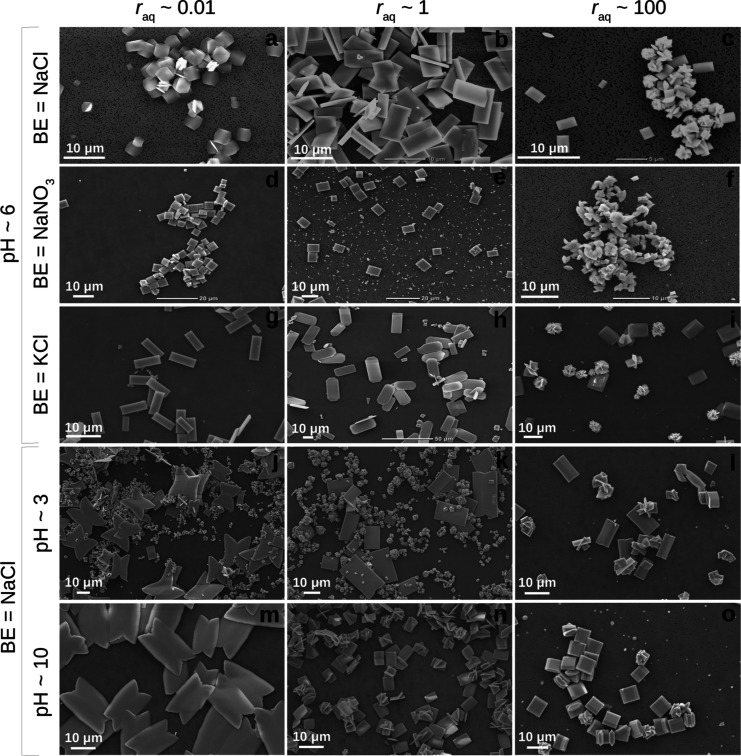
Morphology of the formed barite particles after approximately 3 h (i.e., near equilibrium). The figures correspond to the conditions listed in Table 1; *a* = (solution no.) 2.1, *b* = 2.3, *c* = 2.5, *d* = 5.1, *e* = 5.2, *f* = 5.3, *g* = 4.1, *h* = 4.2, *i* = 4.3, *j* = 3.2, *k* = 3.1, *l* = 3.3, *m* = 3.5, *n* = 3.4, and *o* = 3.6. The size indicator (white bar) is shown in the bottom-left and is 10 μm for each figure.

For the validity and interpretation of our ζ-potential measurements, we characterized for each condition at *r*_aq_ = 0.01, 1, and 100 (Table 1), the formed barite particles present at near-equilibrium (i.e., after 3 h) by SEM imaging (Figure 1). In addition, Figure S2 in Supporting Information-III shows SEM images for solution nos. 2.1–2.5. In all cases, our barite particles showed a platy-like/tabular morphology, without significant signs of 2D-nucleation on the largest and flat surface, which was likely the (100) crystal surface.^41−57^ Generally, at *r*_aq_ > 1, we observed that particles have a higher tendency for twinning and “rosette”-like crystal habit formation compared to *r*_aq_ ≤ 1. However, at pH ∼ 3, we observed that twinning of smaller-sized particles occurred on a broad scale, irrespective of *r*_aq_, while larger-sized particles did not show such twinning behavior. Lastly, our barite particles formed in the KCl solutions were slightly more elongated than those formed in the NaCl and NaNO_3_ solutions.

### 3.2 Validation of ζ-Potential Measurements during Particle Formation and Growth

ζ-Potential measurements are generally performed under stable (equilibrium) conditions. Contrastingly, our measurements were obtained during the dynamic process of particle formation and growth. Therefore, we first evaluated (1) the validity of the Smoluchowski limit of the Henry function, (2) the contribution of surface conductivity,^58^ (3) the aggregation and agglomeration behavior, (4) the extent of long-lasting structuring of particles due to packing, and (5) the role of sedimentation in our batch ζ-potential measurements.

**Figure 2:**
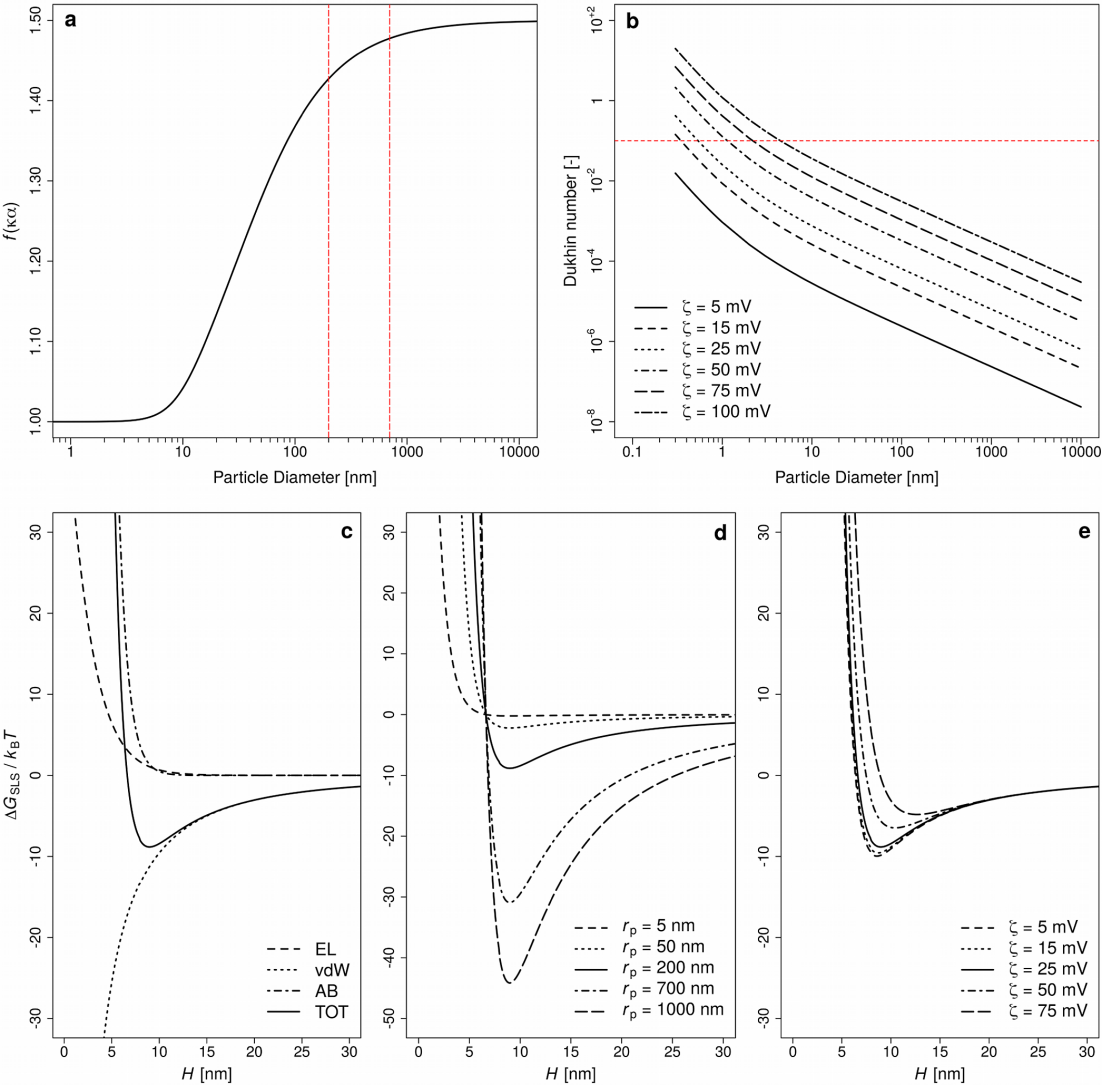
Value for the Henry function versus particle diameter, where the area between the red dashed lines indicates the particle sizes in our investigated systems (a), the Dukhin number versus particle diameter, where the red line indicates the threshold value (=0.1) (b), the hydration (AB), and the electrostatic (EL) and van der Waals (vdW) contributions to the total (TOT) Gibbs free energy field for particles with a diameter of 200 nm (c), the total Gibbs free energy field for different particle sizes for ζ-potential = 25 mV (d), and the total Gibbs free energy field for different ζ-potential at r_p_ = 200 nm (e).

**Figure 3:**
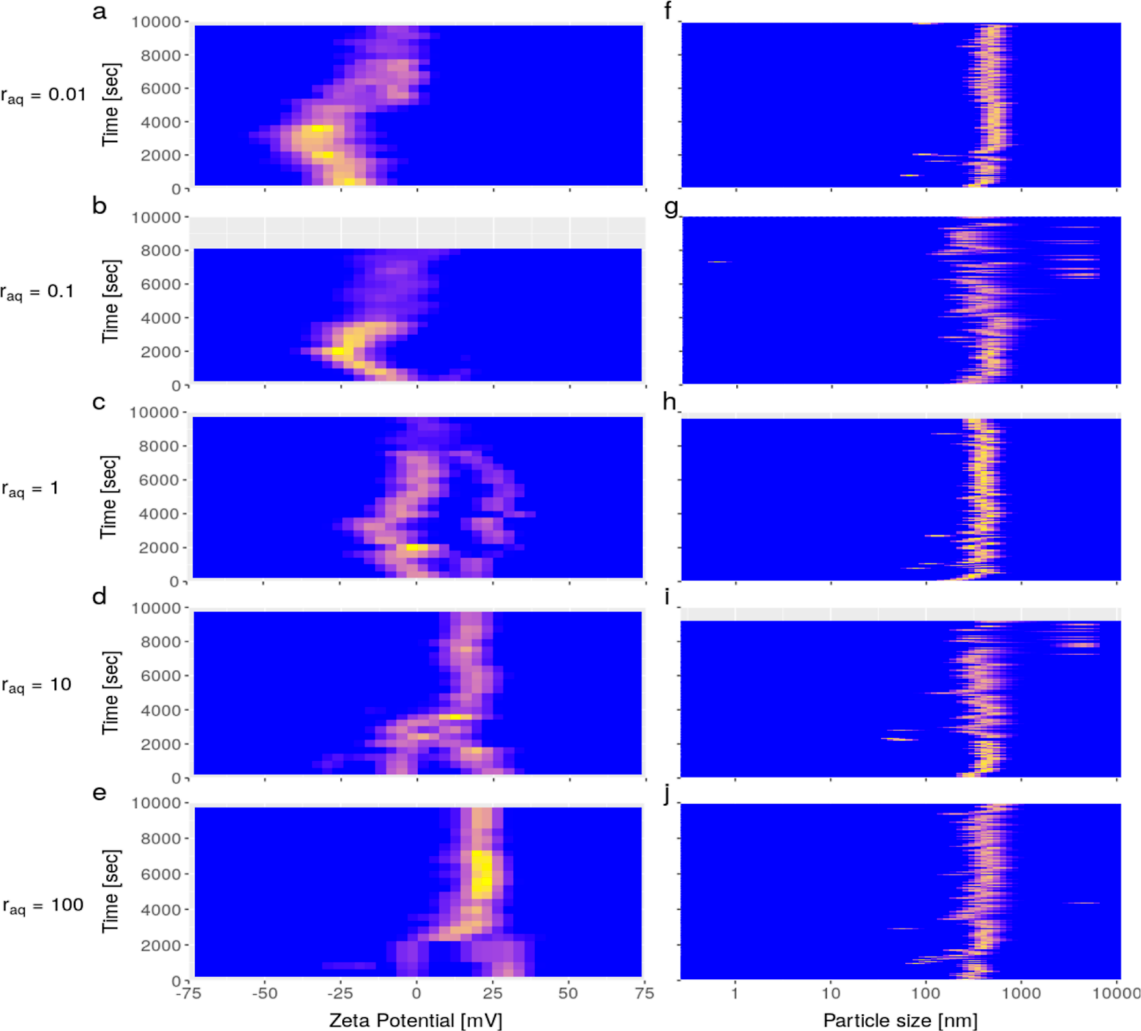
ζ-Potential evolution (a–e) and particle size evolution in FWD (f–j) for a set of *r*_aq_-values at initial Ω_barite_ = 1000. Results follow from experiments with solution nos. 2.1–2.5 (Table 1). Measurements have been conducted for roughly 3 h (up to 10,000 s). In (a–e), the total amount of counts is an absolute number and is indicated by the color, where blue indicates negligible and yellow indicates a large number of counts. The (linear) color scale across all of the panels was normalized to the measurement that contained the largest number of counts. However, the number particle size distributions (f-j) are inherently normalized. In other words, if the size distribution at a certain time contains only one population, which does not show a high degree of polydispersity, then it will always appear as “yellow” (f–j), even when a low total amount of particles is present.

**Figure 4:**
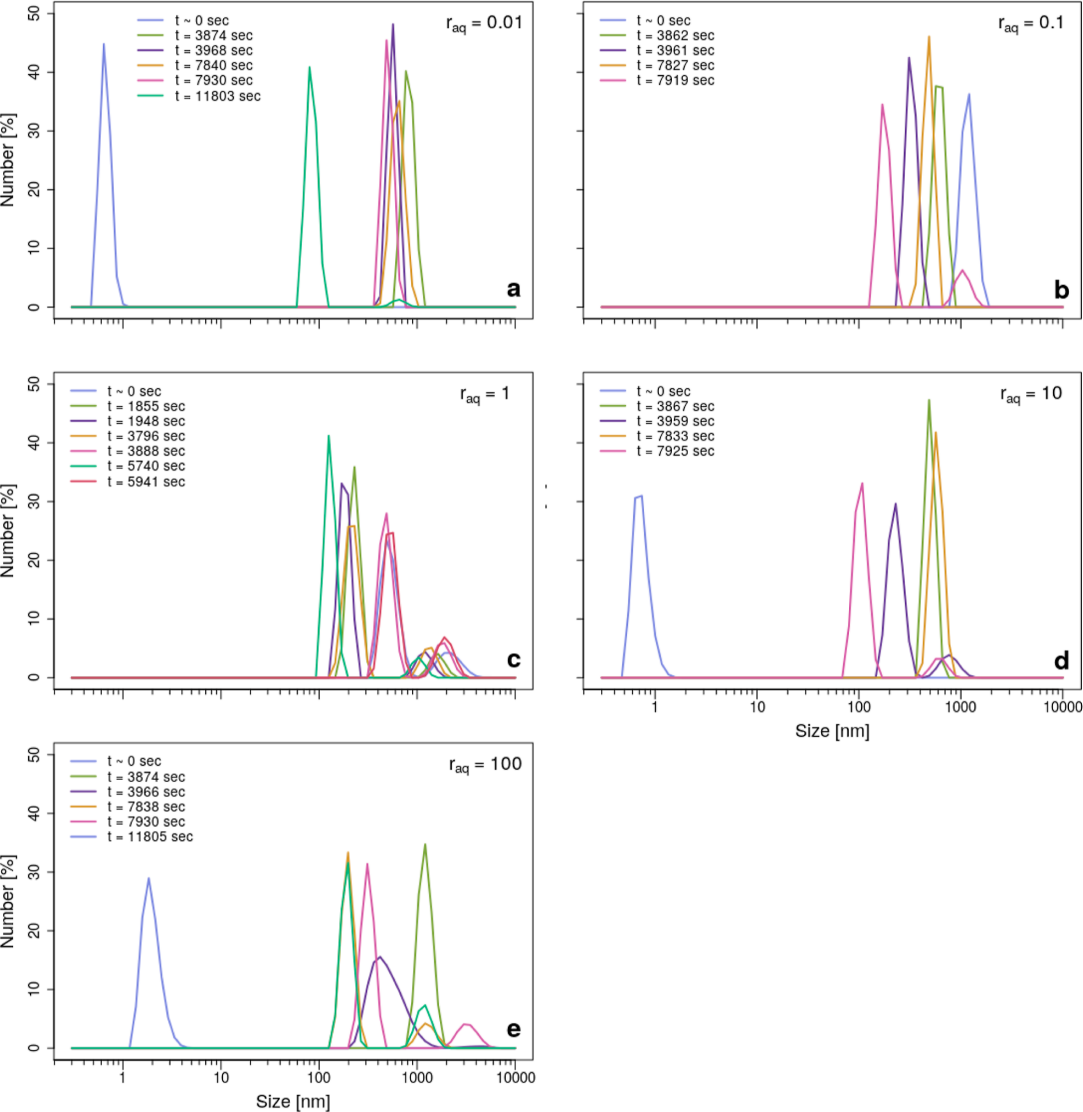
Evolution of the number of particles versus particle size by comparing BSD size data at certain time steps (see legends) obtained during the ζ-potential batch experiments at initial Ω_barite_ = 1000 for *r*_aq_ = 0.01 (a), *r*_aq_ = 0.1 (b), *r*_aq_ = 1 (c), *r*_aq_ = 10 (d), and *r*_aq_ = 100 (e).

First, due to the relatively high BE concentration, the ionic strength can be assumed to be constant in our experiments (Figure S1 in Supporting Information-II). Therefore, the Debye length can be assumed to be constant at 2.156 nm. Consequently, the Smoluchowski limit is valid for our commonly observed particles ≥ ∼ 100 nm (Figure 2a and S3 in Supporting Information-IV). Second, surface conductivity was determined non-negligible for the particles ≤10 nm (Figure 2b) that carried an absolute ζ-potential of ≥50 mV (Figure S4 in Supporting Information-V). Still, the particle sizes in our systems were generally >200 nm (Figure 3f–j and 4), which is in the range where ζ-potential measurements can be considered valid. Third, barite is known for its two hierarchical levels of aggregation, where particles with a size range of 5–10 nm aggregate into particles of 20–60 nm and subsequently into larger-sized crystals of 200–500 nm.^59^ Based on our DLS measurements (Figures 3f–j and 4), the measured ζ-potential must have represented the larger-sized particles, despite the fact that smaller-sized particles were initially present as well (i.e., at *t* = 0 in Figure 4). Therefore, aggregation was likely not a significant factor in the time frame of our ζ-potential measurements. Our SEM results show, with the exception of Figure 1h, single crystals or, at most, loosely bound crystals (likely due to the filtering process) were present in the measured systems. In some conditions, twinning of the crystals occurred and formed individual “rosettes”, which are generally not classified as agglomerates.^60^ In addition, the degree of agglomeration in our type of suspension was estimated (Supporting Information-VI). At the solid volume percentages for our experiments, the (average) interparticle distance was at least 413 nm and became larger as the (average) particle size increased. At such distances, the Gibbs free energy field for agglomeration is negligibly low (Figure 2c–e). Fourth, our calculations on the structure factor in our systems confirmed negligible effects of (long-lasting) particle–particle interactions due to packing (Supporting Information-VII). Finally, sedimentation of large particles occurred in some cases, demonstrated by a sudden drop in the absolute count rate in the ζ-potential measurements. However, the ζ-potential profiles with time (Figure 3a–e) did not show any discontinuities at the point where absolute count rates were strongly reduced (e.g., at *t* ∼ 8000 s in Figure 3a). In summary, the validations show that our ζ-potential measurements can be explained well in terms of surface chemistry development during particle formation. In Supporting Information-VIII, however, we discussed how these factors could have an influence on the ζ-potential if validations and observations would show otherwise.

### 3.3 Effect of Stoichiometry on ζ-Potential at Near Equilibrium

Figure 3 shows the time-resolved batch measurements (2–3 h) of the ζ-potential and particle size for a series of solutions that underwent barite formation, with varying *r*_aq_, 0.01, 0.1, 1, 10, and 100, at pH ∼ 5.5–7, *I* ∼ 0.02 M, and NaCl as BE. The Doppler phase plots and the voltage–current versus time plots for those experiments are shown in Figure S9 in the Supporting Information-IX, with the sole purpose of evaluation of data quality. In all experiments, the ζ-potential (Figure 3a–e) and particle size (Figure 3f–j) stabilized after an initial phase. The particle size distribution was systematically broader when *r*_aq_ ≠ 1 after the initial phase. It is worth noting that during batch barite formation Ω_barite_ quickly dropped before stabilizing near equilibrium. Consequently, it is likely that the stabilized ζ-potential and particle size reflect conditions approaching equilibrium. Also discernible in Figure 3a–e, is that the total amount of counts generally decreased with time.

At *r*_aq_ = 1 (Figure 3c), initially, two populations with a different ζ-potential were present. The population with the largest count rate had no net charge or a slightly negative charge. After approximately 8000 s (i.e., 133 min), when ∼ equilibrium was assumed, only one population persisted with a ζ-potential of ∼0 mV to slightly positive. This is in agreement with previous work at similar conditions and Gallardo et al. (2000)^17^ showed that the ζ-potential is approximately +5 mV (at *I* = 0.02 M, pH ∼ 6, *r*_aq_ = 1, and with NaCl as BE). Under these conditions, most other investigations reported slightly positive ζ-potential values as well^11,15,19^ and attributed this to the preferential release of SO_4_^2–^ from the barite crystal structure. Contrastingly, barite crystals in pure water (or with an *I* up to ∼0.001 M) were reported to carry a slightly negative charge,^12,13,17,18,20^ more comparable to our initial observations at *r*_aq_ = 1. These observations may reflect a different evolution of the surface structure of barite crystals. Buchanan and Heymann (1948)^11^ showed that natural barite has smooth surfaces, and synthesized or precipitated barite has very irregular surfaces and this may cause an absolute difference in ζ-potential as large as 20 mV.^61^ In most of the aforementioned research, the barite crystals were carefully selected before measuring ζ-potential, while in our measurements, this was not possible due to the nature of the experiments—we had no control over the geometry of the formed crystals or the surface roughness. Yet, our SEM images (Figure 1) confirmed that relatively similar barite crystals among all the different conditions (Table 1) were formed. Given the potential impact of particle (surface) structure on the ζ-potential, our results for *r*_aq_ = 1 are both within the literature range and within the error of previously reported values.

When *r*_aq_ < 1, at excess sulfate conditions, the ζ-potential was slightly negative (about −20 mV; Figure 3a), while the ζ-potential was slightly positive (about +25 mV; Figure 3e) when *r*_aq_ > 1, at excess barium. Although this has not been reported previously for nucleation experiments, it has been reported by numerous researchers for barite particles in barium or sulfate-excess conditions near equilibrium.^15,17,19,20,62−64^ Since the Ba^2+^-ions and SO_4_^2–^-ions in the solutions can adsorb specifically on the particle surface, their ratio in solution has a large influence on the surface potential and thus the ζ-potential (i.e., these are PDIs). Note that it has been shown previously that BEs such as Na^+^-ions and Cl^–^-ions can also interact with the particle surfaces.^65−71^ Under low ionic strength conditions (*I* < 0.1 M), such BE ions only affect the charging of the diffuse double layer and, in that case, are considered to act as indifferent ions, which means that they cannot cause a sign reversal for the surface charge but may affect and even reverse the sign of the ζ-potential^17,72−78^ (see also Section 3.6.). In our experiments, the {Na^+^}:{Cl^–^} is fairly constant (from 0.2 to 5) in the ionic strength buffer compared to the {Ba^2+^}:{SO_4_^2–^} (from 0.01 to 100) so it may be assumed that the impact of sodium and chloride on ζ-potential was more or less equal over *r*_aq_. Still, the magnitude of the ζ-potential at near-equilibrium may have been reduced and even reversed by Na^+^ or Cl^–^ ions as was observed toward the end of barite formation at *r*_aq_ = 0.01 (Figure 3a). To summarize, the negative surface charge in our sulfate-excess conditions is most likely caused by a lack of barium or an excess of sulfate adsorbing on the barite particle surfaces and vice versa for barium excess conditions, but the magnitude (and in specific cases, the sign) of the ζ-potential at near-equilibrium may have been affected by Na^+^ or Cl^–^ ions.

### 3.4 Effect of Stoichiometry on ζ-Potential Evolution Far from Equilibrium

While the ζ-potential values obtained at near equilibrium were within the range of previous research, our ζ-potential changed substantially with time before reaching equilibrium (i.e., in the first ∼4000 s or the first hour of the experiments, Figure 3). Foremost, we observed at *t* = 0 (initial measurements), a negative ζ-potential in excess sulfate and a positive ζ-potential in the initial population in excess barium. It is likely that this is caused by excess inner-sphere complexes of sulfate or barium, respectively. This supports the fact that barium and sulfate act as PDIs (Section 3.3.) and likely decide the sign of the ζ-potential, although other processes affecting the ζ-potential cannot be ruled out (Supporting Information-VIII).

Furthermore, in the first hour at every *r*_aq_, the ζ-potential changed considerably. Most notable is the ζ-potential at sulfate-excess conditions (*r*_aq_ < 1) that initially became more negative before becoming more neutral. Also striking are the two populations at *r*_aq_ = 10 and *r*_aq_ = 100, one with circumneutral particles and one population with more positive ζ-potential, that were present in the first hour or so. How this behavior relates to the different particle size classes observed will be discussed in Section 3.6. Furthermore, at *r*_aq_ = 10 (Figure 3d) and *r*_aq_ = 100 (Figure 3e), the largest number of counts was observed once these two initial populations became one population, after approximately 1 h as the systems evolved toward a more monomodal particle size distribution. From that point onward, the counts started to decrease slightly, likely due to the sedimentation (Supporting Information-VIII).

### 3.5 Particle Size Measurements

Time-resolved particle size measurements were conducted in two ways: (1) using BSD in sequence and in situ, i.e., between sets of ζ-potential measurements on the same suspensions and in the same cell as the ζ-potential measurements and (2) using FWD in parallel experiments at the same experimental conditions as those for the ζ-potential measurement. The latter data are shown in Figure 3f–j (see also Seepma et al. (2023)^9^). The predominant particle size observed for the different *r*_aq_ conditions was always between 100 and 700 nm (Figure 3f–j). In addition, the apparent size increased within the first half an hour at every *r*_aq_ (note that the scale in Figure 3f–j is logarithmic) from 200–300 nm to about 600–700 nm.

In the serial BSD size measurements (Figure 4), peaks occurred in a smaller size range than in the FWD data because BSD is more capable of monitoring multiple particle size populations in the samples compared to FWD (Seepma et al. (2023);^9^ reader is referred to Supporting Information-X for more background data). The initial measurement at *t* = 0 showed higher stochasticity (Figure 4a–e), where size distributions occurred at a much smaller size range than the generally observed 100–700 nm in FWD (Figure 3f–j) and this was also observed for the other conditions (Table 1) in serial BSD size measurements (Figure S15 in Supporting Information-XI). Most likely, the initial signal was more strongly influenced by nucleation besides particle growth. This is in agreement with the findings of MacHale and Finke (2023),^79^ who showed (based on the data of Turnbull (1953)^80^) that for initial Ω_barite_ = 361, nucleation dominated growth at *t* < 90 s. Though our initial Ω_barite_ was higher (i.e., Ω_barite_ = 1000), the difference in timing of nucleation at these high supersaturation values is small,^81−83^ i.e., induction time is less sensitive to the degree of supersaturation at Ω_barite_ > 316, it is likely that nucleation dominated growth at roughly *t* < 90 s in our (stoichiometric) systems with an initial Ω_barite_ = 1000 as well.

Based on Figures 3f–j and 4, it is likely that two particle size populations existed during the first hour at every *r*_aq_. However, at *t* ≫ 0 and every *r*_aq_, our BSD data are in agreement with our FWD measurements that were obtained without imposing an electric field. The slight differences in apparent sizes between Figures 3f–j and 4 can be explained by the different detection angles used (i.e., Mie Theory). Therefore, Figure 4 shows that during ζ-potential measurements, the particle size was not significantly affected by the imposed electric field.

### 3.6 Concomitant Particle Size and Charge Evolution with Varying Stoichiometry

At stoichiometric conditions (*r*_aq_ = 1), the apparent occurrence of two populations with different ζ-potential (Figure 3c) might be caused by the commonly known instability of uncharged particles in ζ-potential measurements.^84^ This “instability” is generally thought to reflect particle agglomeration. However, the BSD particle size measurements performed in sequence with the ζ-potential measurements also showed two populations (Figure 4c). Furthermore, both populations grew during the first 100 min (Figure 4c). The dominant population increased in average particle size from ∼150 to ∼500 nm, while the less dominant population grew from ∼1000 to ∼2000 nm. Simultaneously, the two populations observed in the ζ-potential measurements evolved via stronger (positive or negative) ζ-potentials toward uncharged particles.

From our results, it cannot be concluded unambiguously that the two differently sized populations at *r*_aq_ = 1 also represent the two populations with different ζ-potential. Nevertheless, what is clear from these measurements is that positively charged particles evolve more slowly to uncharged particles than negatively charged ones and that there is a difference in particle size (evolution) of the two populations. It might be that these two observations are related. For example, there may have been an initial preferential inner-sphere complex formation with either Ba^2+^ or SO_4_^2–^ between the different groups of particles, balanced over time by the adsorption of the other constituent ion. This process may have been slow and diffusion-limited in our nonstirred batch experiments. Alternatively, the evolution in ζ-potential for both groups of particles may reflect a different (change in) screening of the surface charge by various electrolyte ions, although such ions cannot trigger surface charge reversals^19,78^ (see also Section 3.3.). Both processes may also have occurred simultaneously. This suggests that particles that are Ba^2+^-enriched evolve and stabilize more slowly than SO_4_^2–^-enriched particles at *r*_aq_ = 1 conditions.

In excess sulfate conditions (*r*_aq_ = 0.01 and 0.1), generally a single population was observed in size and ζ-potential measurements. The negative ζ-potentials observed initially imply that the nuclei that formed likely carried an excess sulfate due to the adsorption of SO_4_^2–^ anions that formed inner-sphere complexes on the nuclei surfaces. The resulting ζ-potential value evolved to even more negative values for another 30–60 min, while the initial rapid particle size increase slowed down within 15 min (Figure 3a versus 3f and 4a and 3b versus 3g and 4b). Since no new <50 nm particles were observed after an hour, a stable 100–700 nm-sized population was present, it can be assumed that observed changes in the ζ-potential reflect the evolution of the particles’ surface and/or interface chemistry. Assuming that Ω_barite_ had dropped substantially at the point that no further increase in absolute ζ-potential was observed, crystal growth became more favorable than nucleation. This may explain the following decrease of ζ-potential. The absolute value of the ζ-potential is generally higher for small but stable (i.e., |ζ| ≥ 20) particle sizes compared to larger particle sizes (i.e., the value for *f*(κ*a*) changes; Figure 1a) because smaller particles are more affected by Brownian motion, where they tend to collide more easily with other particles.^85^ In addition, the amount of surface charge of the small particles is relatively larger than that of the large particles.^85^ Alternatively, it may be that particles that initially formed with a charge imbalance recrystallized/ripened and, in that process, approached stoichiometry and thereby charge balance. Lastly, the evolution toward a neutral ζ-potential may reflect an increase of Na^+^ ions in the diffuse layer that act as counterions for the SO_4_^2–^ ions absorbed to the crystal surface. Similar experiments to ours, but with CaSO_4_^75^ and CaCO_3_,^72,78^ showed that “indifferent” ions, like Na^+^ and Cl^–^, accumulate as counterions in the diffuse layer over a time of 30–60 min for agitated suspensions^61^ due to Coulombic attraction. It can be envisaged that for our non-agitated systems, this period was longer. Therefore, in our experiments (Figure 3a,b), the reduction of ζ-potential was likely caused by the diffuse layer being built up slowly with counterions (Na^+^) after the initial and more rapid adsorption of excess SO_4_^2–^ ions at the crystal surface.

At barium excess (*r*_aq_ = 10 and 100), our ζ-potential experiments showed behavior different from that at sulfate excess. Initially, two populations of particles with near-neutral and positive ζ-potentials were observed. With time, these converged toward a single population of less strongly positively charged particles. In these experiments, it is expected that the potential-determining ion is the Ba^2+^ ion, causing the positive ζ-potential values observed. The adsorption rate of Ba^2+^-ions is reportedly much slower than that of SO_4_^2–^-ions,^17,20^ most likely because the Ba^2+^-ion is more strongly hydrated and its dehydration is generally considered the rate-limiting step.^7,86−91^ Based on the observations of Derkani et al. (2019),^78^ it may be envisaged that, for *r*_aq_ > 1, the adsorption of Ba^2+^ ions at the crystal surfaces is on the same time scale as the movement of counterions toward the diffuse layer. Consequently, the two populations with initially different ζ-potentials (Figure 3d,e) may reflect this process. Unlike the case at *r*_aq_ < 1, the systems at *r*_aq_ > 1 contained initially uncharged (i.e., unstable within the context of ζ-potential) particles and it may be that the circumneutrally charged particles originated from nucleating crystals or even prenucleation clusters, exhibiting a high degree of aggregation. This type of behavior was also found for silica nanoparticles, which showed a bimodal ζ-potential distribution.^92^ Upon adsorbing excess barium after aggregation, these particles obtained a continuously more positive ζ-potential with time. This may be the cause for the trend observed at ∼2000–4000 s in both *r*_aq_ > 1 experiments (Figure 3d,e). Alternatively, the two ζ-potential populations may be related to the two different morphologies observed, especially at *r*_aq_ > 1: Singular platy-like crystals with a positive charge and individual “rosettes”. Although it is not known if twinning of platy-like crystals into “rosette-like” crystal structures affects the charge, the individual “rosettes” were most likely circumneutrally charged, based on our observations of the trend for the effect of *r*_aq_ on ζ-potential in combination with the trend in morphology with *r*_aq_. Yet, the two ζ-potential populations were only observed when “rosettes” were abundantly present (i.e., *r*_aq_ > 1). In any case, the population of particles (i.e., the larger-sized one), with a positive ζ-potential from the start, showed a slight decrease in ζ-potential with time. Similar to the *r*_aq_ < 1 systems, this can be explained by the slow build-up of counterion concentrations in the interface and/or crystallization/ripening toward stoichiometric crystal composition.

### 3.7 Conceptualization of the Stoichiometry Effect on Charge Development at Barite Surfaces

**Figure 5:**
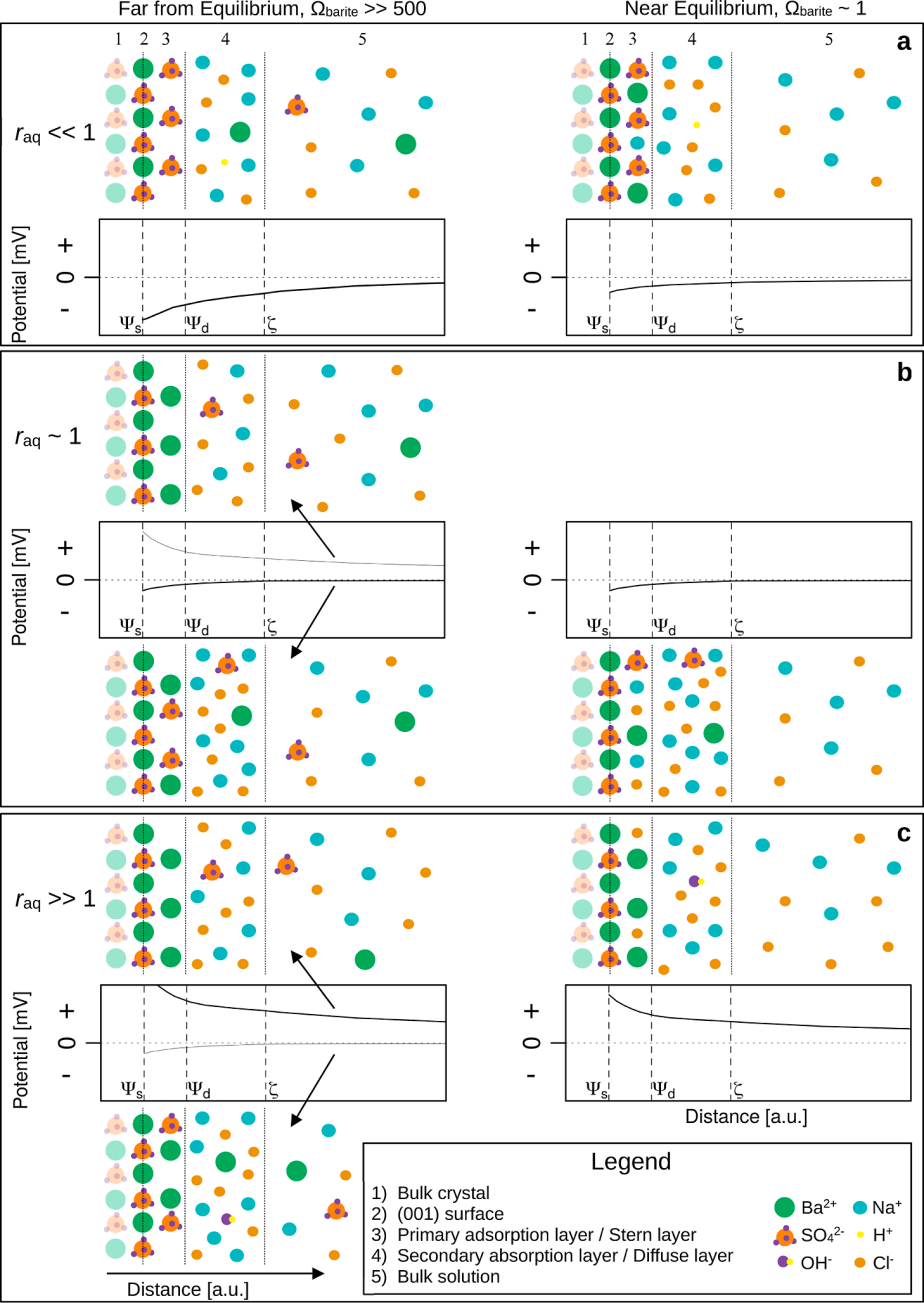
Conceptual models of particle–water interfaces of barite particles at *r*_aq_ ≪ 1 (a), *r*_aq_ ∼ 1 (b), and *r*_aq_ ≫ 1 (c), and conceptual plots of the (chemical) potential versus distance. On the left side of each figure, the initial and far from equilibrium situation is illustrated, while on the right side, the situation after approximately 3 h (i.e., near equilibrium) is shown. Ψ_s_, Ψ_d_, and ζ refer to the surface potential, Stern potential, and ζ-potential, respectively. For legibility reasons, water molecules were omitted.

The effect of *r*_aq_ on the particle charge evolution during barite formation is summarized conceptually in Figure 5. At *r*_aq_ ≪ 1 (Figure 5a), early in the formation, excess SO_4_^2–^ ions likely adsorbed onto the barite particles, forming a negatively charged primary adsorption layer, some of which may have been screened by increased concentrations of counterions like Na^+^ in the diffuse layer. We showed previously that particles formed initially in identical sulfate-excess conditions do not express crystal faces yet but rougher and rounded surfaces [transmission electron microscopy (TEM), Figure 3a in Seepma et al. (2023)^9^]. Toward equilibrium (i.e., after about 3 h in the experiments), barite has grown into more uniform and euhedral tabular crystals (Figure 1a), likely expressing stoichiometric (100) surfaces (i.e., with surface ions of Ba and SO_4_ at a 1:1 ratio). The adsorption and diffuse layers evolved with growing crystals and likely include only limited adsorption of SO_4_^2–^ ions, with some of this excess negative charge screened by counterions such as Na^+^ in the diffuse layer. Consequently, the ζ-potential evolved slowly over the 3 h from more to less negative due to the continued re-equilibration of the adsorbed and diffuse layers with the growing and (re)crystallizing particles.

At *r*_aq_ ∼ 1 (Figure 5b), early in the particle formation process, the dominant population of particles with a slightly negative to near-neutral charge suggests that slightly more SO_4_^2–^ ions than Ba^2+^ ions occupy the primary adsorption layer (and some of that charge is balanced by counterions such as Na^+^ in the diffuse layer). Contrastingly, the less dominant population of particles with a positive charge likely adsorbed more Ba^2+^ ions and, therefore, more Cl^–^ counterions were likely present in the diffuse layer. It is worth noting that the particles observed with TEM that formed within a few minutes at these experimental conditions (Seepma et al. (2023),^9^ their Figure 3c) were generally subhedral to euhedral, although some showed triangular or less crystalline morphologies. The differently charged populations observed in the ζ-potential measurements presented here may reflect these differently shaped particles that express more or less stoichiometric surfaces. Toward equilibrium, a single uncharged particle population remained. The SEM image of the particles present at these conditions (Figure 1b) shows a fairly homogeneous particle size and morphology. This indicates that the various particle morphologies observed initially have all grown to the most stable morphology for these conditions, expressing likely stoichiometric (100) faces, besides stoichiometric (001) and (010) faces, explaining why only uncharged particles remain.

At *r*_aq_ ≫ 1 (Figure 5c), similarly charged particle populations to those at *r*_aq_ ∼ 1 were observed early on during the formation, but now the positively charged particle population was more dominantly present, likely due to more adsorption of the excess Ba onto the particle surfaces. The particles observed initially at these identical conditions (Seepma et al. (2023),^9^ their Figure 3e) are a mixture of subhedral, triangular, and rounded particles. Toward equilibrium, these particles evolved into a mixture of tabular and rosette-like particles (Figure 1c) and a slightly less-positively charged particle population remained. Apparently, these particles still adsorb some of the excess barium, and probably, with the growth and morphology evolution of these particles, the composition of the Stern and diffuse layer also evolved in counterion concentrations, with lattice ions becoming more depleted and Cl^–^ concentration increasing.

### 3.8 Effect of pH

**Figure 6:**
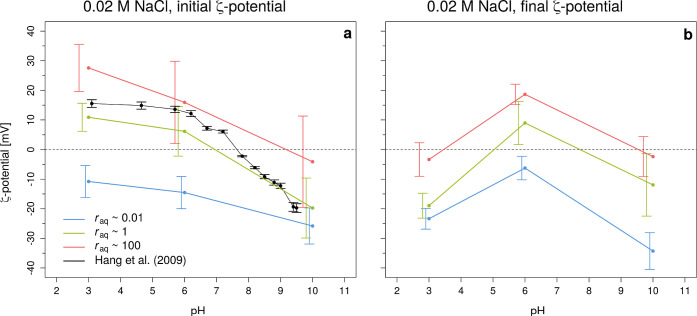
Measured (weighted average) ζ-potential versus pH at *r*_aq_ = 0.01, 1, and 100 at an initial Ω_barite_ = 1000 and in 0.02 M NaCl. At those conditions, the initial ζ-potential (i.e., within the first 3 min after growth solutions were added together) (a) and final ζ-potential (b) are displayed. Results found by Hang et al. (2009)^19^ are plotted in black with their error bars. Our colored error bars, obtained by a weighted standard deviation, are plotted next to the data points for clarity. Note that the lines are for illustration purposes and do not represent the actual evolution of the ζ-potential with pH (which is expected to be steeper/faster around the IEP of 7.8).

We investigated the effect of pH on the ζ-potential evolution for the BaSO_4_–NaCl–H_2_O batch experiments (solutions numbers 3.1–3.6 in Table 1) by measuring time-resolved ζ-potential at initial pH = 3, ∼6, and 10 at *r*_aq_ = 0.01, 1, and 100, respectively, while keeping the rest of the (initial) physicochemical parameters constant. The barite formation experiments lasted approximately 3 h (Figure S16 in the Supporting Information-XII). Note that the batch experiments are thought to evolve toward an equilibrium (Ω_barite_ ∼ 1) with time, whereby the solution stoichiometry becomes more extreme for the nonstoichiometric systems,^9^ while *r*_aq_ = 1 remains constant. Figure 6 summarizes the results, where Figure 6a shows the initial ζ-potential and Figure 6b shows the final ζ-potential at varying pH values and *r*_aq_.

Generally, we observed that the ζ-potential was (more) negative with an increase in pH at every *r*_aq_ in our initial measurements. A more negative ζ-potential with increasing pH has been observed in the past by other researchers as well^19,93−95^ and is ascribed to the adsorption of OH^–^ ions onto the positive charge centers of barite crystals and deprotonation of surface hydroxyl sites. Contrary to this, lowering the pH resulted in an increase in ζ-potential, due to OH^–^ desorption and H^+^ adsorption on the negative charge centers of barite crystals.^96^ Furthermore, other processes that may contribute to this trend are the distribution of dissolved lattice ions and/or hydrolytic reactions of H^+^ and OH^–^ with the adsorbed lattice ions at the solid–water interface.^19,93^ The point at which the amount of positive charges is equal to the amount of negative charges is known as the IEP.^97^ The IEP found by Hang et al. (2009)^19^ for barite lies approximately at pH = 7.8 (Figure 6a) and is best to compare our results for *r*_aq_ = 1, due to similar barite synthesis, comparable ionic strength, BE (i.e., *I* = 10^–2^ M NaCl), and type of analysis (also Malvern Zetasizer). However, other pH values have been found for the IEP. For example, Wierer and Dobiáš (1988)^93^ found that the IEP lies at pH ∼ 5.2, but they used natural barite (with a purity of 99.2%) from England (Cumberland), an ionic strength that was 1 order of magnitude lower than ours (i.e., *I* = 10^–3^ M NaCl) and was measured by a different apparatus (i.e., Marks II, Rank Bros. Cambridge). Zhang et al. (2011)^94^ found the IEP at pH = 6.92, but they grew their barite crystals in the presence of disodium ethylenediamine tetraacetate (EDTA-2Na) before the crystals were suspended in deionized water. The IEP that can be inferred from our results for *r*_aq_ = 1 is comparable to that reported by Hang et al. (2009),^19^ in particular when considering the usual sigmoidal nature of ζ-potential-pH dependence (instead of the trend lines in Figure 6). In addition to the general ζ-potential-pH dependence, we observed that the difference in ζ-potential between pH ∼ 6 and pH = 10 was larger than between pH = 3 and pH ∼ 6, in agreement with Hang et al. (2009)^19^ (Figure 6a). The pH also affected the general reproducibility of the ζ-potential values obtained. In particular, we observed a broader distribution of the ζ-potential at alkaline pH (i.e., pH = 10) compared to lower pH conditions for every *r*_aq_ as indicated by the larger error bars at that pH.

#### 3.8.1 ζ-Potential Evolution at *r*_aq_ = 1 at Different pH

At *r*_aq_ = 1, the ζ-potential followed the expected trend (Section 3.6.), with positive values at pH < pH_IEP_ and negative values at pH >pH_IEP_ (Figure 6a). The evolution of ζ-potential depended on pH and was most striking at pH = 3, where the ζ-potential reversed from +11 to −19 mV (i.e., Figure 6a versus Figure 6b). At the beginning of the experiment, nucleation was likely more favorable, causing a slightly positive ζ-potential. As time progressed and Ω_barite_ decreased, the new formation of barite crystals stopped while the particles continued to grow and ripen, and the electrical double layer (EDL) formation around the particles evolved. Due to the low solubility product of barite, the concentration of Ba^2+^ and SO_4_^2–^ ions in the solution near equilibrium was significantly lower compared to H^+^ ions at *r*_aq_ = 1 and pH = 3. This likely resulted in H^+^ (preferentially) filling up the Stern layer/primary adsorption layer at this pH (i.e., pH < pH_IEP_) and were adsorbed onto the negative charge centers of the formed barite crystals. Subsequently, a build-up of predominantly Cl^–^ likely occurred in the diffuse layer/secondary adsorption layer (cf. Williams (2016)^96^). Therefore, the ζ-potential (measured at the slipping plane) became more negative with time and its sign was reversed (cf. Zhang et al. (2011)^94^). At *r*_aq_ = 1 and pH = 6, the ζ-potential evolved from +6 to +9 mV and we thus did not observe a significant change in the ζ-potential between the initial and final measurement. This was likely due to the screening of the surface charge of both particle size populations with time (Section 3.3.). At *r*_aq_ = 1 and pH = 10, the ζ-potential became less negative by 8 mV only, meaning it stayed more or less constant and may be explained by faster barite formation due to much faster hydration-dehydration kinetics of the Ba^2+^ ions in the presence of OH^–^ ions.^98^ It is likely that barite crystal formation had already largely taken place before our first ζ-potential measurement.

#### 3.8.2 ζ-Potential Evolution at *r*_aq_ ≠ 1 at Different pH

At *r*_aq_ = 0.01, the general trend was offset to lower ζ-potentials, and the ζ-potential decrease with increasing pH was less strong (i.e., from −11 to −26 mV for *r*_aq_ = 0.01 versus +11 to −20 mV for *r*_aq_ = 1; Figure 6a). The more negative ζ-potential was most likely caused by the larger abundance of negative (SO_4_^2–^) charge centers at the surface, and the diffuse layer formed more rapidly with counterions compared to *r*_aq_ = 1 (Figure S16). At *r*_aq_ = 100, the ζ-potentials were somewhat more positive compared to *r*_aq_ = 1, where a ζ-potential was measured at +28 mV at pH = 3 going to −4 mV at pH = 10. The offset to larger positive ζ-potentials most likely reflects a larger abundance of potential-determining Ba^2+^ ions that are at the particles’ surface. At pH = 10, we expected that the role of the BaOH^+^ complex was (still) negligible as the activity is about 700 times less compared to that of Ba^2+^ (Table 1). Moreover, *r*_aq_ = 100 and pH = 10, we observed a “noisy” initial period (Figure S16i), which might be related to the delay of nucleation compared to *r*_aq_ = 0.01 and 1.^9^ In addition, as nucleation is slowed down, the supersaturation remained higher for a longer period, and therefore, a larger portion of the experiment involved simultaneously occurring processes of crystal nucleation events and crystal growth at *r*_aq_ = 100. Kuwahara et al. (2016)^99^ showed that for barite, spiral growth and nucleation can occur when Ω_barite_ = 300–1000 (we used Ω_barite_ = 1000). The trends observed at nonstoichiometric conditions agree with Forke et al. (1987)^15^ and Soccol et al. (2010),^20^ who previously showed that barium and sulfate are PDIs and therefore offset the ζ-potential-pH dependence and affect the IEP. For *r*_aq_ = 0.01 and 100, the ζ-potential decreased by 8 mV and increased by 2 mV, respectively (Figure 6a versus b), despite intermediate variability noticeable in Figure S16 in the Supporting Information-XII. However, these changes were within error (Section 3.6.).

### 3.9 Effect of Different Monovalent Background Electrolyte Types

**Figure 7:**
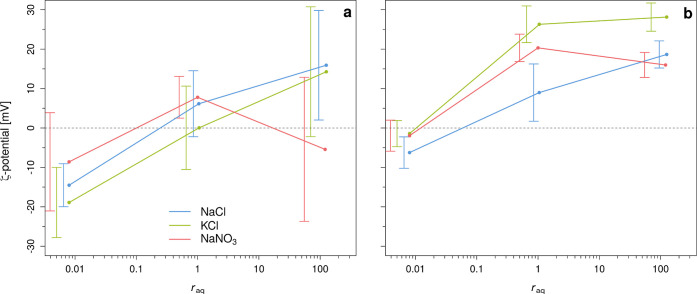
Measured (weighted average) ζ-potential versus *r*_aq_ for different monovalent BE solutions (NaCl, KCl, and NaNO_3_) at an initial Ω_barite_ = 1000 and in a 0.02 M salt solution. At those conditions, the initial ζ-potential (i.e., within the first 3 min after growth solutions were added together) (a) and final ζ-potential (b) are displayed. Error bars are plotted next to the data points for clarity. Note that the lines are for illustration purposes and do not represent the actual evolution of ζ-potential with *r*_aq_.

To investigate the impact of monovalent BE types on ζ-potential in our barite suspensions, we compared NaCl with KCl and NaNO_3_ at pH ∼ 6, thereby replacing once the anion and once the cation (solution nos. 2.1, 2.3, 2.5, 4.1–4.3, and 5.1–5.3 in Table 1). Figure S17 in Supporting Information-XIII shows the ζ-potential evolution of each of these conditions, while Figure 7 is a summary of those results, where Figure 7a shows the initial ζ-potential and Figure 7b shows the final ζ-potential at varying BE solutions and *r*_aq_. For the KCl and NaNO_3_ BE solutions, the ζ-potential evolved initially more chaotically at *r*_aq_ = 0.01 and 1, but especially for KCl at *r*_aq_ = 1. Notably in these solutions is the broader ζ-potential distribution (Figure S17a,b,g,h). This may be an indication that “diffusion broadening” played a role, where (early on) the diffusion of smaller-sized particles contributed relatively more to the ζ-potential distribution than the larger particles (which dominate at near equilibrium).^100^ In particular, the intensity particle size distributions for NaNO_3_ and KCl at *r*_aq_ = 1 (respectively, Figure S15a,e) were multimodal and more broad compared to NaCl (Figure S12). In addition, we cannot rule out that barite morphology may have changed in KCl and NaNO_3_ BE solutions more drastically compared to barite in NaCl BE solutions, which in that case could have contributed to broader ζ-potential distributions in the former. Additionally, although less likely due to the low conductivity in our samples (i.e., <2 mS/cm),^101^ we cannot rule out random factors, including higher local viscosity differences or increased electric field inhomogeneities, contributing to the initially more chaotic ζ-potential evolution for KCl and NaNO_3_ BE solutions.

#### 3.9.1 ζ-Potential Evolution at *r*_aq_ = 1 in Different Background Electrolytes

At *r*_aq_ = 1, we observed that the ζ-potential is initially slightly positive for all three BE solutions (i.e., +6, 0, and +8 mV, respectively, for NaCl, KCl, and NaNO_3_) and increased for all three BEs (i.e., to +9, +26, and +20 mV, respectively, for NaCl, KCl, and NaNO_3_). At *r*_aq_ = 1, for NaCl two distinct populations with a different ζ-potential persisted initially (Figure 3c), while this was not so discernible in both KCl (Figure S17b) and NaNO_3_ (Figure S17h) solutions. However, due to the observed “noisier” initial period for KCl and NaNO_3_, which lasted for about 30 min, we cannot rule out that there may have been two populations with a different ζ-potential present during the first 30 min. In addition, we observed that the ζ-potential in both KCl (Figure S17b) and NaNO_3_ (Figure S17h) solutions became more positive immediately after the start of the experiment, while in NaCl (Figure 3c), this trend only set in after approximately 1 h. This may imply that the saturation of the diffuse layer with counterions is delayed for NaCl solutions compared to KCl and NaNO_3_ solutions. This dissimilarity may hint toward differences in the structuring of water molecules in the solid–liquid interface due to the difference in ionic potential (defined as the electrical charge divided by the ionic radius) between Na^+^, K^+^, Cl^–^, and NO_3_^–^. In addition, Na^+^ has a slightly larger electronegativity than K^+^ (i.e., respectively, 0.9 and 0.8) and Cl^–^ has a much larger electronegativity than NO_3_^–^ (3.0 and 0.4 to 0.5, respectively). Therefore, the free Na^+^ and Cl^–^ ions likely form hydrogen bonds more strongly^102^ and may therefore be slower to diffuse and enter the EDL.^103^

#### 3.9.2 ζ-Potential Evolution at *r*_aq_ ≠ 1 in Different Background Electrolytes

At *r*_aq_ = 0.01, the general trend was offset to more negative values (i.e., to −15, −19, and −9 mV, respectively, for NaCl, KCl, and NaNO_3_) and, similarly, to *r*_aq_ = 1, the ζ-potential increased for all BEs compared to their initial values (i.e., to −6, −1, and −2 mV). Similarly to *r*_aq_ = 1, we observed that the ζ-potential in both KCl (Figure S17a) and NaNO_3_ (Figure S17g) solutions became more positive immediately after the start of the experiment, while in NaCl (Figure 3a), this trend only set in after approximately 1 h.

At *r*_aq_ = 100, the general trend was offset to more positive values for NaCl and KCl (i.e., to +16 and +14 mV, respectively). However, the trend was offset for NaNO_3_, as we observed an (unexpectedly) lower ζ-potential than at *r*_aq_ = 1, though, similarly to this observation, Předota et al. (2016)^104^ found for rutile (TiO_2_) that negative surfaces (i.e., {Ti}:{O_2_} < 1) were overcompensated by strong adsorption of (inner-sphere) Na^+^ and (outer-sphere) Sr^2+^ ions in NaCl and SrCl_2_ solutions due to the formation of positively charged fluid layers. The layers in the region between the bulk (carrying the negative charge) and the overcompensating layers carried a negative charge and compensated for the excess adsorption of cations. Similarly, in our case of *r*_aq_ = 100 in NaNO_3_ solution, NO_3_^–^ and SO_4_^2–^ ions may have formed negatively charged fluid layers, explaining the counterintuitive ζ-potential result. Furthermore, the ζ potential increased slightly with time in a similar way as *r*_aq_ = 0.01 and 1 (i.e., from +16 and +14 to +19 and +28 mV, respectively). However, for NaNO_3_, the ζ-potential increased moderately from −5 to +14 mV, having its sign reversed. We observed, similarly to *r*_aq_ = 1, that the initial period for KCl and NaNO_3_ solutions showed a more scattered ζ-potential distribution compared to NaCl (i.e., Figure 3e compared to Figure S17c,i). Therefore, for *r*_aq_ = 1, two populations may have been present. In addition, in the NaNO_3_ solution, the count rate dropped significantly after 2 h (Figure S17i) and, at the same time, the ζ-potential decreased significantly. Causative may have been the sedimentation of particles, resulting in fewer counts (Supporting Information-VIII). Otherwise, the ζ-potential for the KCl and NaNO_3_ solutions developed similarly, while the NaCl solution developed less strongly at *r*_aq_ = 100, which was also observed by the difference in count rates and at which time they reached a maximum (i.e., for KCl and NaNO_3_ during the first hour and for NaCl between 5000 and 8000 s; Figure S17c,i versus S15f).

### 3.10 Implications for (Tailoring) Electrolyte Crystal Nucleation and Growth Mechanisms

Our experiments show that the Ba^2+^ to SO_4_^2–^ ion activity ratio strongly affects the ζ-potential during barite formation, next to pH and, to a lesser extent, the type of background electrolyte. In sulfate-excess conditions, particles initially develop with a negative ζ-potential, and in barium excess conditions with a positive ζ-potential. In the first case, the particles’ ζ-potential subsequently evolves to ∼0 mV within about an hour. In the second case, the particles maintain the positive ζ-potential for the duration of our experiments. This may have implications for the particles’ (aggregation and agglomeration) behavior and the interaction of the particles with any impurity ions and surfactants.

The first moments (i.e., before our first ζ-potential measurement was recorded) of barite crystallization at high supersaturation show strong signs of aggregation.^9^ At intermediate supersaturation, the aggregation of uncharged barite particles will likely be more favorable compared to charged particles as higher charges may induce behavior like charge-stabilized colloids.^105−107^ This has also been observed in DLS and molecular dynamics simulations for CaCO_3_,^108^ where aggregates at {Ca^2+^}:{CO_3_^2–^} = 1 grew faster and larger than at {Ca^2+^}:{CO_3_^2–^} ≠ 1.

Moreover, solution stoichiometry may improve mineral or nanoparticle interactions with their surroundings. For example, since barite surfaces can carry a positive or negative charge solely caused by solution stoichiometry, it can influence the fate of barite in soils, sediments, and other porous media due to differences in interactions between barite particles and grains/pore walls.^109^ For example, free barium ions may be highly mobile in soil as Ba may be associated with soil colloids by ion exchange.^110^ Negatively charged sites on soil colloids could promote barite dissolution over time, thereby acting as cation exchange sites for barite-derived barium. If, however, the barite particles carry a negative charge (i.e., at *r*_aq_ ≪ 1), due to sulfate excess in the Stern layer (Figure 5a), barium is much less likely to associate with soil colloids and, therefore, barium is less mobile than at *r*_aq_ ≫ 1 conditions. In addition, surface charge may help to explain mechanisms in the biological uptake of barite.^111^ For example, bacterial cells adsorb a higher number of barite nanoparticles that have a positive surface charge.^112^ Also, our observed differences in the ζ-potential sign imply that different interactions with proteins can be expected (cf. Patil et al. (2007)^111^). Lastly, influencing the surface charge via solution stoichiometry may help to improve processes that take place in the chemical, paint, and filler industries,^113−116^ among others, by coordinating the stability of suspensions and, ultimately, controlling the time scale and amount of barite deposition.

## 4 Conclusions

Our experimental results show that the {Ba^2+^}:{SO_4_^2–^} (solution stoichiometry) during crystal nucleation and growth of barite has a strong impact on the surface charge of the barite particles formed. At a constant initial degree of supersaturation (Ω_barite_ = 1000), temperature, and ionic strength, barite crystals:

- have a positive ζ-potential when *r*_aq_ ≫ 1 and a negative ζ-potential when *r*_aq_ ≪ 1
- nucleate and grow on a shorter time scale than the development of the EDL
- carry a more negative ζ-potential at alkaline pH
- have a ζ-potential that evolves in the first hour in NaCl BE solutions and faster in KCl and NaNO_3_ solutions (for *I* ∼ 0.02 M) before stabilizing

The ζ-potential and, therefore, the surface charge can be influenced by the solution stoichiometry during crystal formation. Ultimately, solution stoichiometry may be used to predict the fate of barite in environmental settings and to regulate industrial BaSO_4_ (surface chemistry) processes. Therefore, solution stoichiometry is an additional parameter that can influence the stability of suspensions of ionic minerals in general, tailor the surface charge of the particles during formation, and likely affect their reactivity toward surfactants.

## Supporting Information Available

Motivation for chosen physicochemical conditions, Debye length variances during experiments, SEM images of barite particles with varying *r*_aq_, validity of Henry’s function with respect to the product of the inverse Debye length and particle radius, influence of surface conductivity on Henry’s function, influence of particle–particle interactions on Henry’s function, structure factor calculations, effects of aggregation and agglomeration, sedimentation, and crystal structure on ζ-potential, data quality evaluation of performed M3-PALS measurements, autocorrelation functions, diffusion distributions, and particle size intensity distributions associated with the “in-between” size measurements, additional particle size distributions, and additional figures on ζ-potential evolution for different *r*_aq_, pH, and monovalent BE solutions (PDF)
