# Supplementary material for: Impact of Solution {Ba2+}:{SO42–} on Charge Evolution of Forming and Growing Barite (BaSO4) Crystals: A ζ—Potential Measurement Investigation
Source: ACS Omega. 2023 Nov 7;8(46):43521–37. doi: 10.1021/acsomega.3c03727 (PMC10666142; doi:10.1021/acsomega.3c03727)
Supplement: Supplementary file 1 — ao3c03727_si_001.pdf [file ao3c03727_si_001.pdf]

The Impact of Solution $\{Ba^{2+}\}:\{SO_4^{2-}\}$ on Charge Evolution of Forming and Growing Barite ($BaSO_4$) Crystals: ζ – Potential Measurements Investigation

Sergěj Y. M. H. Seepma*,[a] Bonny W. M. Kuipers,[b] Mariette Wolthers[a]

[a] Utrecht University, Department of Earth Sciences, Princetonlaan 8A, 3584 CB Utrecht, The Netherlands

[b] Utrecht University, Van 't Hoff Laboratory for Physical and Colloid Chemistry, Debye Institute for Nanomaterials Science, Padualaan 8, 3584 CH Utrecht, The Netherlands

---

Supporting Information

I --- Appropriate Physicochemical Conditions for ζ-Potential Measurements

The initial Ω_{barite} was chosen carefully with regards to the accuracy of the M3-PALS measurements. Trial-and-error optimization was used to find this initial Ω_{barite} (effectively defining what the expected potential particle concentration would be at equilibrium), which is a trade-off between minimizing aggregation and agglomeration conditions (i.e. a concentration too large) and the signal-to-noise ratio (i.e. a too low concentration). A concentration too large is accompanied with increasing blackening of the electrodes, affecting the field strength and the measurement adversely (i.e. drifting ζ-potential values), and the loss of measurable particles that became too large due to agglomeration and ultimately sank to the bottom of the cell. Clogston & Patri (2011)[1] mentioned that samples should be optically clear and not turbid for M3-PALS. We believe that the used initial Ω_{barite}-value leads to an intermediate particle concentration that provided sufficient signal while minimizing aggregation and agglomeration. The challenge of investigating barite formation is the very low solubility ($K_{sp} = 10^{-9.99}$), which meant we had to work with a high degree of supersaturation to create enough material to obtain an appropriate signal-to-noise ratio.

Automatically, this brings questions to the prerequisite that the particles in solution should only be brought into motion by the applied electric field. Anything else that would affect the motion affects directly the apparent ζ-potential. That means that particle-particle interactions (i.e. agglomeration and long-lasting structuring) should have preferentially been minimized, so that particles could behave as if they were in an infinite medium/solvent. A high degree of supersaturation is therefore not ideal. However, this had to be balanced by the practical limits of the scattered light's detection, which was defined by the system we investigated. Most likely, aggregation took place before the initial ζ-potential measurements in each of our experiments based on our backsacttering detection angle (BSD) particle size observations (i.e. SI-X and SI-XI), but was most likely negligible during the measurements. Also, we estimated by DLVO calculations that agglomeration is negligible (SI-VI). In addition, long-lasting structuring of the particles was negligible (SI-VI).

Lastly, although CO_2 dissolution is supposedly slow in alkaline environments,[2] we could not rule out that small amounts of CO_2 may have been converted into CO_3^{2-} in solutions nos. 3.4 - 3.6 (Table 1), which may

have led to witherite ($BaCO_3$) formation alongside barite. In that case, witherite has a different refractive index than barite (1.504 – 1.529 compared to ~ 1.64)[3] and forms more needle-like (or acicular) structures comparable to that of aragonite ($CaCO_3$) and has different crystallization kinetics than barite.[4] Consequently, this may have led to two different types of crystals with a dissimilar ζ-potential, causing the interference as observed in Figure S16i. This would have been most prominent at r_{aq} = 100, where the relatively large concentration of Ba^{2+} ions increased the ionic activity product (i.e. $\{Ba^{2+}\}\{CO_3^{2-}\}$) to form $BaCO_3$. If the 'noise' was caused by co-existing witherite formation, then the noise disappeared in the second half of the experiment, because witherite was at least more than 2 orders of magnitude less supersaturated than barite. Consequently, barite crystals were present at much larger amounts than witherite toward equilibrium and dominated the ζ-potential signal.

However, our SEM results at pH = 10 (i.e. Figure 1j-l) showed that no witherite crystals were formed and our measured pH-values did not drop notably at those conditions, which would be expected in the case of witherite formation. Based on hydration and hydroxilation rates of CO_2 at pH 10,[2] a maximum of 19% CO_2 saturation was reached after 48 hours in our prepared growth solutions. Chemical speciation calculations show that a 19% CO_2 saturation (i.e. 19% of the current pCO_2 of 400 ppm) leads to a slight supersaturation of witherite only for conditions of pH = 10 and r_{aq} = 100 (i.e. initial $\Omega_{witherite}$ = 4.3), while calculations showed undersaturation with respect to witherite for all other conditions. However, at such low supersaturation, nucleation of witherite is very unlikely and likely outcompeted by barite nucleation and growth.

II --- Evolution of Ionic Strength and Debye Length during Experiments

We have calculated the ionic strength at the initial and equilibrium conditions among the different predefined solutions, to check if that the ionic strength did not change significantly during our experiments. The Debye Length was calculated by the following equation:[5]

$$\kappa^{-1} = \sqrt{\frac{\varepsilon_r \varepsilon_0 k_B T}{2 N_A e^2 I}} \tag{S1}$$

where ε_r is the fluid's dielectric constant [-], ε_0 the permittivity of free space [C^2 s^2 kg^{-1} m^{-3}], k_B the Boltzmann constant [m^2 kg s^{-2} K^{-1}], T the absolute temperature [K], N_A Avogadro's constant [mol^{-1}], e the elementary charge [C] and I the ionic strength [M]. The relationship between the Debye Length over the range 1 mM < I < 1 M is shown in Figure S1a. Figure S1b displays the change in I as $BaSO_4$ formed during batch experiments. Figure S1c shows the resulting change in the Debye Length at the surface of the $BaSO_4$ particles. The Debye length varied from ~2.1 nm to ~2.3 nm (Figure S1c). Therefore, our electrical double layer (EDL) stayed fairly constant throughout the experiment for each condition.

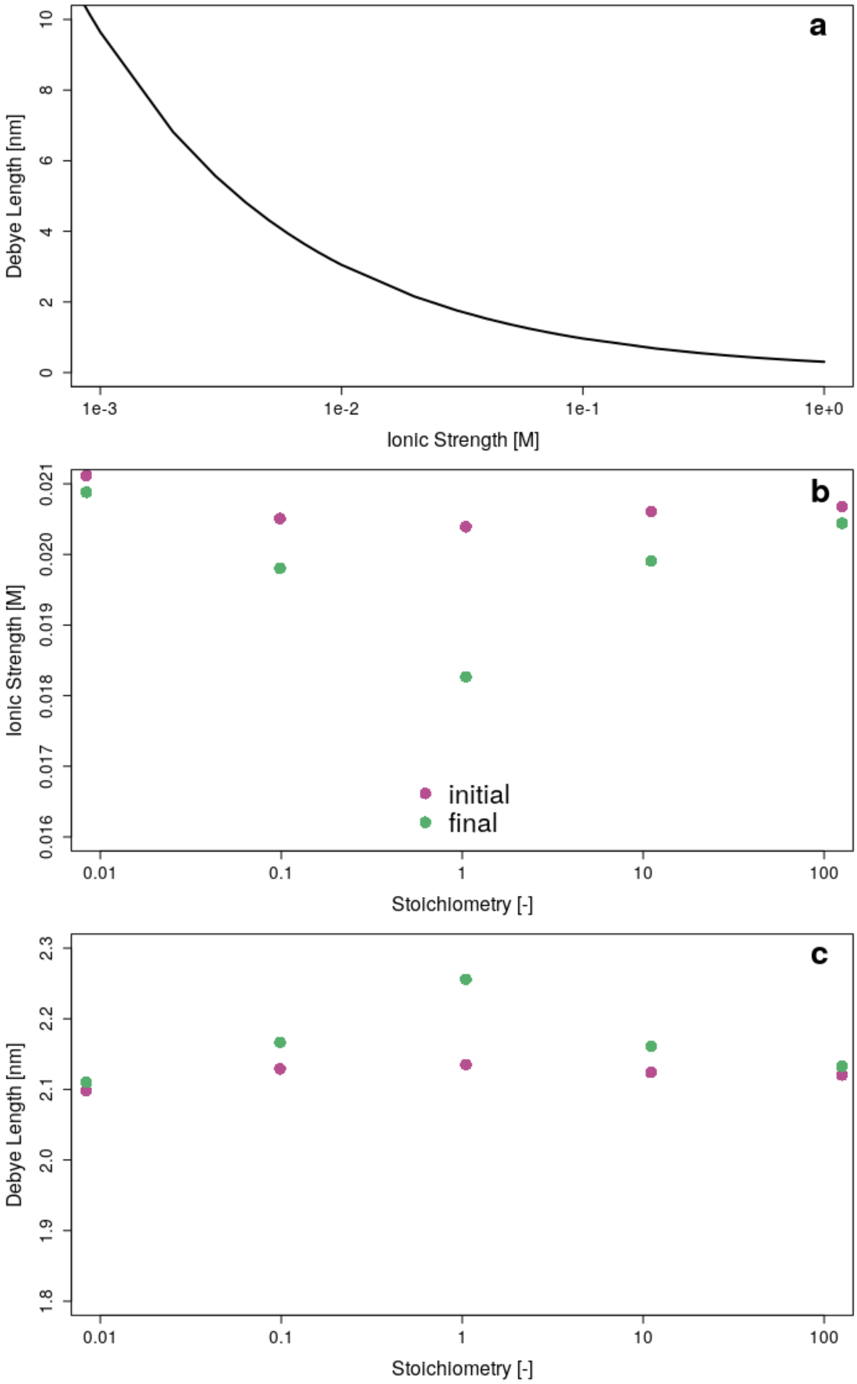

Figure S1: The dependency of the Debye length on the lower region of ionic strength (a), the drop in ionic strength during batch DLS measurements when the system approached equilibrium (b) and the associated change in Debye length (c).

III --- SEM Particle Characterization of Barite for Solution Nos. 2.1-2.5

The morphology of barite particles for NaCl solutions at circumneutral pH (i.e. 5.5 – 7.4) and in NaCl solutions with varying r_{aq}, including r_{aq} = 0.1 and 10, is shown in Figure S2.

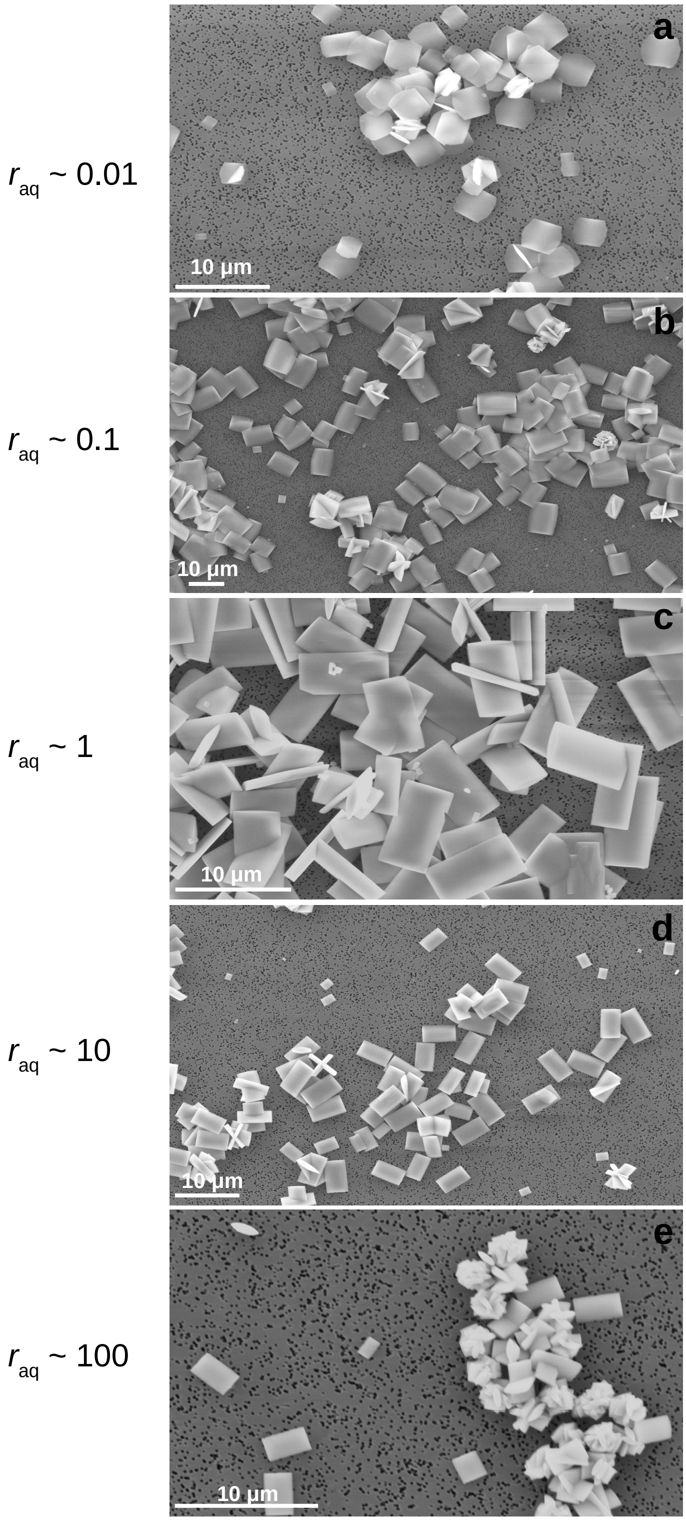

Figure S2: Morphology of the formed barite particles after approximately 3 hours (i.e. near equilibrium). The SEM images correspond to the conditions listed in Table 1; a = (solution no.) 2.1, b = 2.2, c = 2.3, d = 2.4, e = 2.5. The size indicator (white bar) is shown in the bottomleft and is 10 µm for each figure.

IV --- Smoluchowski Limit of Henry's Function

When performing ζ-potential measurements during particle formation, it is important to validate if the Smoluchowski limit (i.e. $f(\kappa\alpha) = 1.50$) of Henry's function (i.e. Equation S2) can be used. Henry's function depends on the geometry of the particle and on the product between the particle size radius and inverse Debye length (κa).[6] Furthermore, Henry's function is restricted in the sense that the (absolute) potential of the surfaces should be approximately less than 25 mV and the distortion of the electrical double layer, by retardation and relaxation, should be minimal during electrophoresis (i.e. strongly determined by I). Though it is expected that retardation of the (charged) particles movement would occur due to the ion-containing fluid surrounding the particles in our chemical solutions, this is corrected for in the Henry function by the measurement of electro-osmosis.[6] Relaxation effects where ions have to constantly rearrange themselves due to the charge disbalance in the ion-containing fluid surrounding the particles of interest were considered negligible, as the Debye length in our investigated solutions is expectedly short ($I = 0.02$ M).

Nonetheless, we investigated how reasonable it is to assume that the Smoluchowski limit is always applicable for our measurements. To calculate at which particle sizes the Smoluchowski limit is valid, the following approximation proposed by Ohshima[7] for the original Henry equation has been used, because the original Henry equation has a hiatus for κa-values between 5 and 25:[8]

$$f(\kappa\alpha) = 1 + \frac{1}{2 * \left(1 + \left(\frac{2.5}{\kappa\alpha * [1 + 2e^{-\kappa\alpha}]}\right)\right)^3} \qquad \text{(S2)}$$

Note that $a = r_p = 0.5d_p$ = particle radius. Equation S2 leads to a deconvolved sigmoidal-like relationship between $f(\kappa a)$ and κa (Figure S3) and has a maximum of 1% relative error. The relationship in Figure S3 is only valid for $| \zeta | < 25$ mV, because relaxation effects become more significant at higher ζ-potential values and affect the value for Henry's function substantially.[9] In our experiments, where I is ~ 0.02 M, κa is ~ 0.07 (i.e. (0.3 nm/2) / 2.2) at $d_p = 0.3$ nm, while κa is ~ 2300 at $d_p = 10000$ nm. Figure S3 shows that we cannot

apply the Smoluchowski limit over the entire d_p range, (note that 4κα ~ d_p (and 2κα ~ r_p), because (d_p / 2)($κ^{-1}$) ~ d_p / 4) and that a different value of *f*(κα) should be considered in the interpretation of our results. However, our measurements in the forward detection angle (FWD) show d_p-values ≥ 200 nm and Figure S3 shows that the Smoluchowski limit of the Henry function is accurate for d_p ≥ 300 nm, for which *f*(κα) ≥ 1.45. Based on Figure S3, all ζ-potential values for particles > ± 100 nm are valid with the use of the Smoluchowski limit with a maximum error of 26.5% for *f*(κα) for particles of 100 nm.

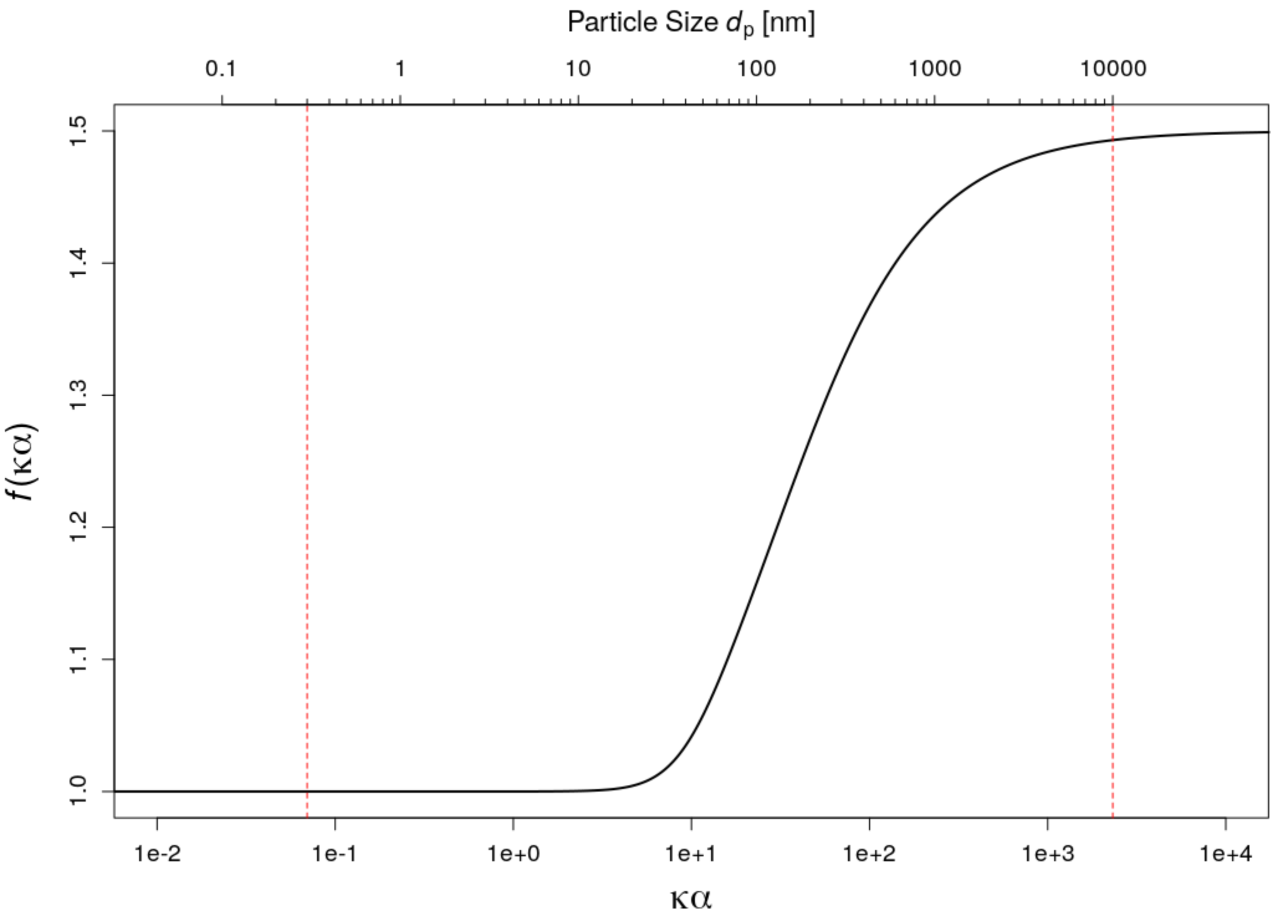

Figure S3: The relationship between Henry's function, which is an approximation proposed by Ohshima, and κa. The particle size diameter d_p is about 4.3 times as large as κ*a*. The DLS particle size range is between the red-dotted lines. The Debye-Hückel limit is accurate for d_p ≤ 11 nm (i.e. *f*(κ*a*) ≤ 1.05) and the Smoluchowski limit for d_p ≥ 300 nm (i.e. *f*(κ*a*) ≥ 1.45). d_p-values that fall in between would require a correction.

Another challenge is the interpretation of the ζ-potential and what that means for the surface charge of the measured particles. The use of the Smoluchowski-limit of the Henry function comes with the assumption that particles behave as ideal hard spheres. The hard-sphere approach for ions is mostly used for inorganic mineral systems, where the anion and cation are represented as hard spheres and assumed to be in contact,

although oversimplified by the fact that the size in the hard-sphere model is assumed to be constant with a defined charge and co-ordination number and considers that the attractive forces between anion and cation with identical opposite charge is constant.[10] Lang & Smith (2010)[10] showed that in a soft-sphere model, where some overlap exists between the anion and cation, internuclear distances for Ba^{2+} with most other monatomic anions match better with experimental values compared to the hard-sphere model. However, the choice for the hard-sphere model is still preferred as a more reliable estimate of the surface charge density can be made, especially when the heterogeneity is large with regards to the particle size (polydispersity) and differences in morphology of the particles among different experiments occur.[11]

V --- Influence of Surface Conductivity on the Smoluchowski Limit of Henry's Function

Surface conductivity is also neglected with Henry's function. For $I > 0.1$ M, surface conductivity is usually negligible compared to the bulk water conductivity[9,12,13] and, therefore, the Dukhin number *Du*, which relates surface to bulk dispersant conductivity, is ~ 0. In this case, the measured or apparent ζ-potential is roughly equal to the ζ-potential corrected for surface conductivity. However, when *Du* >> 0.1, surface conductivity may play an important role and nonlinear electrokinetic phenomena need to be considered.[14,15] Therefore, we calculated *Du* with a set of fixed ζ-potential values, Debye length, ionic strength, the translational diffusion coefficient and particle size (Equation S3). *Du* is defined as[16,17]:

$$Du = \frac{\kappa^{\sigma}}{C_{\mathrm{f}}\,\alpha} \tag{S3}$$

where κ^{σ} is the surface conductivity and C_{f} the bulk fluid electrical conductivity. The surface conductivity κ^{σ} is defined as[18]:

$$\kappa^{\sigma} = \frac{4F^2 C_{\mathrm{i}} z^2 D_{\mathrm{Trans}}\left(1 + 3m/z^2\right)}{\kappa RT} \cosh\left(\frac{zF\zeta}{2RT} - 1\right) \tag{S4}$$

where z is the ion valency, C_{i} the ionic concentration of the bulk fluid [M] and m the dimensionless electro-osmosis contribution to the ion motion in the double layer[14] and is defined as:

$$m = \frac{2\varepsilon_0 \varepsilon_{\mathrm{r}} R^2 T^2}{3\mu F^2 D_{\mathrm{Trans}}} \tag{S5}$$

Note that the ion size is important for κ^{σ}, as it determines the magnitude of D_{Trans} and that the ionic strength influences the value of κ. Equation S3 - S5 assumes that the electrolyte is symmetrical, where the respective diffusion coefficients are equal. This is not entirely true (i.e. 1.693×10^{-9} and 2.130×10^{-9} $m^2\ s^{-1}$ for Ba^{2+} and SO_4^{2-} respectively, according to Kadhim & Gamaj (2020),[19] but so far gives the best approximation. For a set

of ζ-potential values (i.e. ζ = 5, 15, 25, 50, 75 and 100 mV), we determined *D*u with the particle size range matching that of the Zetasizer ULTRA (Figure S4a). Figure S4b shows a more detailed image of the range in which surface conductivity plays a significant role. Also, Figure S5 displays the change in *D*u with ζ-potential for a set of fixed values for the particles size in which surface conductivity may be important.

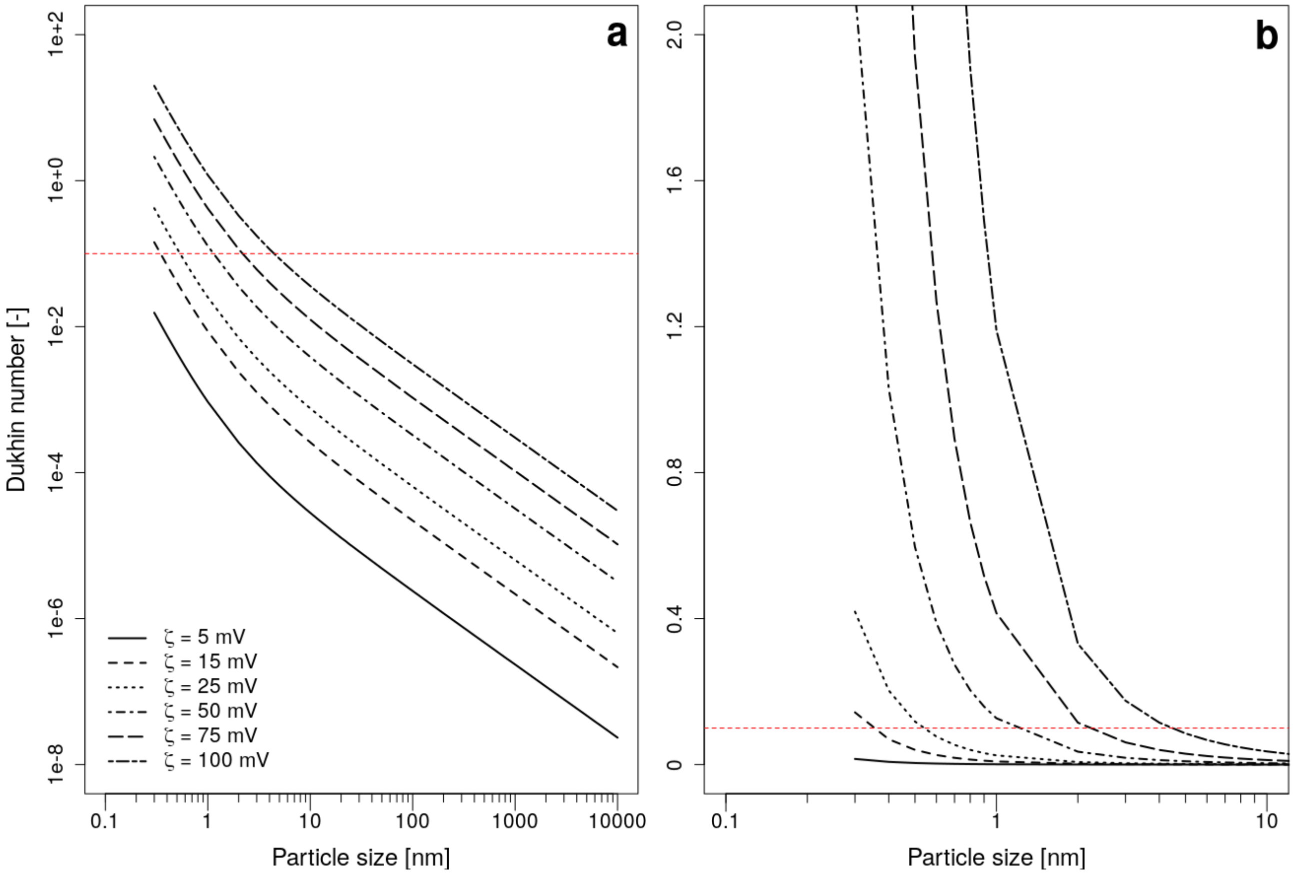

Figure S4: The relationship between the Dukhin number and the particle size at specific ζ-potential values (i.e. 5, 15, 25, 50, 75 and 100 mV). The red-dotted line represents the condition of *Du* = 0.1 and specifies more or less the threshold at which surface conductivity should not be ignored.

Overall, when the particle size is 2 nm or larger and the | ζ | ≤ 50, surface conductivity can be considered as a negligible influence on the measured ζ-potential. However, if these conditions are not met, then the true (corrected) ζ-potential may be significantly larger than the apparent (measured) ζ-potential as the following relationship should be taken into account, assuming that the macroscopic porosity in our synthesized $BaSO_4$ crystals is unity[13,20,21]:

$$\frac{\zeta_a}{\zeta} = \frac{1}{1 + Du} \tag{S6}$$

The ζ-potential measurements in the FWD rarely go beyond ± 50 mV. Therefore, the ζ-potential correction for possible surface conductivity was not needed (Equation S6).

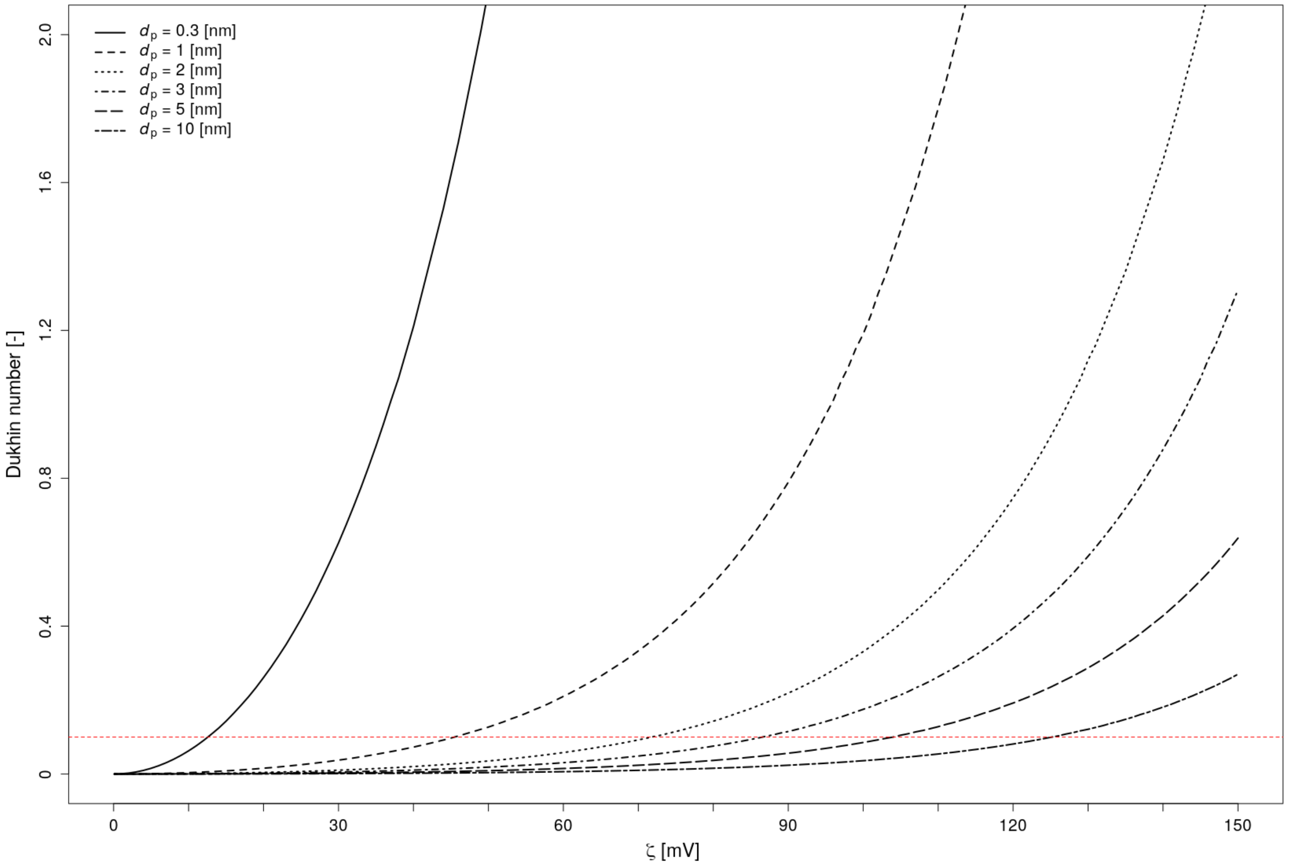

Figure S5: Illustrates the relationship between the Dukhin number and the ζ-potential at specific particle sizes (i.e. d_p = 0.3, 1, 2, 3, 5 and 10 nm), where surface conductivity can be an important factor. The red-dotted line represents the condition of *Du* = 0.1 and specifies more or less the threshold at which surface conductivity should not be ignored.

In addition, we ignored in Equation S4 the possibility of ions in the stagnant layer contributing to the conductivity (i.e. ions in the stagnant layer being responsive due to the electric field; see Jiménez et al. (2005)[22]), because in our experiments, the charge (ζ-potential) is relatively low and the diffusion of ions adsorbed at the stagnant layer is much lower than the diffusion in the mobile layer. According to Delgado et al. (2007)[14] this is a valid assumption and Jiménez et al. (2005)[22] showed that this assumption is valid if the

Dukhin number is lower than 0.05 (see also Figure 2b), which was the case for our rapidly developing systems.

VI --- Influence of Collision Particle-Particle Interactions based on DLVO-Calculations

Besides the importance of κa (SI-IV) and the surface conductivity (SI-V), Henry's function also ignores the Van der Waals interactions and electrostatic repulsion (relaxation effects). To include the relaxation effects, one could use the Modified Booth Equation, which evolved from the Derjaguin-Landau-Verwey-Overbeek (DLVO) theory,[23] and solves the ζ-potential numerically for any set of r_p, ion valences, concentrations and limiting ion conductivities.[9] The numerical solution uses 4 coupled equations that include the Henry function plus 3 relaxation correction functions. By using a 3rd-order polynomial approach to describe these functions, a graph similar to Figure S3 is obtained with a regression coefficient greater than 0.997.[6,9] Another way to obtain information about the importance of the relaxation effects in our systems is to use the extended-DLVO theory, which originates from the 90s[24-26] and describes the stability of suspensions via several established explicit analytical equations. Although, given the results of Deshiikan & Papadopoulos (1998),[6] this effect should not be significant in our case as our | ζ | is rarely is beyond 25 to 30 mV, for completeness we have investigated the relaxation effects with the use of the extended-DLVO theory.

According to the extended-DLVO theory, the key parameter is the total energy of interaction between two solid phases separated by a liquid medium (ΔG_{SLS}). ΔG_{SLS} is the sum of electrostatic forces ($\Delta G_{SLS,EL}$), van der Waals interaction ($\Delta G_{SLS,vdW}$) and hydration forces originating from the solvation of the solid ($\Delta G_{SLS,AB}$). The latter can be understood as electron-acceptor/electron-donor polar interaction contributions due to hydrophilicity or hydrophobicity. Experimental studies have shown that this factor may be significant and should be taken into consideration.[27-29] This implies the following equation:

$$\Delta G_{\mathrm{SLS}} = \Delta G_{\mathrm{SLS,EL}} + \Delta G_{\mathrm{SLS,vdW}} + \Delta G_{\mathrm{SLS,AB}} \quad \text{(S7)}$$

If the ζ-potential has the same value between two distinct particles of the same size, then $\Delta G_{SLS,\,EL}$ can be related to ζ-potential according to the following equation[30-32]:

$$\Delta G_{\mathrm{SLS,\,EL}} = \pi \varepsilon_{\mathrm{r}} \varepsilon_0 r_{\mathrm{p}} \zeta^2 \left[p_{\mathrm{i}} + q_{\mathrm{j}} \right] \quad \text{(S8)}$$

where

$$p_{\mathrm{i}} = \ln\left[\frac{1 + \exp(-\kappa H)}{1 - \exp(-\kappa H)}\right],$$

$$q_{\mathrm{j}} = \ln\left[1 - \exp(-2\kappa H)\right],$$

with κ as the inverse of the Debye Length (Equation S1) and where *H* is the distance between the surfaces of two particles.

$\Delta G_{\mathrm{SLS,\,vdW}}$ and $\Delta G_{\mathrm{SLS,\,AB}}$ require the surface tension from both the medium (ultra-pure water; UPW) and the solid ($BaSO_4$) to be known, γ_L and γ_S, respectively. The surface tension can be split into two components according to van Oss (1994)[25]; γ_{vdW} and γ_{AB}, where the latter consists of the electron-donor (γ^-) and electron-acceptor (γ^+). This leads to the following equation:

$$\gamma = \gamma_{\mathrm{vdW}} + \gamma_{\mathrm{AB}} = \gamma_{\mathrm{vdW}} + 2\sqrt{\gamma^{+}\gamma^{-}} \qquad \text{(S9)}$$

For UPW, the values for $\gamma_{L,vdW}$, γ_L^- and γ_L^+ at room temperature are 21.8, 25.5 and 25.5 mJ m^{-2}, respectively,[33,34] although some alternative values have been proposed.[35] Therefore, γ_L equates to 72.8 mJ m^{-2} according to Equation S9. Gallardo et al. (2000)[33] determined with the thin layer wicking technique[36-38] the values for $\gamma_{S,vdW}$, γ_S^- and γ_S^+ for a 10^{-2} M NaCl solution (e.g. comparable *I* as our investigated systems), which are 51.3 ± 2.0, 63.4 ± 1.9 and 0.0 ± 1.0 mJ m^{-2}, respectively. Consequently, γ_S is equal to 51.3 ± 2.0 mJ m^{-2}.

Now all the parameter values are known to calculate $\Delta G_{\mathrm{SLS,\,vdW}}$ according to the following equation[39,40]:

$$\Delta G_{\mathrm{SLS,\,vdW}} = -\frac{A r_{\mathrm{p}}}{12H}\, f(P_0) \qquad \text{(S10)}$$

where

$$P_0 = \frac{2\pi H}{\lambda_c} \tag{S11}$$

for $P_0 < 0.5$,

$$f(P_0) = \frac{1}{1 + 1.77 P_0} \tag{S12}$$

and for $0.5 < P_0 < \infty$,

$$f(P_0) = -\frac{2.45}{60 P_0} + \frac{2.17}{180 P_0^2} - \frac{0.59}{420 P_0^3} \tag{S13}$$

where λ_c in Equation S11 is the wavelength of intrinsic oscillations of atoms and typically has a value of 10^{-7} m.[41] A in Equation S10 is the so-called Hamaker constant and is defined as[33,42,43]:

$$A = 24\pi H_0^2 \left(\sqrt{\gamma_{S,vdW}} - \sqrt{\gamma_{L,vdW}}\right)^2 \tag{S14}$$

where H_0 is the minimum equilibrium contact distance between the $BaSO_4$ particles, for which Gallardo et al. (2000)[33] used a value of 1.58 Å and van Oss et al. (1990)[44] a value of 1.63 Å. If we use H_0 = 1.58 Å, then we obtain $A = 1.17 \times 10^{-20}$ kg m^2 s^{-2}. Such a value, between 10^{-20} and 10^{-19} kg m^2 s^{-2} (J) is expected for single material in solid-liquid systems.[42,45]

$\Delta G_{SLS,AB}$ is calculated by the following equation[46,47]:

$$\Delta G_{\mathrm{SLS,AB}} = \Xi_{\mathrm{AB}} \pi r_{\mathrm{p}} h_0 \exp\left(\frac{H_0 - H}{h_0}\right) \qquad \text{(S15)}$$

where h_0 is the correlation length of water molecules and varies between ~ 0.2 nm (for nonhydrogen bonded water molecules)[48] and 13 nm.[49] A reasonable value is 1 nm.[25,33,50,51] Ξ_{AB} in Equation S15 contains the acid/base components of the surface free energy of both the $BaSO_4$ and UPW phases and is defined as:

$$\Xi_{\mathrm{AB}} = -4\left(\sqrt{\gamma_{\mathrm{S}}^{+}\gamma_{\mathrm{S}}^{-}} + \sqrt{\gamma_{\mathrm{L}}^{+}\gamma_{\mathrm{L}}^{-}} - \sqrt{\gamma_{\mathrm{S}}^{+}\gamma_{\mathrm{L}}^{-}} - \sqrt{\gamma_{\mathrm{S}}^{-}\gamma_{\mathrm{L}}^{+}}\right) \qquad \text{(S16)}$$

From the experimental values found by Gallardo et al. (2000)[33] (see their page 13), the interfacial energy (γ) between the UPW phase and $BaSO_4$ phase is calculated by using Good's Equation[52]:

$$\gamma = \gamma_{\mathrm{L}} + \gamma_{\mathrm{S}} - 2\left[\sqrt{\gamma_{\mathrm{L}}^{\mathrm{vdW}}\gamma_{\mathrm{S}}^{\mathrm{vdW}}} + \sqrt{\gamma_{\mathrm{L}}^{+}\gamma_{\mathrm{S}}^{-}} + \sqrt{\gamma_{\mathrm{L}}^{-}\gamma_{\mathrm{S}}^{+}}\right] \qquad \text{(S17)}$$

If we insert the values for $\gamma_{L,vdW}$, γ_L^-, γ_L^+ ,$\gamma_{S,vdW}$, γ_S^- and γ_S^+, then we obtain a value of – 22.6 mJ m^{-2} for γ. A value with that order of magnitude is quite common among oxides or ionic crystals, like $BaSO_4$, immersed in water that only have electron donor properties.[53,54] Consequently, it is easier for water to penetrate between those surfaces and hence, lead to repulsion between $BaSO_4$ particles. This would suggest that the interaction energy ΔG_{SLS} (Equation S4.1) should be rather low. Vijayabaskar & Vishveshwara (2010)[55] regarded values of 0 – 5 kJ/mol for ΔG_{SLS} as low energy interactions and ≤ - 20 kJ/mol as high energy interactions. All in all, the consideration of low and high interaction energies by Vijayabaskar & Vishveshwara (2010)[55] and all the parameter values previously addressed, we are now able to solve Equation S7:

$$\Delta G_{\mathrm{SLS}} = \left\{ \pi \varepsilon_{\mathrm{r}} \varepsilon_0 r_{\mathrm{p}} \zeta^2 \left[\ln\left[\frac{1 + \exp(-\kappa H)}{1 - \exp(-\kappa H)} \right] + \ln\left[1 - \exp(-2\kappa H)\right] \right] \right\} - \left\{ \frac{\pi H_0^2 \gamma_{\mathrm{S,vdW}} r_{\mathrm{p}}}{H} f(P_0) \right\} +$$

$$\left\{ -4\left(\sqrt{\gamma_{\mathrm{S}}^{+}\gamma_{\mathrm{S}}^{-}} + \sqrt{\gamma_{\mathrm{L}}^{+}\gamma_{\mathrm{L}}^{-}} - \sqrt{\gamma_{\mathrm{S}}^{+}\gamma_{\mathrm{L}}^{-}} - \sqrt{\gamma_{\mathrm{S}}^{-}\gamma_{\mathrm{L}}^{+}} \right) \pi r_{\mathrm{p}} h_0 \exp\left(\frac{H_0 - H}{h_0} \right) \right\} \quad \text{(S18)}$$

where ε_r, ε_0, κ, H_0, $\gamma_{S,vdW}$, γ_L^+, γ_L^-, γ_S^+, γ_S^- and h_0 are (assumed to be) constants, $f(P_0)$ is a semi-continuous function, ζ and r_p are controlled variables, H the independent variable and ΔG_{SLS} the dependent variable. Figure S6a displays the contributions of $\Delta G_{SLS,EL}$, $\Delta G_{SLS,vdW}$ and $\Delta G_{SLS,AB}$ to ΔG_{SLS}. while Figure S6b and Figure S6c illustrate the relationship between ΔG_{SLS} and H for various r_p and ζ-potential, respectively.

It is difficult to predict the average H in our suspensions, but existing literature in the field of electrokinetics describing definitions for dilute, semi-dilute and concentrated systems gives us an indication. Suspensions having a solid volume percentage of 10% or larger are often categorized in the concentrated regime (Posner (2009)[57] and references therein). Although some categorize suspensions containing a solid volume percentage of 1% or less in the dilute regime,[56] many consider this to be true only at a solid volume percentage of 0.1% or less.[57-59] Between solid volume percentages of 0.1% and 10%, the semi-dilute regime persists. Our investigated suspensions at initial Ω_{barite} = 1000, show a maximum solid volume percentage of ~ 0.013% at equilibrium, meaning that all investigated suspensions fall into the dilute regime. Whereas in concentrated and semi-dilute regimes the distances between particles are much smaller than the particle size and approximately in the range of the particle size, respectively, the distances between particles in the dilute regime is much larger than the particle size.[56,60,61]

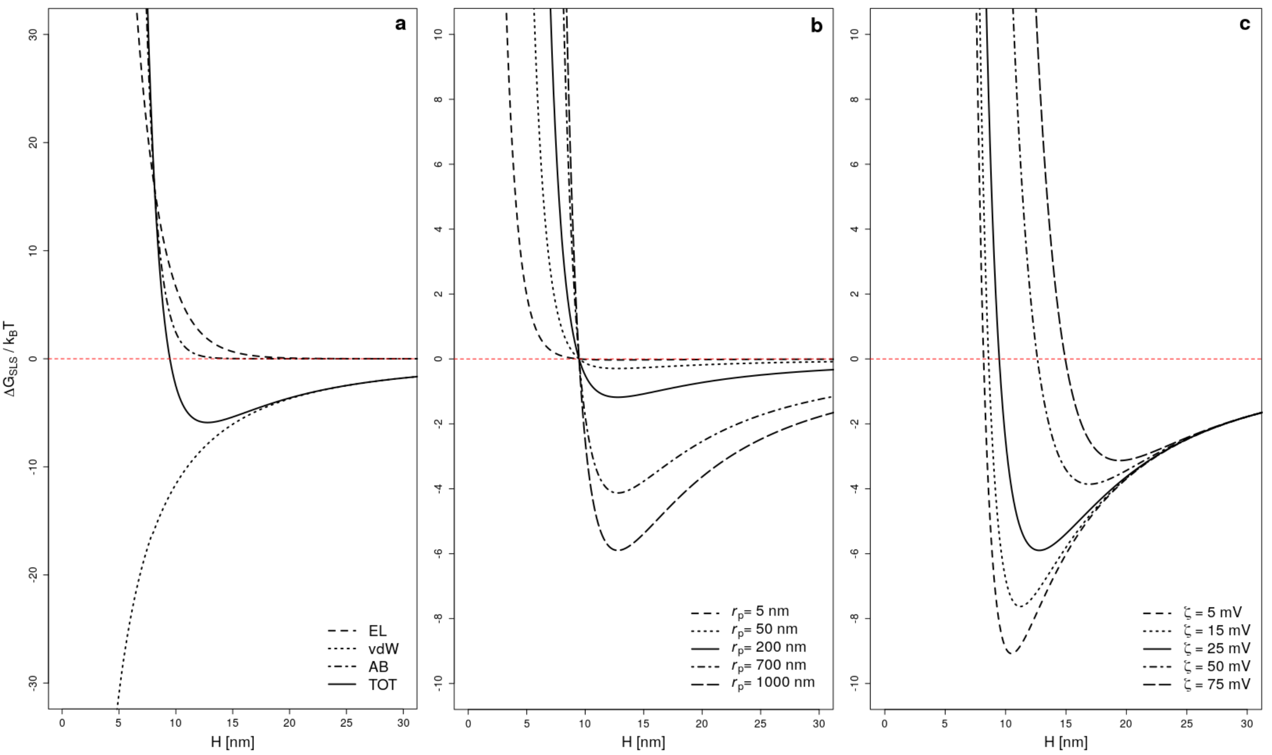

Figure S6: Contributions of the electrostatics (EL), van der Waals (vdW) and hydration (AB) to the total surface free energy (TOT) with *H* (the distance between particles) for r_p = 1000 and ζ = 25 mV (a), the influence of r_p on the total surface free energy with *H* for ζ = 25 mV (b) and the effect of ζ-potential on the total surface free energy with *H* for r_p = 1000 nm (c).

According to Figure S6, the total surface free energy is negligibly negative for $H > 30$ nm, independent of ζ-potential and r_p. A simple equation for monodisperse spheres has been derived to estimate the average separation distance between two particles in suspension[62,63]:

$$H = 2r_p\left[\sqrt{\left(\frac{1}{3\pi\phi_s} + \frac{5}{6}\right)} - 1\right] \qquad \text{(S19)}$$

where $ø_s$ is the solid volume percentage. Using a value for r_p of 100 nm (smallest observed particle size during batch experiments in FWD) and 0.013 for $ø_s$ (the largest equilibrium solid volume fraction we used), then we estimate that *H* is approximately 400 nm (i.e. 413 nm). Larger r_p would result in larger *H*.

Huang et al. (2010)[64] showed, with the use of the extended-DLVO theory, that differences in crystal morphology may generally affect the particle-particle interactions more than the differences in surface roughness. However, in our case, that would not compensate enough such that the interparticle distance would be < 30 nm for particle sizes > 200 nm and would still lead to the conclusion that the Smoluchowski limit of the Henry function was evidently valid for our batch ζ-potential experiments.

In summary, irrespective of high and low interaction energies, we considered the relaxation effects for our barite suspensions negligible as the calculated average separation distance is far from the position of the well (Figure S6).

VII --- Influence of Long-Lasting Particle-Particle Interactions

Besides the likelihood of agglomeration in our systems (SI-VI), we also calculated if the position of particles in solution are influenced by their neighboring particles (i.e. long-lasting particle-particle interactions due to packing) and determined therefore the structure factor. If the structure factor is not equal or close to unity, particles are not randomly positioned in the solution. Ultimately, this has an effect on the scattering signal during light scattering measurements.[65] The absolute total scattering intensity I_{tot} is defined as:

$$I_{\text{tot}} = I_0 K_c N V_p^2 (\text{'contrast factor'})^2 P(q) S(q) \quad \text{(S20)}$$

where I_0 is the incident beam intensity, K_c a constant and a factor related to the measurement set-up (i.e. it includes wavelength and scattering volume-detector distance among others) [m^{-6}], N is the total number of particles, V_p the particle volume, $P(q)$ the form factor of a single particle and $S(q)$ the inter-particle structure factor. The 'contrast factor' is the difference of a specific physicochemical property between the solid and liquid (e.g. the difference in density ($\Delta\rho$) between the solid ρ_s and the liquid ρ_l or the difference in refractive index ($\Delta\eta$) between the solid η_s and the liquid η_l). Note that when $P(q\rightarrow0) = 1$, $P(q\rightarrow\infty) = 0$ and $S(q\rightarrow\infty) = 1$.[66] In Equation S20 (and the following ones), q is the wave vector and is defined as:

$$q = \frac{4\pi\eta_l}{\lambda_0} \sin\left(\frac{\theta}{2}\right) \quad \text{(S21)}$$

where η_l is the refractive index of the medium, λ_0 the wavelength of the light in the medium and θ the angle of light diffraction.

The shape factor $P(q)$ is defined as (i.e. in the Porod-regime):

$$P(q) = \left[\left(\frac{3}{qr_{\mathrm{p}}} \right) \left(\frac{\sin(qr_{\mathrm{p}})}{(qr_{\mathrm{p}})^2} - \frac{\cos(qr_{\mathrm{p}})}{(qr_{\mathrm{p}})} \right) \right]^2 \qquad \text{(S22)}$$

where r_{p} is the particle radius. When $P(q \rightarrow 0) = 1$ and the gyration radius is used (i.e. $R_{\mathrm{g}}^2 = 3r_{\mathrm{p}}^2/5$), then the Guinier approximation applies:

$$P(q) = 1 - \frac{(qR_{\mathrm{g}})^2}{3} + \frac{(qR_{\mathrm{g}})^4}{21} \qquad \text{for} \quad (qR_{\mathrm{g}})^2 < 1 \qquad \text{(S23)}$$

The Ornstein-Zernike integral equation[67] is used to describe the inter-particle interactions and is defined as:

$$c(r_{\mathrm{id}}) = g(r_{\mathrm{id}}) \left[1 - e^{-\frac{w(r_{\mathrm{id}})}{k_{\mathrm{B}} T}} \right] \qquad \text{(S24)}$$

where $c(r_{\mathrm{id}})$ represents direct interactions among two particles and $g(r_{\mathrm{id}})$ indirect interactions by other particles in the surrounding. The problem with Equation S24 is that $c(r_{\mathrm{id}})$ and $g(r_{\mathrm{id}})$ are both unknowns and represent distances and therefore cannot be negative. For that reason, a so-called 'closure'-relationship is needed to the Ornstein-Zernike integral equation. The simplest closure relationship, which is able to describe a colloidal system well[68] and which provides an analytical solution,[69] is that of Percus-Yevick[70]:

$$w(r_{\mathrm{id}}) = 0 \qquad \text{for} \quad r_{\mathrm{id}} > d_{\mathrm{p}} \qquad \text{(S25.a)}$$

$$w(r_{\mathrm{id}}) = \infty \qquad \text{for} \quad r_{\mathrm{id}} < d_{\mathrm{p}} \qquad \text{(S25.b)}$$

where d_{p} is the particle diameter. Then the analytical solutions for $c(r_{\mathrm{id}})$ are the following:

$$c(r_{\mathrm{id}}) = 0 \qquad \text{for} \quad r_{\mathrm{id}} > d_{\mathrm{p}} \qquad \text{(S26.a)}$$

$$c(r_{\mathrm{id}}) = -\lambda_1 - 6\Phi_{\mathrm{s}}\lambda_2\frac{r_{\mathrm{id}}}{d_{\mathrm{p}}} - \frac{\Phi_{\mathrm{s}}}{2}\lambda_1\frac{r_{\mathrm{id}}^3}{d_{\mathrm{p}}^3} \quad \text{for} \quad r_{\mathrm{id}} < d_{\mathrm{p}} \tag{S26.b}$$

where Φ_s is the solid/particle volume fraction [-] and the dimensionless λ_1 and λ_2 parameter are defined as:

$$\lambda_1 = \frac{(1 + 2\phi_{\mathrm{s}})^2}{(1 - \phi_{\mathrm{s}})^4} \tag{S27}$$

$$\lambda_2 = \frac{-(1 + \phi_{\mathrm{s}}/2)^2}{(1 - \phi_{\mathrm{s}})^4}$$

The analytically derived Fourier transform of the direct correlation function (Equation S26.b) is the following:

$$\bar{N}C(q) = -24\Phi_{\mathrm{s}}\lambda_1\left[\frac{\sin(qd_{\mathrm{p}})-(qd_{\mathrm{p}})\cos(qd_{\mathrm{p}})}{(qd_{\mathrm{p}})^3}\right] - 6\Phi_{\mathrm{s}}\lambda_2\left[\frac{(qd_{\mathrm{p}})^2\cos(qd_{\mathrm{p}})-2(qd_{\mathrm{p}})\sin(qd_{\mathrm{p}})-2\cos(qd_{\mathrm{p}})+2}{(qd_{\mathrm{p}})^4}\right]$$
$$-\Phi_{\mathrm{s}}\frac{\lambda_1}{2}\left[\frac{(qd_{\mathrm{p}})^4\cos(qd_{\mathrm{p}}) - 4(qd_{\mathrm{p}})^3\sin(qd_{\mathrm{p}}) - 12(qd_{\mathrm{p}})^2\cos(qd_{\mathrm{p}}) + 24(qd_{\mathrm{p}})\sin(qd_{\mathrm{p}}) + 24\cos(qd_{\mathrm{p}}) - 24}{(qd_{\mathrm{p}})^6}\right] \tag{S28}$$

where $\bar{N}$ is the number density of particles and C a material constant, which is defined by the density and refractive index among others,[66] but is not important to know for the solution of the structure factor. The structure factor for perfect hard spheres is defined as:

$$S(q) = \frac{1}{1 - \bar{N}C(q)} \tag{S29}$$

Using Equations S21, S22, S27-S29, Figure S7a-d was obtained, where the structure ($S(q)$) and shape factor ($P(q)$) were plotted against qr_{p} for $BaSO_4$ particles in aqueous suspensions at λ_0 = 632.8 nm. Therefore,

Figure S7a-d is independent of particle size. Figure S7a and S7b show the trend on a linear scale, while Figure S7c and S7d show the same trend on a logarithmic scale. Φ_s = 0.00013 corresponds roughly with conditions of initial Ω_{barite} = 1000 and r_{aq} = 1 and it shows that the structure factor does not take any role (i.e. $S(q) = 1$), except at $qr_p \sim 0$ (Figure S7b and S7d). Contrary, at Φ_s = 0.13, meaning that there is 1000x more solid material present in an equal volume than can be formed in our experiments, it shows that for $qr_p < 8$, $S(q) \neq 1$. In Figure S7a and S7b, it seems that $P(q)S(q) = 0$ at larger qr_p, but on a logarithmic scale one can observe that that is not the case.

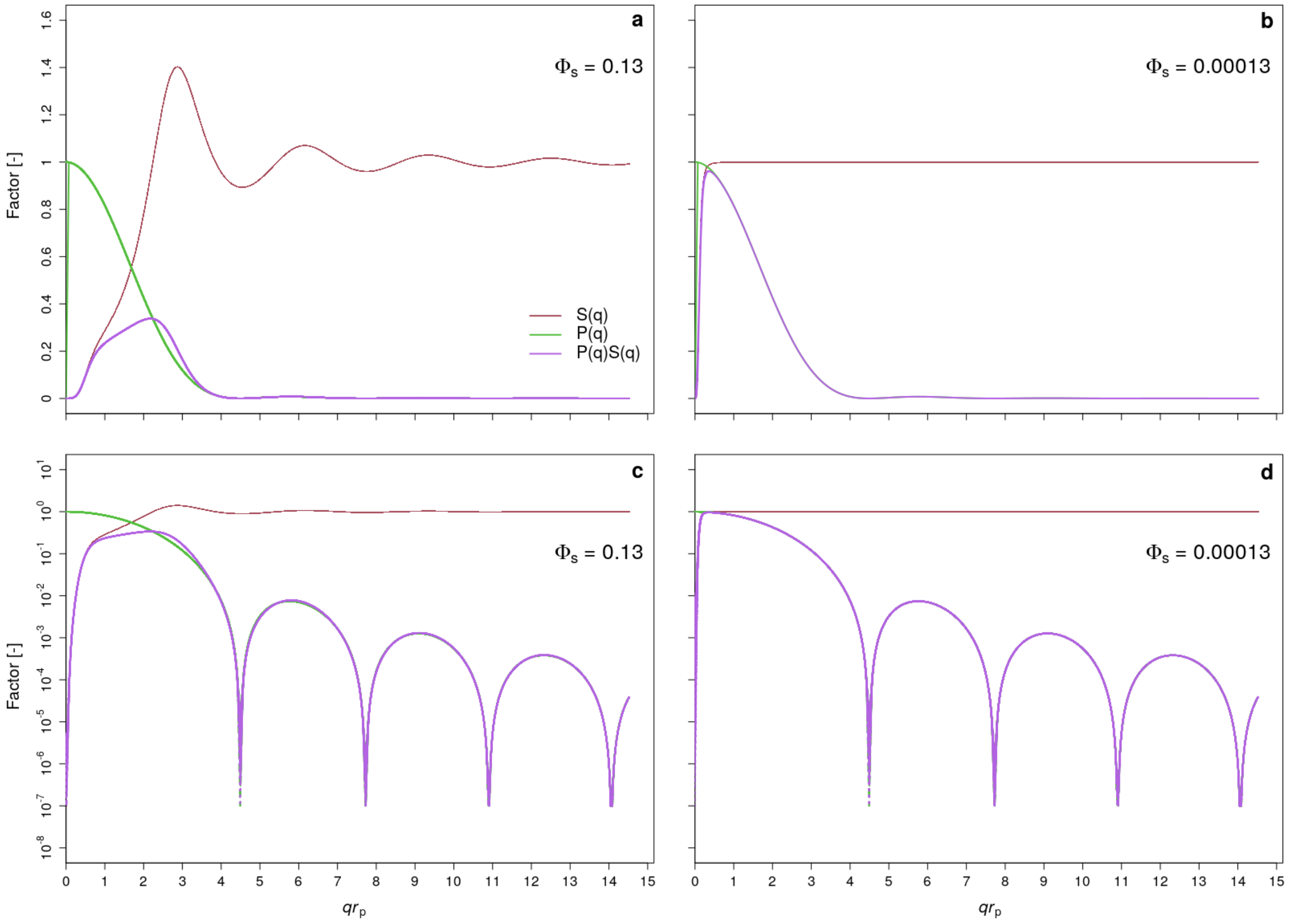

Figure S7: Shape factor $P(q)$, structure factor $S(q)$ and the product of both (i.e. $P(q)S(q)$) as a function of qr_p for Φ_s = 0.13 and Φ_s = 0.00013 on both a linear and a logarithm scale; linear scale for Φ_s = 0.13 (a), linear scale for Φ_s = 0.00013 (b), logarithmic scale for Φ_s = 0.13 (c) and logarithmic scale for Φ_s = 0.00013 (d). Note: There are tiny 'gaps' in the calculated data. Causative is q, because of its sinusoidal nature; With increasing θ, from 0 to 360, q continuously increases and decreases, causing the data to be non-ordered with respect to qr_p. The calculated data was obtained by steps of θ = 0.01 (i.e. 36,001 data points were acquired).

To understand better what Figure S7 entails with regards to light scattering measurements, one can plot $P(q)$ and $S(q)$ as a function of θ in polar coordinates (Figure S8).

At Φ_s = 0.00013, the structure factor is at almost every angle equal to unity (Figure S8a). The ‘gaps’ at certain θ-values, where $S(q) < 0.99$ are about 0.17. In total, this equates to a total angle of about 9.6, where $S(q) < 0.99$. At this solid volume fraction, $S(q)$ = 0.99959 at the forward scattering detection angle (i.e. θ = 12.78°), $S(q)$ = 1.00001 at the side scattering detection angle (i.e. θ = 90.00°) and $S(q)$ = 0.99999 at the back scattering detection angle (i.e. θ = 174.70°). Conversely, at Φ_s = 0.13 (i.e. 1000x more solid volume than can be formed in our systems), the structure factor is never reaching a constant period where $S(q)$ is between 0.99-1.01, which would mean that structuring of particles is a significant factor (Figure S8b). To conclude, it is safe to assume particle structuring did not occur in our systems/was not observable at the angles we measured.

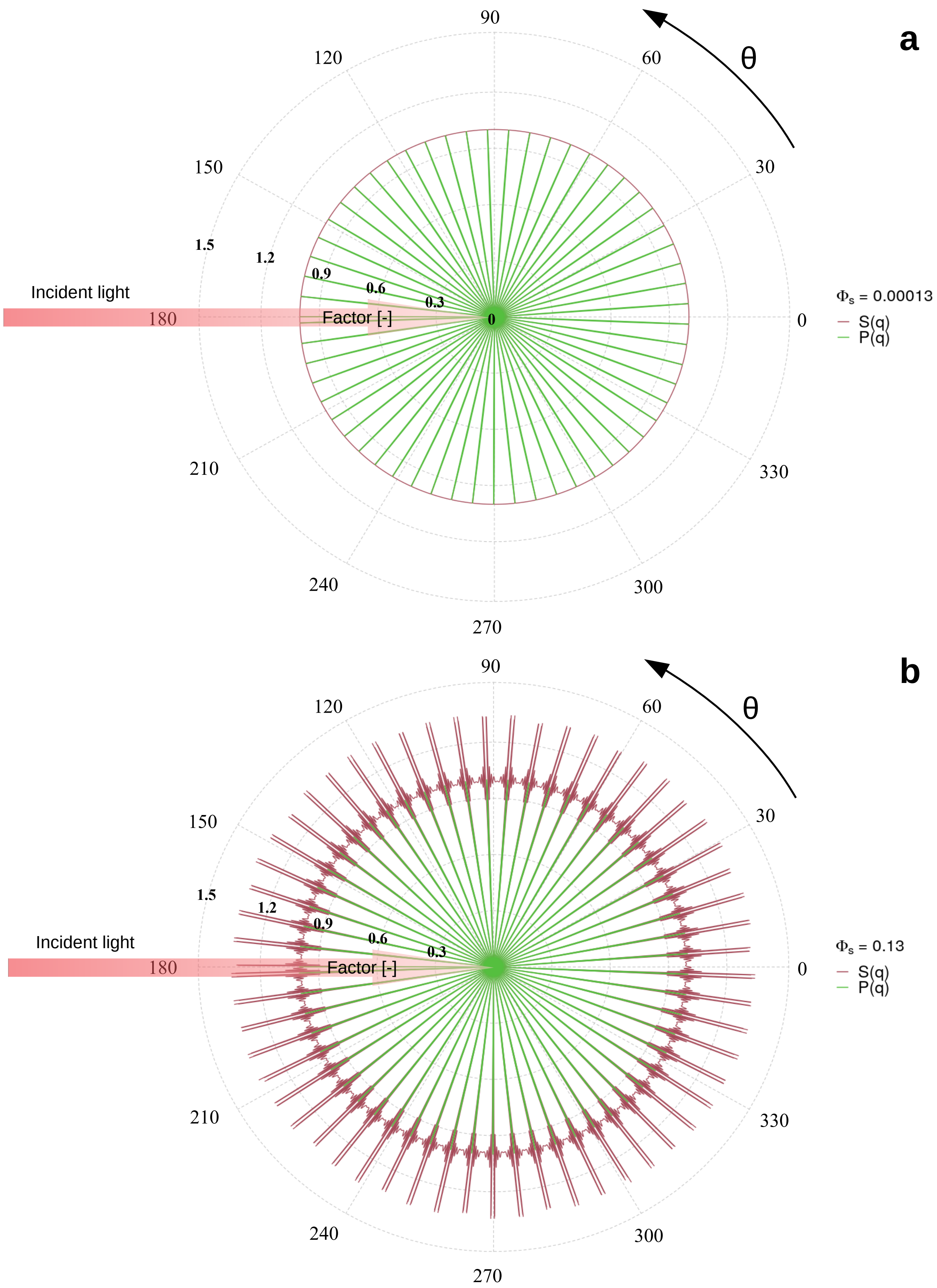

Figure S8: Shape factor $P(q)$ and structure factor $S(q)$ versus the scattering angle θ in polar coordinates. $P(q)$ shows a maximum value at minima of $S(q)$ and *vice versa*.

VIII --- Effect of Aggregation and Agglomeration, Sedimentation and Influence of Crystal Structure on ζ-Potential

Besides the changes of ζ-potential due to evolving surface chemistry, as discussed in Section 3.6., the changes of ζ-potential during non-equilibrium conditions may also be associated to one or any combination of the following processes: Aggregation and agglomeration, sedimentation and arising differences in crystal surface structure.[71] Before discussing these processes and how they may have affected the ζ-potential into more detail, it is important to note that changes in the particle size do not affect ζ-potential directly (Equation 3).

Aggregation and agglomeration could have influenced the ζ-potential indirectly, not because of the sudden particle size increase, but because of different electrokinetic properties of two or more particles that collide with each other (a phenomenon known as electroflocculation).[72] In addition, preferential aggregation or agglomeration of particles with favourable electrokinetic properties may have expounded the impact on ζ-potential. However, Ruiz-Agudo et al. (2014)[73] and Seepma et al. (2023)[74] showed that aggregation behaviour of barite crystals mainly occurred among particle sizes < 100 nm. So, we assumed little to no aggregation in the batch experiments occurred, as in most cases, the first DLS measurements for each experiment showed a particle size of 200 nm or larger. In addition, based on our extended-DLVO calculations (see Section 3.2, the discussion in SI-V and calculations in SI-VI), we believe that agglomeration in our very dilute samples, with a maximum solid volume fraction of ~ 0.013%, was negligible, as the average particle separation distance is ought to be in the range that is larger than the average particle size. Furthermore, Hang et al. (2009)[75] found that for similar NaCl background electrolyte concentrations in suspensions of barite particles are electrostatically stable at pH < 6, thus supporting our case.

An increase in average particle size may have promoted sedimentation, where sedimentation could have occurred when particles reached a size of about 700 nm.[74] Figure 3a-e shows strong evidence that sedimentation occurred, because, during each experiment, the absolute count rates decreased again (preceded by an initial increase), illustrated by the intensity of the yellow color. However, it is difficult to assess to

what extent this affected the apparent ζ-potential in our experiments. For example, if multiple populations of particles existed in the sample, but with a similar ζ-potential (i.e. with similar crystal surface structure and composition), then the apparent ζ-potential is unaffected by sedimentation. Causative is that the number of charges on the particle's surface driving the particle forward (in the electrical field) and the viscous drag on the particle are proportional to the surface area.[9] In other words, only the (average) particle size changes. However, if these settling particles are within the measurement window, then there is the possibility that they create an additional (velocity) vector other than the one induced by the electrical field and this may influence the apparent ζ-potential. In our types of samples, the apparent ζ-potential can also be affected by sedimentation as long as there were multiple populations present (i.e. at $r_{aq} = 1$; Figure 4c) with a different ζ-potential (Figure 3c) and where only one population may have grown larger than the other(s). Though we have no reason to assume that such a process took place, as neither of the two distinct ζ-potential distributions disappear suddenly (Figure 3c), differences in ζ-potential among the different particle size populations may have caused, for example, preferential aggregation and/or agglomeration and subsequent sedimentation of the circumneutral particles.

The crystal surface structure is important to translate the apparent ζ-potential to the potential at the surface. If the dominant crystal surface structure, especially its (micro-)roughness, changed as the system went to equilibrium, then the apparent zeta-potential, as Bikerman (1941)[76] pointed out, would need a correction, which would be different for each of the time steps (see also Wolthers et al. (2012)[77]). The crystal surface roughness of barite, caused by surface steps and other types of defect structures, leads to free energies that are significantly larger for such sites than those of perfect crystal faces. Past research has shown that the barite (001) surface displays a degree of heterogeneity, with the presence of defect structures like steps and etch pits, and cause an accumulation of ion species, which may alter the surface potential Ψ_s,[78-81] but cannot cause sign reversal.[81] Although the influence of surface roughness on the Ψ_s has not been investigated for barite, Na et al. (2007)[82] investigated this effect for cleaved $(10\bar{1}4)$ calcite surfaces. Using surface potential microscopy (i.e. a variant of non-contact atomic force microscopy), they showed that the Ψ_s is on average about 137 mV larger for nanostructure terraces of about 2 nm that exist on the substrate terraces. However, they observed about twice as much substrate terrace compared to nanostructure terrace (i.e. relative

frequency difference), even though they chose to measure along a 'xy'-line with a much larger nanostructure terrace density, because from their topography images it is clear that about 80% is substrate terrace. Due to the technique they used and their sample preparation, this large offset could be observed. The effect of surface roughness on colloidal particles immersed in an aqueous solution with a background electrolyte is much less (see Gan et al. (2012)[83]; i.e. their figure 8a), whom used numerical computations of the zeta potential using Monte Carlo simulations. For monovalent background electrolytes, with a concentration five times higher than our concentration (i.e. 0.1 M versus our 0.02 M), they showed that the difference is < 5 mV over a wide range of surface charge densities for a height difference of 0.4 nm. We consider that the extreme case presented by Na et al. (2007)[83] may only be important in systems where Ω_{barite} is continuously large, so that (2D-)nucleation is dominant. Under these conditions, adhesive growth on newly nucleated particles could create large differences in surface roughness. However, Kuwahara et al. (2016)[84] showed that Ω_{barite} needs to be larger than 1000 for 2D-nucleation to occur in the [001] direction (i.e. the crystal face that is most expressed) on a large scale. When $\Omega_{barite} < 1000$, the edge pits, kink sites and steps are filled with growth units or ions, so that the surface roughness disappears. Although, our BE concentration was less and their choice of the position of the slipping plane (i.e. about one ion away from the surface) is slightly different compared to our systems, it can be envisaged that the differences in ζ-potential was not more than 10 mV. Therefore, the differences in our case, potentially caused by surface roughness, were most likely captured in the distribution and error of the ζ-potential measurements themselves and most likely did not cause another distribution to appear in our measurements.

In our experiments, the average particle size was already far beyond the critical nucleus size of ~ 10 nm[74] and they grew most likely toward 600 – 700 nm by spiral growth.[84] While the role of surface roughness on ζ-potential was likely negligible in our experiments, crystal morphology cannot be ignored so easily. It is well known that for barite, different crystal morphology is developed at different values of r_{aq}.[85-87] Also, among different BEs and at the same r_{aq}-values, different morphology is developed.[88] However, it is less likely that in one particular experiment, more than one type of crystal morphology existed at the same time.[85] In that view, ζ-potential evolution in the experiments of Figure 3 could not be explained by crystal morphology and

we have discussed changes in ζ-potential during batch experiments in light of surface chemistry development.

IX --- Doppler Phase Shift & Voltage-Current Logging during Measurements with Varying r_{aq}

High quality data of the ζ-potential includes "smooth" Doppler phase curves, where the fast and slow field reversal are well distinguishable. This is the case for $r_{aq} \neq 1$ (Figure S9a, b, d and e). At $r_{aq} = 1$, there are some Doppler phase curves, which show "spaghetti-like" behaviour. These "spaghetti-like" curves represent the circumneutral population in Figure 3c. We expected that the ζ-potential at $r_{aq} = 1$ should be the closest to neutral compared to $r_{aq} \neq 1$ and, therefore, the particles have negligible preferences in moving direction upon the initiation of the electric field. In other words, this data is not necessarily poor in quality as this was something we expected to happen for $r_{aq} = 1$.

The Voltage-Current plots (Figure S9f-j) served as an indication of how much the folded capillary cell (in which we measured ζ-potential throughout the batch experiment) underwent degradation. This was important, because we measured 30 times the ζ-potential consecutively. The purple color in those plots is the average voltage during one ζ-potential measurement (usually consisting of 20-25 subruns). With the constant voltage mode, this is always the same for any ζ-potential measurement. The red and the green color represent the current during the first and last ζ-potential measurement. The current is slightly less for the last measurement compared to the initial measurement and is a consequence of degradation at both ends of the electrodes of the cell. Degradation of the cell during repetition of ζ-potential measurements is a normal process and cannot be avoided. However, extensive degradation of the electrodes would lower the current drastically or even change the shape of the current curves. Since we did not observe such behaviour in Figure S9f-j, the performed ζ-potential measurements were of high quality.

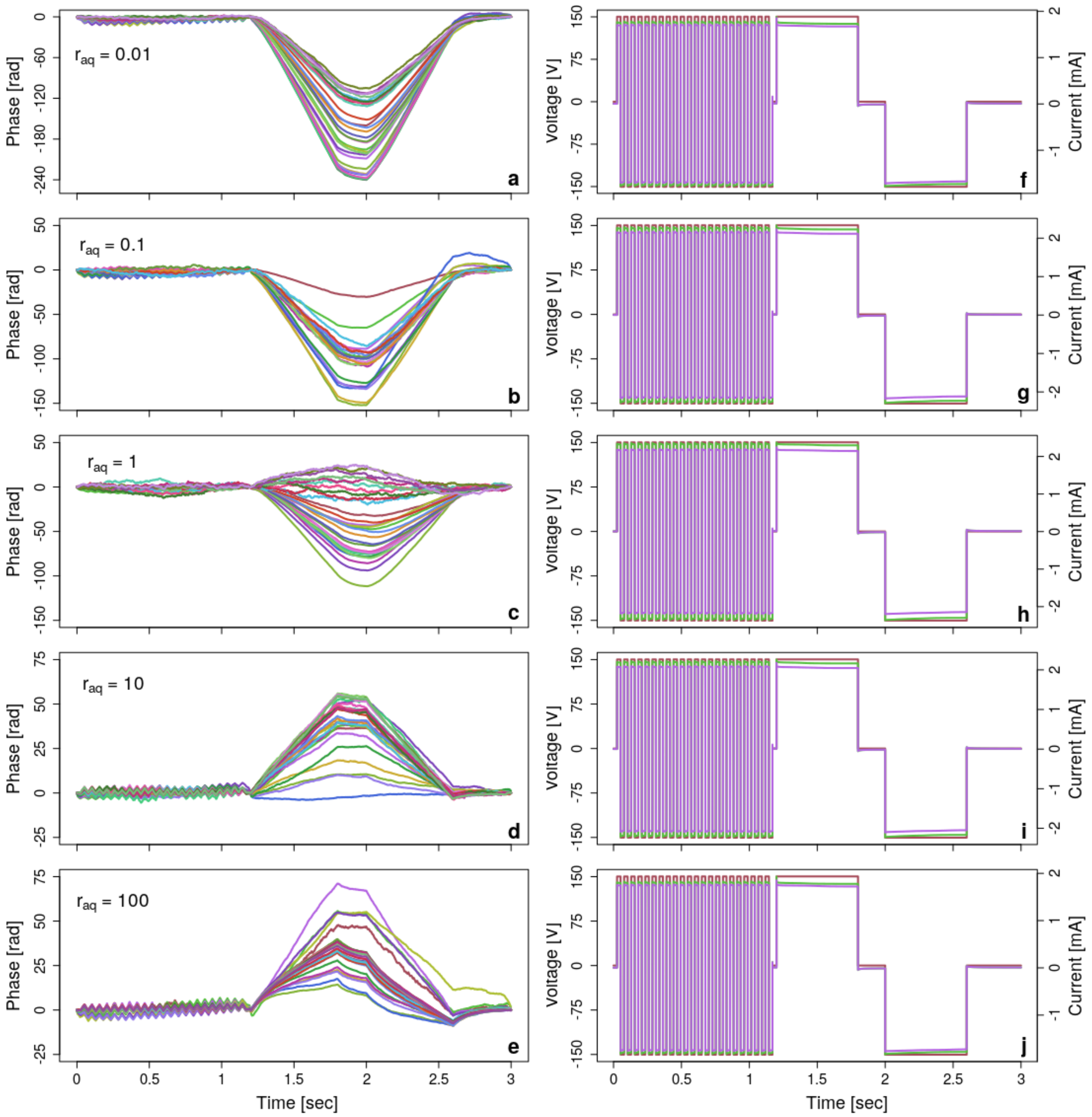

Figure S9: Doppler phase plots, showing the phase shift between the measured beat frequency (scattered beam) and the reference frequency as a function of time (a-e) and the Voltage-Current logging (f-j), coinciding with the experiments presented in Figure 3 (i.e. initial Ω_{barite} = 1000, varying r_{aq}). r_{aq} increases from top to bottom; 0.01, 0.1, 1, 10 and 100.

X --- BSD Size Measurements Performed Routinely in-between ζ-Potential Measurements

Some size measurements for every r_{aq} were performed during the batch ζ-potential experiments (Figures S10-S14). These size measurements were performed to check whether the applied electric field caused unusual changes in d_p. The application of an electric field may have a variety of consequences during crystal formation. Examples include: Reduced nucleation times, increased nucleation rates, less crystals formed but with enlarged sizes, different orientation of crystals, increased crystallization yield and alteration of transformation processes affecting polymorphism (see review of Alexander & Radacsi (2019)[89] and references therein). The DLS measurements in between ζ-potential measurements were performed in BSD, because it was more suitable to detect different size populations in the smaller range of d_p and we were only interested if the d_p was in the range that we measured in FWD where no electric field was imposed (i.e. between 100 and 1000 nm; Figure 3f-j) and not in the very precise hydrodynamic size. Besides the very first measurement ($t \sim 0$), which was dominated by the highly stochastic nature of crystal nucleation, the size range for $t \neq 0$, dominated by crystal growth, was indeed in the same size range as was measured by FWD without an applied electric field and we assumed that the applied electric field did not significantly influence the particles in the investigated systems.

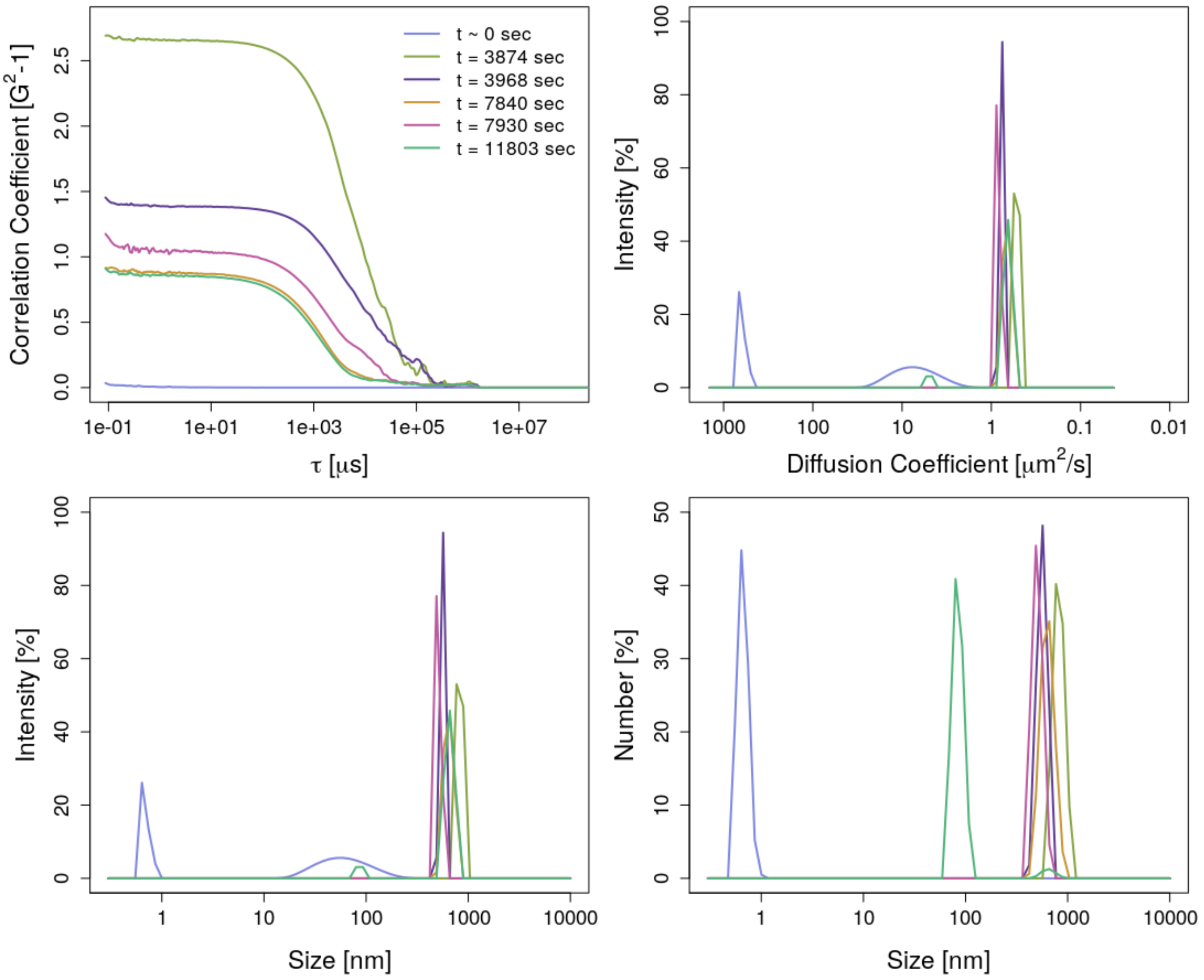

Figure S10: Displays the autocorrelation curves (a), the diffusion coefficient distribution (b), the intensity size distribution (c) and the number size distribution (d) for DLS measurements performed at specific time steps (see legend) during the ζ-potential batch experiment at initial Ω_{barite} = 1000 and r_{aq} = 0.01.

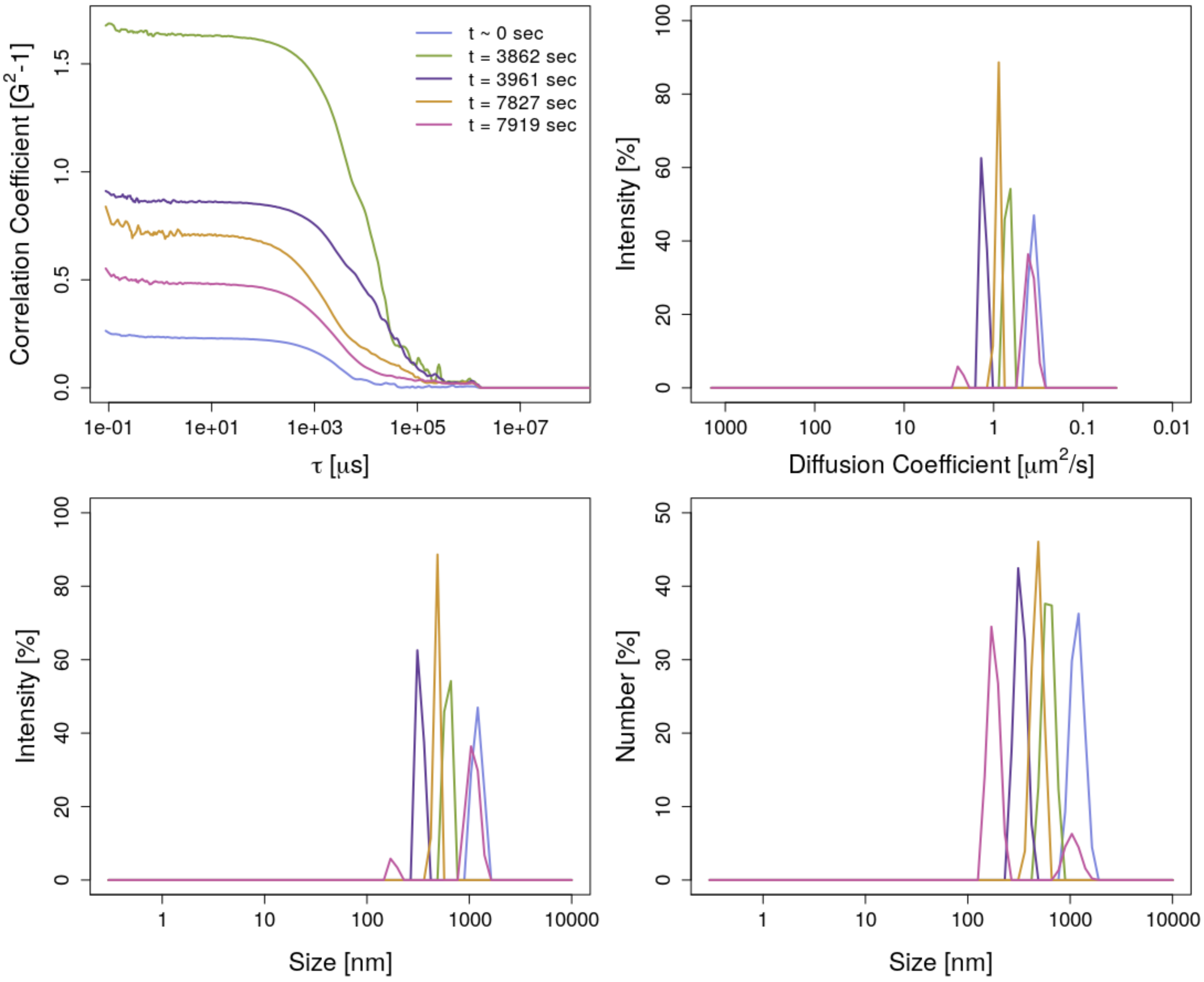

Figure S11: Displays the autocorrelation curves (a), the diffusion coefficient distribution (b), the intensity size distribution (c) and the number size distribution (d) for DLS measurements performed at specific time steps (see legend) during the ζ-potential batch experiment at initial Ω_{barite} = 1000 and r_{aq} = 0.1.

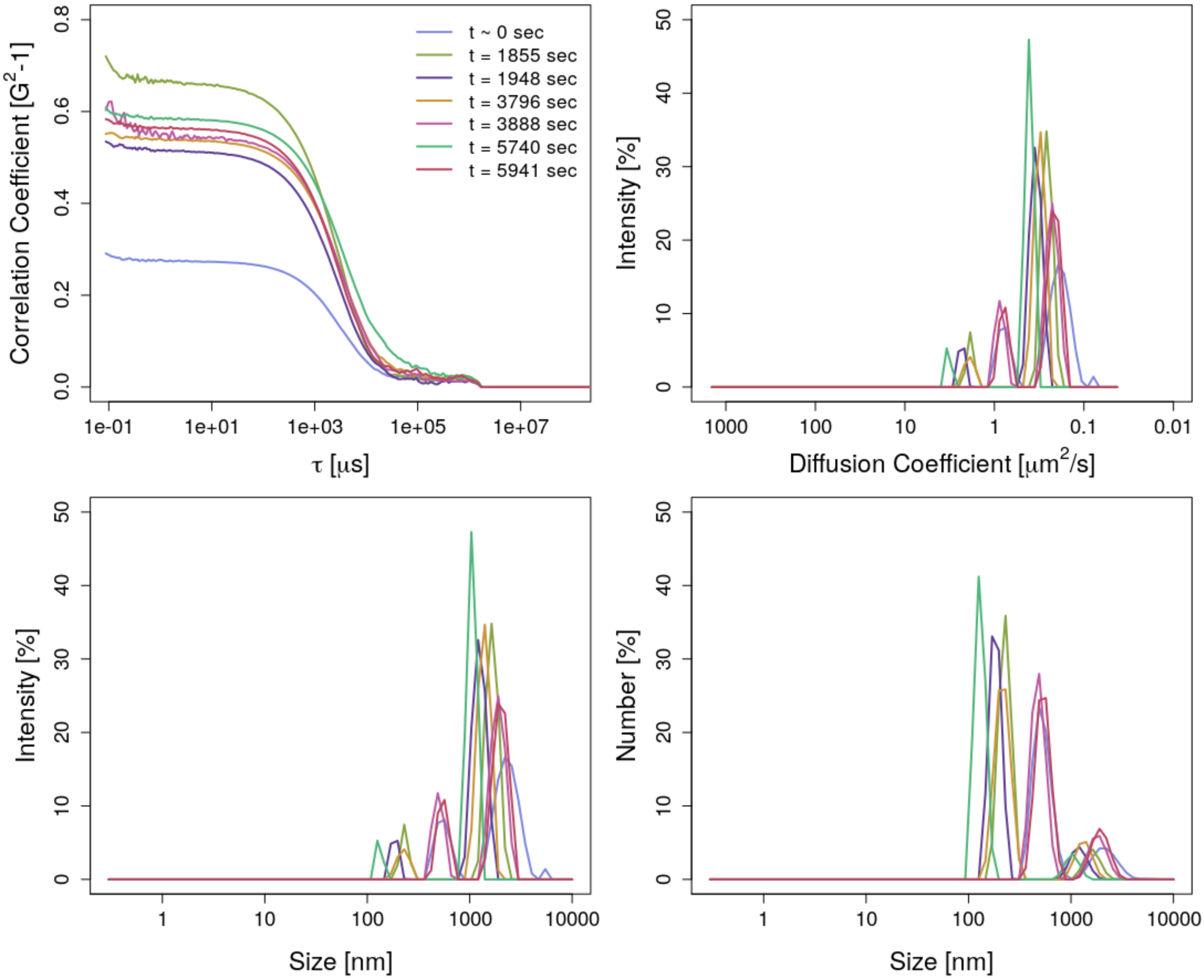

Figure S12: Displays the autocorrelation curves (a), the diffusion coefficient distribution (b), the intensity size distribution (c) and the number size distribution (d) for DLS measurements performed at specific time steps (see legend) during the ζ-potential batch experiment at initial Ω_{barite} = 1000 and r_{aq} = 1.

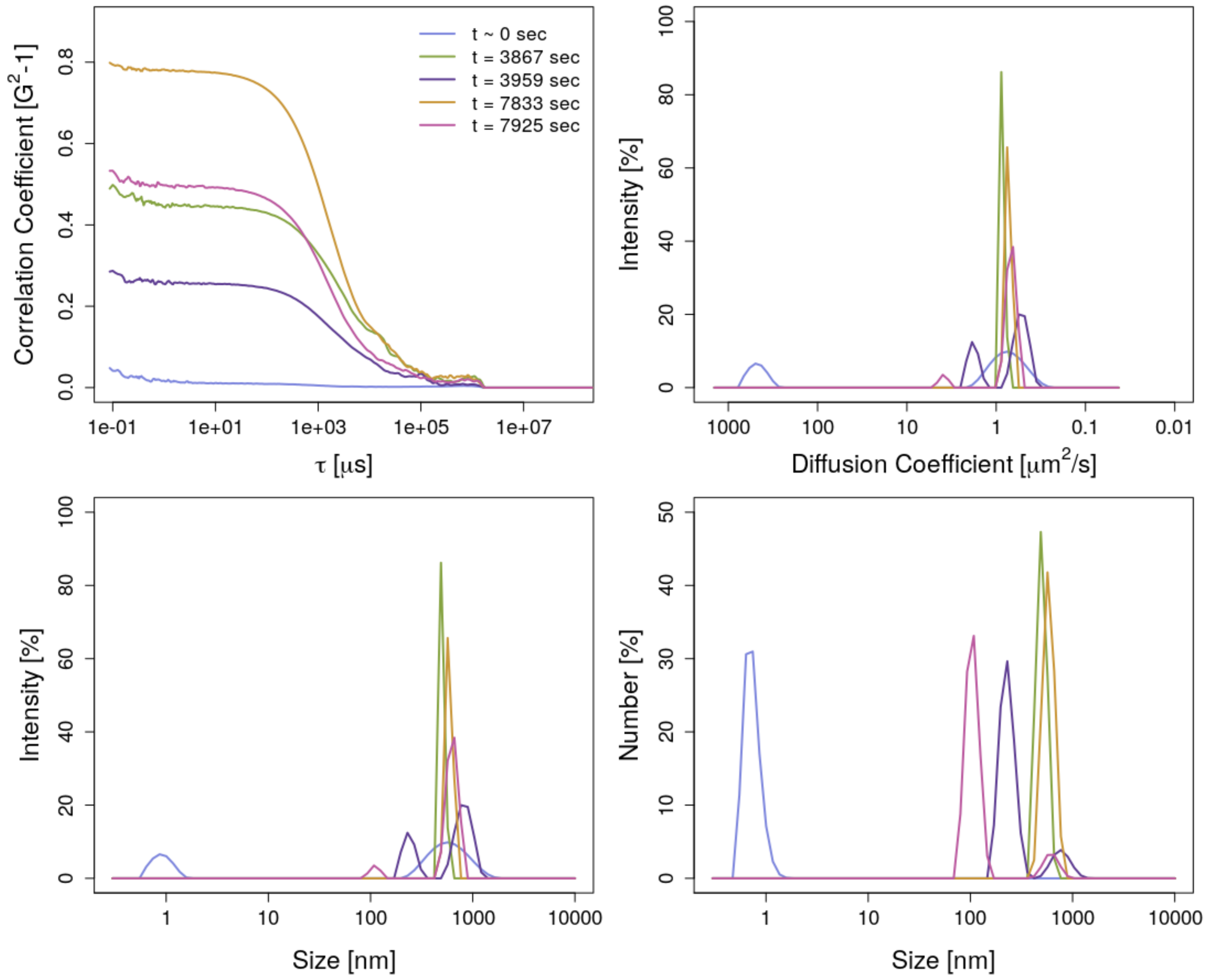

Figure S13: Displays the autocorrelation curves (a), the diffusion coefficient distribution (b), the intensity size distribution (c) and the number size distribution (d) for DLS measurements performed at specific time steps (see legend) during the ζ-potential batch experiment at initial Ω_{barite} = 1000 and r_{aq} = 10.

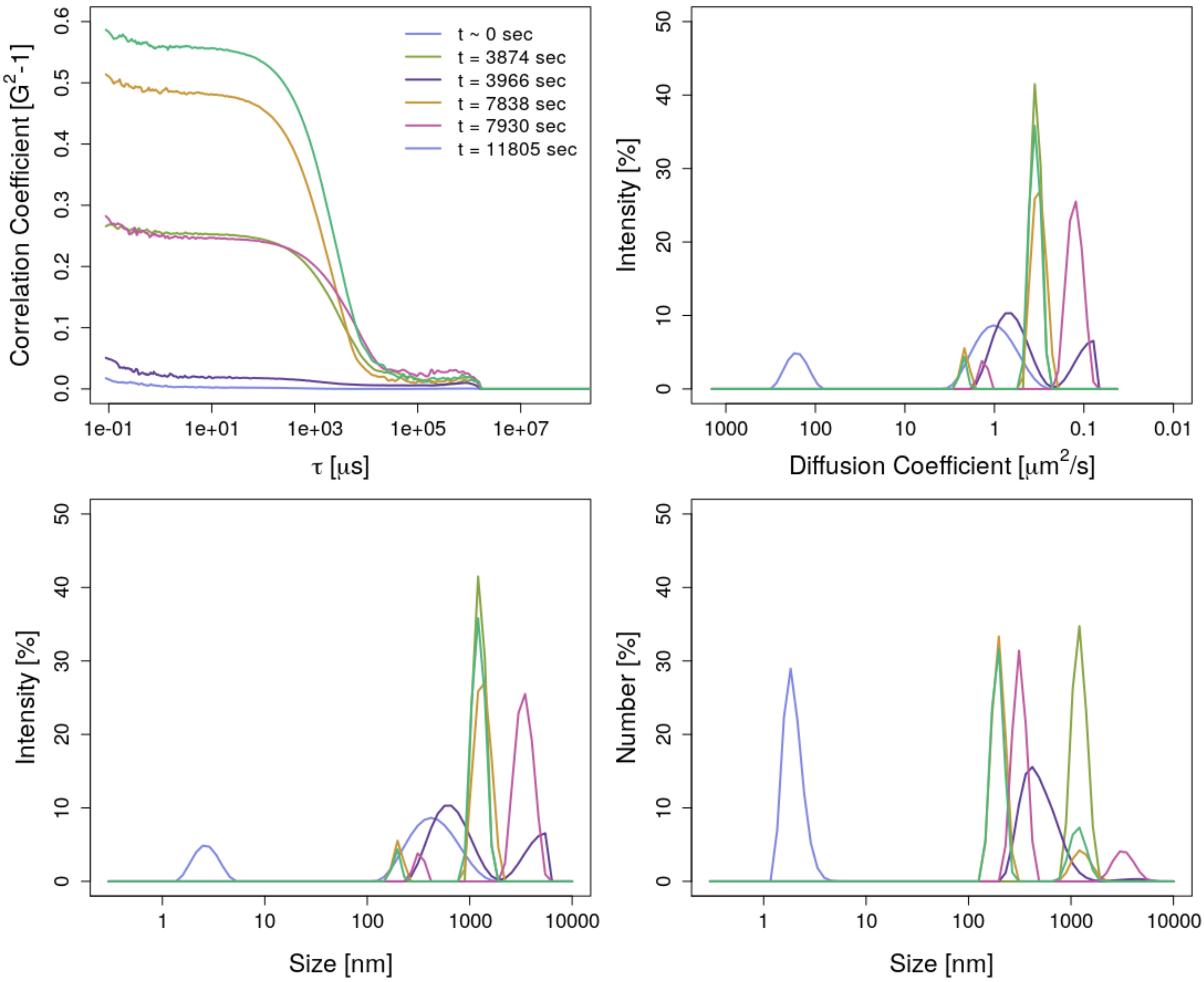

Figure S14: Displays the autocorrelation curves (a), the diffusion coefficient distribution (b), the intensity size distribution (c) and the number size distribution (d) for DLS measurements performed at specific time steps (see legend) during the ζ-potential batch experiment at initial Ω_{barite} = 1000 and r_{aq} = 100.

XI --- Particle Size Distributions for Other Conditions

Similarly to Figure 4, Figure S15 shows the same trend in intensity for backscattering detection angle (BSD) measurements; sometimes a particle size distribution at < 10 nm at t = 0 seconds was observed, meaning conditions of nucleation and growth occurred.

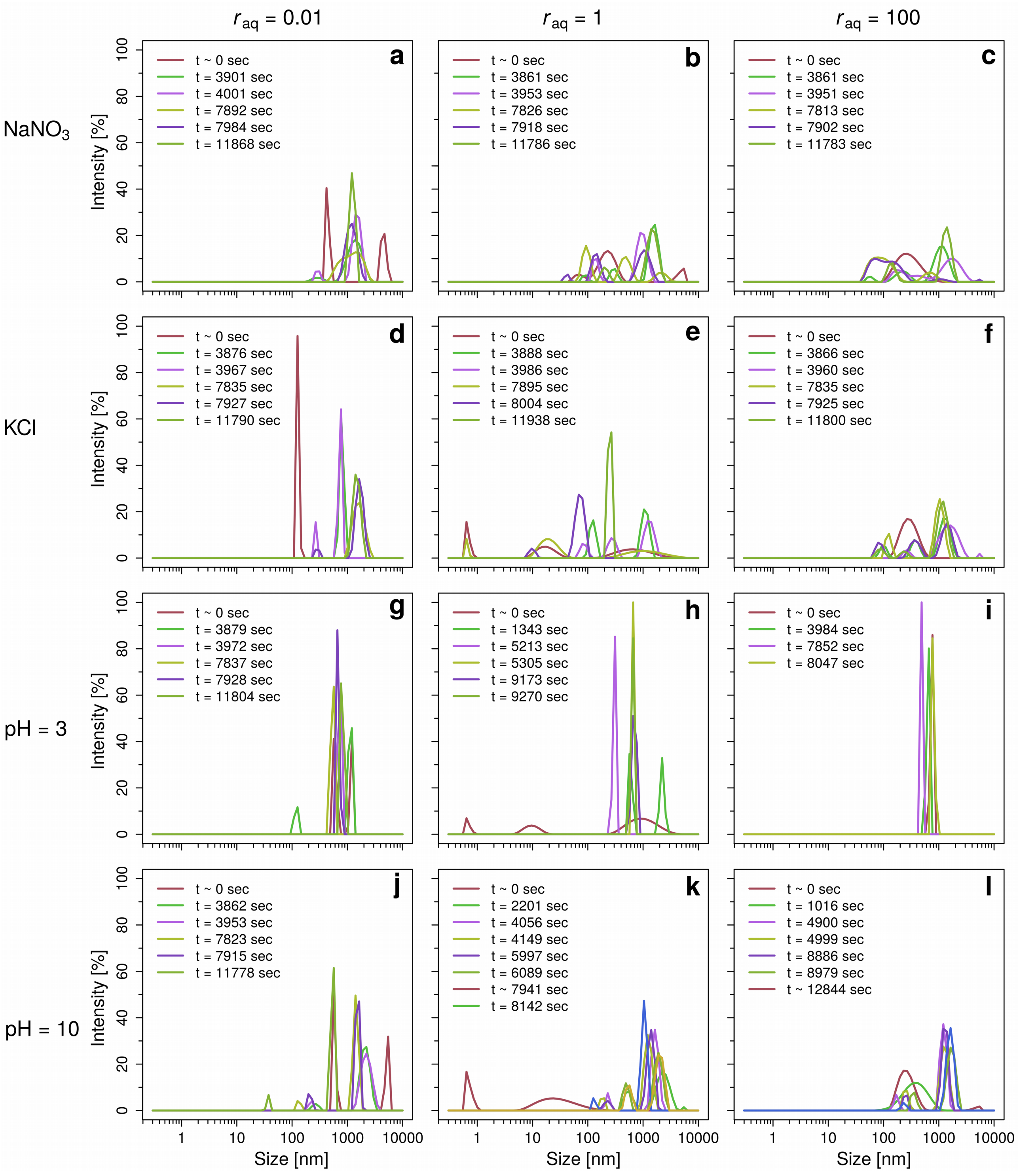

Figure S15: Intensity particle size distributions during the continuous ζ-potential measurements for solution nos. 5.1 (a), 5.2 (b), 5.3 (c), 4.1 (d), 4.2 (e), 4.3 (f), 3.2 (g), 3.1 (h), 3.3 (i), 3.5 (j), 3.4 (k) and 3.6 (l). The time steps are defined by color.

XII --- The Effect of pH on the ζ-Potential for Different r_{aq} Conditions

Batch experiments were performed at different conditions of pH to investigate the effect of pH on the formation of $BaSO_4$ at different r_{aq}. Note that Figure S16d-f are respectively the same as the ones displayed in Figure 3a, c and e in the main text.

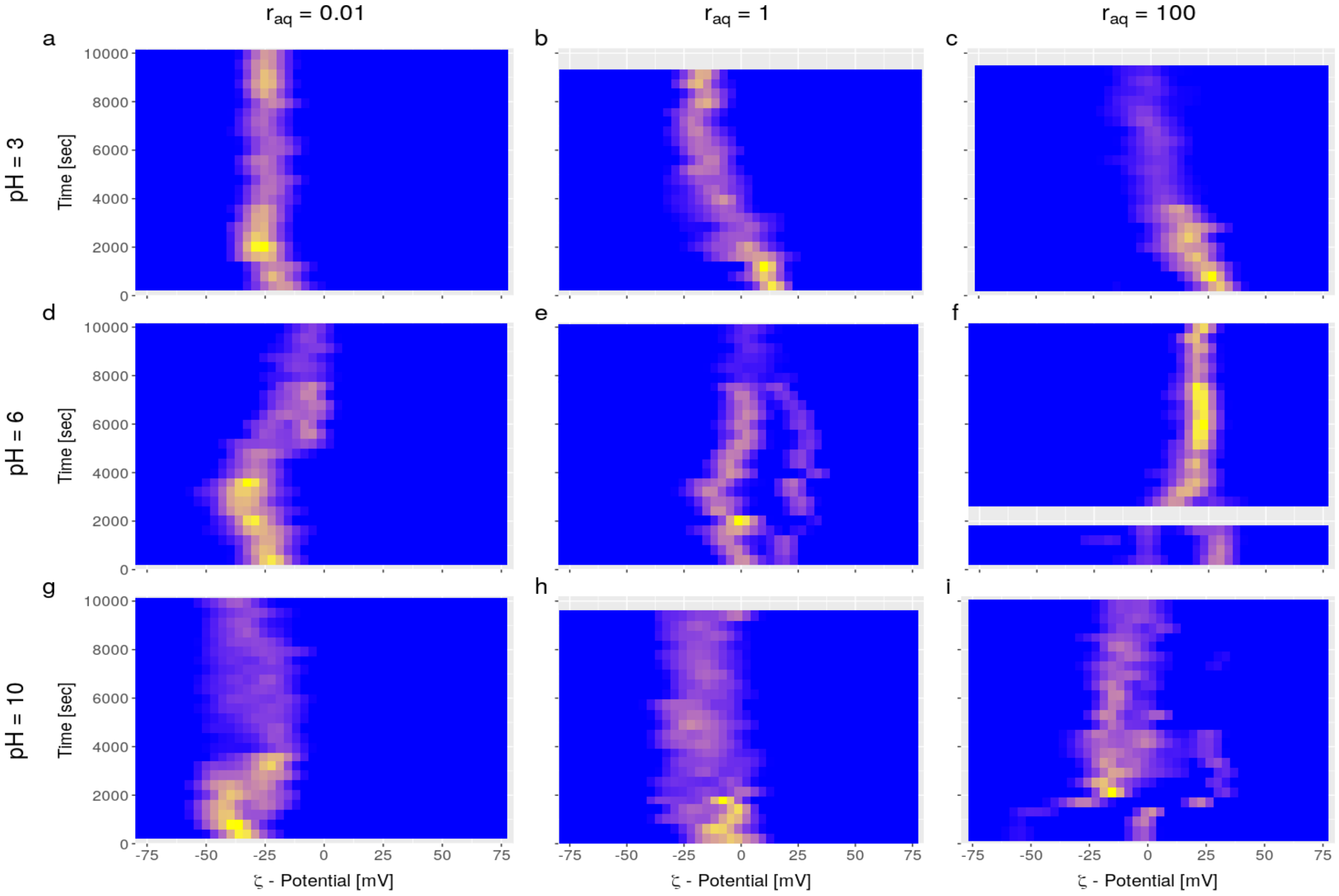

Figure S16: Evolution in ζ-potential with time at pH = 3 for r_{aq} = 0.01 (a), r_{aq} = 1 (b) and r_{aq} = 100 (c) and at pH ~6 for r_{aq} = 0.01 (d), r_{aq} = 1 (e) and r_{aq} = 100 (f) and at pH = 10 for r_{aq} = 0.01 (g), r_{aq} = 1 (h) and r_{aq} = 100 (i). All experiments were performed at an initial Ω_{barite} = 1000 and I = 0.02 M. The measurements have been conducted for roughly three hours. Note that a minor data gap is present for pH = 6 and r_{aq} = 100 due to the zetasizer running into an unknown error during the measurement.

XIII --- The Effect of Different Monovalent BEs on the ζ-Potential for Different r_{aq} Conditions

Batch experiments were performed at different conditions of pH to investigate the effect of pH on the formation of $BaSO_4$ at different r_{aq}. Note that Figure S17d-f are respectively the same as the ones displayed in Figure 3a, c and e in the main text.

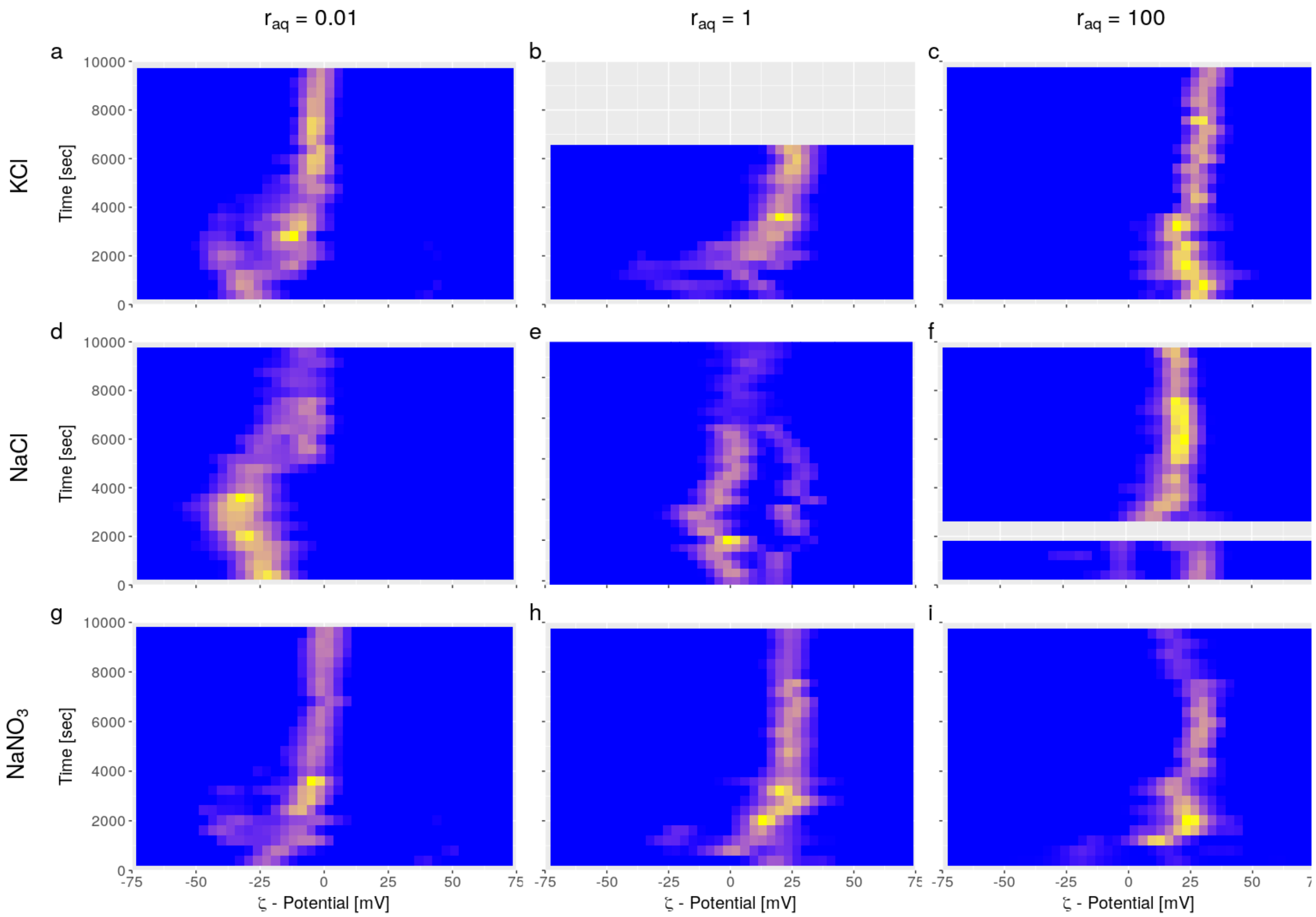

Figure S17: Evolution in ζ-potential with time at initial Ω_{barite} = 1000, pH ~ 6 and *I* = 0.02 M for KCl at r_{aq} = 0.01 (a), 1 (b) and 100 (c) and for NaCl at r_{aq} = 0.01 (d), 1 (e) and 100 (f) and for $NaNO_3$ at r_{aq} = 0.01 (g), 1 (h) and 100 (I). The measurements have been conducted for roughly three hours. Note that minor data gaps are present due to the zetasizer running into an unknown error during those measurements.

References

[1] Clogston, J. D.; Patri, A. K. Zeta Potential Measurements. In *Characterization of nanoparticles intended for drug delivery. Chapter 6 Zeta Potential Measurement*; McNeil, S. Eds.; Humana Press: Clifton, NJ, 2011: pp 63-70.

[2] Zeebe, R. E.; Wolf-Gladrow, D. *CO_2 in Seawater: Equilibrium, Kinetics, Isotopes, Chapter 2 Kinetics*; Gulf Professional Publishing: Houston, TX, 2001: pp 85-140.

[3] Lawless, W. N.; Devries, R. C. Oxygen polarizability and point-dipole theory in the carbonate minerals. *J. Phys. Chem. Solids* **1964**, *25*(10), 1119-1124.

[4] Bucca, M.; Dietzel, M.; Tang, J.; Leis, A.; Köhler, S. J. Nucleation and crystallization of otavite, witherite, calcite, strontianite, hydrozincite, and hydrocerussite by CO_2 membrane diffusion technique. *Chem. Geol.* **2009**, *266*(3-4), 143-156.

[5] Hückel, E.; Debye, P. Zur Theorie der Elektrolyte. I. Gefrierpunktserniedrigung und verwandte Erscheinungen. *Phys. Z.* **1923**, *24*(9), 185-206.

[6] Deshiikan, S. R.; Papadopoulos, K. D. Modified Booth equation for the calculation of zeta potential. *Colloid Polym. Sci.* **1998**, *276*, 117-124.

[7] Ohshima, H. A simple expression for henry's function for the retardation effect in electrophoresis of spherical colloidal particles. *J. Colloid Interface Sci.* **1994**, *168*(1), 269-271.

[8] Henry, D. C. The cataphoresis of suspended particles. Part I.—The equation of cataphoresis. *Proc. R. Soc. London, Ser. A* **1931**, *133*(821), 106-129.

[9] Hunter, R. J. *Colloid science: zeta potential in colloid science: principles and applications*; London Academic Press: UK, 1981.

[10] Lang, P. F.; Smith, B. C. Ionic radii Group 1 and Group 2 halide, hydride, fluoride, oxide, sulfide, selenide and telluride crystals. *Dalton Trans.* **2010**, *39*(33), 7786-7791.

[11] Elghannay, H.; Tafti, D.; Yu, K. Evaluation of physics based hard-sphere model with the soft sphere model for dense fluid-particle flow systems. *Int. J. Multiphase Flow* **2019**, *112*, 100-115.

[12] Revil, A.; Hermitte, D.; Spangenberg, E.; Cocheme, J. J. Electrical properties of zeolitized volcaniclastic materials. *J. Geophys. Res.: Solid Earth* **2002**, *107*(B8), ECV-3.

[13] Li, S.; Leroy, P.; Heberling, F.; Devau, N.; Jougnot, C.; Chiaberge, C. Influence of surface conductivity on the apparent zeta potential of calcite. *J. Colloid Interface Sci.* **2016**, *468*, 262-275.

[14] Delgado, Á. V.; González-Caballero, F.; Hunter, R. J.; Koopal, L.; Lyklema, J. Measurement and interpretation of electrokinetic phenomena. *J. Colloid Interface Sci.* **2007**, *309*(2), 194-224.

[15] Youssefi, R. M.; Diez, F. J. Ultrafast electrokinetics. *Electrophoresis* **2016**, *37*(5-6), 692-698.

[16] Dukhin, S. S. Diffusion-electrical theory of electrophoresis. XX*th Int. Congr. Pure Appl. Chem.*, Moscow, USSR, 12-18 July, 1965.

[17] Derjaguin, B.V.; Dukhin, S. S.; Matijevic, E. Nonequilibrium double layer and electrokinetic phenomena. *Surf. Colloid Sci.* **1974**, *7*, 273-335.

[18] Bikerman, J. J. Ionentheorie der Elektrosmose, der Strömungsströme und der Oberflächenleitfähigkeit. *Z. Phys. Chem.* **1933**, *163*(1), 378-394.

[19] Kadhim, M. J.; Gamaj, M. I. Estimation of the Diffusion Coefficient and Hydrodynamic Radius (Stokes Radius) for Inorganic Ions in Solution Depending on Molar Conductivity as Electro-Analytical Technique - A Review. *J. Chem. Rev.* **2020**, *2*(3), 182-188.

[20] Bolève, A.; Crespy, A.; Revil, A.; Janod, F.; Mattiuzzo, J. L. Streaming potentials of granular media: Influence of the Dukhin and Reynolds numbers. *J. Geophys. Res.: Solid Earth* **2007**, *112*(B8), B08204.

[21] Leroy, P.; Revil, A. A mechanistic model for the spectral induced polarization of clay materials. *J. Geophys. Res.: Solid Earth* **2009**, *114*(B10), B10202.

[22] Jiménez, M. L.; Arroyo, F. J.; Carrique, F.; Kaatze, U.; Delgado, A. V. Determination of stagnant layer conductivity in polystyrene suspensions: temperature effects. *J. Colloid Interface Sci.* **2005**, *281*(2), 503-509.

[23] Ohshima, H. The Derjaguin-Landau-Verwey-Overbeek (DLVO) Theory of Colloid Stability. In *Electrical Phenomena at Interfaces and Biointerfaces: Fundamentals and Applications in Nano-, Bio- and Environmental Sciences*; John Wiley & Sons Inc.: Hoboken, NJ, 2012: pp 27-34.

[24] Israelachvili, J. *Intermolecular & surface forces*; London Academic Press: London, UK, 1992.

[25] Van Oss, C. J. *Interfacial forces in aqueous media*: Marcel Dekker: New York, NY, 1994.

[26] Van Roij, R.; Dijkstra, M., Hansen, J. P. Phase diagram of charge-stabilized colloidal suspensions: van der Waals instability without attractive forces. *Phys. Rev. E* **1999**, *59*(2), 2010-2025.

[27] Durán, J. D. G.; Ontiveros, A.; Delgado, A. V.; González-Caballero, F.; Chibowski, E. A study on the adhesion of calcium carbonate to glass. Energy balance in deposition process. *J. Adhes. Sci. Technol.* **1996**, *10*(9), 847-868.

[28] Vera, P.; Gallardo, V.; Salcedo, J.; Delgado, A. V. Colloidal stability of a pharmaceutical latex: experimental determinations and theoretical predictions. *J. Colloid Interface Sci.* **1996**, *177*(2), 553-560.

[29] Tagawa, M.; Gotoh, K. Detergency. In *Electrical phenomena at interfaces*; Ohshima, H.; Furusawa, K., Eds.; Marcel Dekker: New York, NY, 1998: pp 358-374.

[30] Hogg, R.; Healy, T. W.; Fuerstenau, D. W. Mutual coagulation of colloidal dispersions. *Trans. Faraday Soc.* **1966**, *62*, 1638-1651.

[31] Pugh, R. J.; Kitchener, J. A. Theory of selective coagulation in mixed colloidal suspensions. *J. Colloid Interface Sci.* **1971**, *35*(4), 656-664.

[32] Ohshima, H. Interaction of electrical double layers. In *Electrical phenomena at interfaces*; Ohshima, H.; Furusawa, K., Eds.; Marcel Dekker: New York, NY, 1998: pp 57-86.

[33] Gallardo V.; Zurita, L.; Ontiveros, A.; Durán, J. D. G. Interfacial properties of barium sulfate suspensions. Implications in their stability. *J. Pharm. Sci.* **2000**, *89*(9), 1134-1142.

[34] Wang, X.; Zhang, Q. Insights into the Influence of Surface Roughness on the wettability of Apatite and Dolomite. *Minerals* **2020**, *10*(2), 114.

[35] Lee, L. H. Correlation between Lewis acid- base surface interaction components and linear solvation energy relationship solvatochromic α and β parameters. *Langmuir* **1996**, *12*(6), 1681-1687.

[36] Giese, R. F.; Constanzo, P. M.; van Oss, C. J. The surface free energies of talc and pyrophyllite. *Phys. Chem. Miner.* **1991**, *17*(7), 611-616.

[37] Chibowsky, E.; Holysz, L. Use of the Washburn equation for surface free energy determination. *Langmuir* **1992**, *8*(2), 710-716.

[38] Chibowski, E.; González-Caballero, F. Theory and practice of thin-layer wicking. *Langmuir* **1993**, *9*(1), 330-340.

[39] Verwey, E. J. W.; Overbeek, J. T. G. Theory of the stability of lyophobic colloids. *J. Colloid Sci.* **1955**, *10*(2), 224-225.

[40] Schenkel, J. H.; Kitchener, J. A. A test of the Derjaguin-Verwey-Overbeek theory with a colloidal suspension. *Trans. Faraday Soc.* **1960**, *56*, 161-173.

[41] Verwey, E. J. W; Overbeek, J. T. G. *Theory of the Stability of Lyophobic Colloids: The Interaction of Soil Particles having an Electric Double Layer*; Elsevier Publishing Company, Inc.: Amsterdam, The Netherlands, 1948.

[42] Hamaker, H. C. The London – van der Waals attraction between spherical particles. *Physica* **1937**, *4*(10), 1058-1072.

[43] Bergström, L. Hamaker constants of inorganic materials. *Adv. Colloid Interface Sci.* **1997**, *70*, 125-169.

[44] Van Oss, C. J.; Giese, R. F.; Costanzo, P. M. DLVO and non-DLVO interactions in hectorite. *Clays Clay Miner.* **1990**, *38*(2), 151-159.

[45] Farmakis, L.; Lioris, N.; Koliadima, A.; Karaiskakis, G. Estimation of the Hamaker constants by sedimentation field-flow fractionation. *J. Chromatogr.* **2006**, *1137*(2), 231-242.

[46] Israelachvili, J.; Pashley, R. The hydrophobic interaction is long range, decaying exponentially with distance. *Nature* **1982**, *300*(5890), 341-342.

[47] Claesson, P. M.; Blom, C. E.; Herder, P. C.; Ninham, B. W. Interactions between water-stable hydrophobic Langmuir-Blodgett monolayers on mica. *J. Colloid Interface Sci.* **1986**, *114*(1), 234-242.

[48] Chan, D. Y. C.; Mitchell, D. J.; Ninham, B. W.; Pailthorpe, B. A. Dispersion interactions across binary liquid mixtures. A proper account of structural effects. *J. Colloid Interface Sci.* **1979**, *68*(3), 462-470.

[49] Christenson, H. K.; Claesson, P. M. Cavitation and the interaction between macroscopic hydrophobic surfaces. *Science* **1988**, *239*(4838), 390-392.

[50] Van Oss, C. J. *Interfacial Forces in Aqueous Media* (2nd Ed.); Marcel Dekker: New York, NY, 2006.

[51] Yao, J.; Han, H.; Hou, Y.; Gong, E.; Yin, W. A Method of Calculating the Interaction Energy between Particles in Minerals Flotation. *Math. Probl. Eng.* **2016**, *2016*, 8430745.

[52] Good, R. J. Contact angle, wetting, and adhesion: a critical review. *J. Adhes. Sci. Technol.* **1992**, *6*(12), 1269-1302.

[53] Van Oss, C. J.; Chaudhury, M. K.; Good, R. J. Monopolar surfaces. *Adv. Colloid Interface Sci.* **1987**, *28*, 35-64.

[54] Wu, W. Nancollas, G. H. A New Understanding of the Relationship Between Solubility and Particle Size. *J. Solution Chem.* **1998**, *27*(6), 521-531.

[55] Vijayabaskar, M. S.; Vishveshwara, S. Interaction energy based protein structure networks. *Biophys. J.* **2010**, *99*(11), 3704-3715.

[56] Posner, J. D. Properties and electrokinetic behavior of non-dilute colloidal suspensions. *Mech. Res. Commun.* **2009**, *36*(1), 22-32.

[57] Patterson, G. D.; Jarry, J. P.; Lindsey, C. P. Photon Correlation Spectroscopy of Polystyrene Solutions. *Macromolecules* **1980**, *13*(3), 668-670.

[58] Candau, S. J.; Butler, I.; King, T. A. Co-operative diffusion of semi-dilute solutions, concentrated solutions and swollen networks for polystyrene in dibutylphthalate. *Polymer* **1983**, *24*(12), 1601-1609.

[59] Michaelides, E. E. Wall effects on the Brownian movement, thermophoresis, and deposition of nanoparticles in liquids. *J. Fluids Eng.* **2016**, *138*(5), 051303.

[60] Marra, A.; Peuvrel-Disdier, E.; Wittemann, A.; Guo, X.; Ballauff, M. Rheology of dilute and semidilute suspensions of spherical polyelectrolyte brushes. *Colloid Polym. Sci.* **2003**, *281*(6), 491-496.

[61] Mewis, J.; Wagner, N. J. *Colloidal Suspension Rheology*; Cambridge University Press: London, UK, 2011.

[62] Hao, T.; Riman, R. E. Calculation of interparticle spacing in colloidal systems. *J. Colloid Interface Sci.* **2006**, *297*(1), 374-377.

[63] Abbott, S.; Holmes, N. *Nanocoatings: Principles and Practice: From Research to Production*; DEStech Publications Inc.: Lancaster, PA, 2013.

[64] Huang, X; Bhattacharjee, S.; Hoek, E. M. Is surface roughness a "scapegoat" or a primary factor when defining particle-substrate interactions? *Langmuir* **2010**, *26*(4), 2528-2537.

[65] Schärtl, W. *Light scattering from polymer solutions and nanoparticle dispersions*; Springer: Heidelberg, Germany, 2007.

[66] Hammouda, B. *Probing Nanoscale Structures – The SANS Toolbox*; National Institute of Standards and Technology, Center for Neutron Research, Gaithersburg, MD, 2016. Available at: http://www.ncnr.nist.gov/staff/hammouda/the_SANS_toolbox.pdf

[67] Ornstein, L.; Zernike, F. Die linearen dimensionen der dichteschwankungen. *Phys. Z.* **1918**, *19*, 134.

[68] Pusey, P. N.; van Megen, W. Phase behaviour of concentrated suspensions of nearly hard colloidal spheres. *Nature* **1986**, *320*, 340-342.

[69] Wertheim, M. Exact Solution of the Percus-Yevick Integral Equation for Hard Spheres. *Phys. Rev. Lett.* **1963**, *10*(8): 321-323.

[70] Percus, J. K.; Yevick, G. J. Analysis of classical statistical mechanics by means of collective coordinates. *Phys. Rev.* **1958**, *110*(1), 1-13.

[71] Lowry, G. V.; Hill, R. J.; Harper, S.; Rawle, A. F.; Hendren, C. O.; Klaessig, F.; Nobbmann, U; et al. Guidance to improve the scientific value of zeta-potential measurements in nanoEHS. *Environ. Sci.: Nano* **2016**, *3*(5), 953-965.

[72] Ofir, E.; Oren, Y.; Adin, A. Electroflocculation: the effect of zeta-potential on particle size. *Desalinisation* **2007**, *204*(1-3), 33-38.

[73] Ruiz-Agudo, E.; Putnis, C. V.; Putnis, A. The effect of a copolymer inhibitor on baryte precipitation. *Mineral. Mag.* **2014**, *78*(6), 1423-1430.

[74] Seepma, S. Y. M. H.; Kuipers, B. W. M.; Wolthers, M. Asymmetrical Dependence of {Ba^{2+}}:{SO_4^{2-}} on $BaSO_4$ Crystal Nucleation and Growth in Aqueous Solutions: A Dynamic Light Scattering Study. *ACS Omega* **2023**, *8*(6), 5760-5775.

[75] Hang, J.; Shi, L.; Feng, X.; Xiao, L. Electrostatic and electrosteric stabilization of aqueous suspensions of barite nanoparticles. *Power Technol.* **2009**, *192*(2), 166-170.

[76] Bikerman, J. J. Immobile Layer at Solid-Liquid Interface. *J. Chem. Phys.* **1941**, *9*(12), 880.

[77] Wolthers, M.; Di Tommaso, D.; Du, Z.; de Leeuw, N. H. Calcite surface structure and reactivity: molecular dynamics simulations and macroscopic surface modelling of the calcite-water interface. *Phys. Chem. Chem. Phys.* **2012**, *14*(43), 15145-15157.

[78] Higgins, S. R.; Jordan, G.; Eggleston, C. M.; Knauss, K. G. Dissolution kinetics of the barium sulfate (001) surface by hydrothermal atomic force microscopy. *Langmuir* **1998**, *14*(18), 4967-4971.

[79] Fenter, P.; McBride, M. T.; Srajer, G.; Sturchio, N. C.; Bosbach, D. Structure of barite (001)- and (210)- water interfaces. *J. Phys. Chem. B* **2001**, *105*(34), 8112-8119.

[80] Bokern, D. G.; Ducker, W. A. C.; Hunter, K. A.; McGrath, K. M. Surface imaging of a natural mineral surface using scanning-probe microscopy. *J. Cryst. Growth* **2002**, *246*(1-2), 139-149.

[81] Bokern, D. G.; Hunter, K. A.; McGrath, K. M. Charged barite− aqueous solution interface: Surface potential and atomically resolved visualization. *Langmuir* **2003**, *19*(24), 10019-10027.

[82] Na, C.; Kendall, T. A.; Martin, S. T. Surface-potential heterogeneity of reacted calcite and rhodochrosite. *Environ. Sci. Technol.* **2007**, *41*(18), 6491-6497.

[83] Gan, Z.; Xing, X.; Xu, Z. Effects of image charges, interfacial charge discreteness, and surface roughness on the zeta potential of spherical electric double layers. *J. Chem. Phys.* **2012**, *137*(3), 034708.

[84] Kuwahara, Y.; Liu, W.; Makio, M.; Otsuka, K. In Situ AFM Study of Crystal Growth on a Barite (001) Surface in $BaSO_4$ Solutions at 30 °C. *Minerals* **2016**, *6*(4), 117.

[85] Voigt, A; Sundmacher, K. Monte Carlo simulation of shape evolution in solutions – A model study of $BaSO_4$ precipitation. In *Computer Aided Chemical Engineering* **2012**, *30*, 1163-1166.

[86] Bracco, J. N.; Gooijer, Y.; Higgins, S. R. Hydrothermal atomic force microscopy observations of barite step growth rates as a function of the aqueous barium-to-sulfate ratio. *Geochim. Cosmochim. Acta* **2016**, *183*, 1-13.

[87] Wang, C.; Zhou, L.; Zhang, S.; Wang, L.; Wei, C.; Song, W.; Xu, L.; Zhou, W. Morphology of Barite Synthesized by In-Situ Mixing of Na_2SO_4 and $BaCl_2$ Solutions at 200 °C. *Crystals* **2021**, *11*(8), 962.

[88] Kowacz, M.; Putnis, A. The effect of specific background electrolytes on water structure and solute hydration: Consequences for crystal dissolution and growth. *Geochim. Cosmochim. Acta* **2008**, *72*(18), 4476-4487.

[89] Alexander, L. F.; Radacsi, N. Application of electric fields for controlling crystallization. *CrystEngComm* **2019**, *21*(34), 5014-5031.
